# Spatiotemporal dynamics of scattering exceptional points

**DOI:** 10.1038/s41467-026-75187-2

**Published:** 2026-07-07

**Authors:** Yu Xiao, Zhiyuan Che, Lei Shi, Xu Wang, Jie Zhu

**Affiliations:** 1 Institute of Acoustics, School of Physics Science and Engineering, Tongji University, Shanghai, China; 2 State Key Laboratory of Surface Physics, Department of Physics, Fudan University, Shanghai, China; 3 Key Laboratory of Micro- and Nano-Photonic Structures (Ministry of Education), Department of Physics, Fudan University, Shanghai, China; 4 Institute for Nanoelectronic devices and Quantum computing, Fudan University, Shanghai, China; 5 Shanghai Research Center for Quantum Sciences, Shanghai, China

**Keywords:** Topological matter, Fluid dynamics, Acoustics

## Abstract

Exceptional points (EPs) offer new routes to control wave-matter interactions in non-Hermitian systems. Whereas conventional EPs arise in Hamiltonians and mark eigenstate coalescence, scattering EPs recast them in input-output responses for free-space wave control. Yet the rich topology of EPs is largely concealed in steady-state observables, exhibiting similar asymmetric patterns governed by identical Jordan-block forms. Here we exploit scattering as an evolving process in space and time to resolve spatiotemporal dynamics. We establish a cross-domain mapping from momentum-frequency to space-time, connecting static EPs to dynamic scattering singularities. By projecting a unitary scattering matrix onto lower-dimensional subspaces, EP topology is imprinted into a wave packet as a spatiotemporal vortex (STV), whose phase singularity is anchored to the EP and carries its quantized topological charge. In a liquid-surface-wave platform, we observe a charge-1 STV and a disintegrated charge-2 STV, generated by distinct EPs in a single device. Our work links fixed exceptional topology to dynamic wave topology, establishing a framework for spatiotemporal wave control.

## Abstract (web summary)

Exceptional points govern unique wave scattering, yet their topology is often hidden in steady states. Here, the authors map these static singularities to dynamic wave packets, revealing how their topological charge is imprinted onto spatiotemporal vortices using liquid surface waves.

## Introduction

The study of non-Hermitian physics possesses a profound history of over 60 years in quantum research^1,2^. Building upon these quantum origins and through the introduction of parity-time symmetry^3,4^, it has developed into a framework for controlling wave-matter interactions. Recent advances in these non-Hermitian systems with complex eigenvalues have spurred significant interest in exceptional points (EPs)^5,6^, spectral singularities at which eigenvalues and eigenvectors coalesce simultaneously^7–9^. These singularities, carrying exceptional topology, have enabled a host of counterintuitive phenomena, such as PT-symmetric wave manipulation^10,11^, non-Hermitian skin effect^12,13^, and enhanced sensing^14–16^, across diverse platforms including photonics, acoustics, and elastodynamics. Conventionally, these demonstrations predominantly revolve around Hamiltonian EPs (H-EPs), which emerge as degeneracies of the non-Hermitian effective Hamiltonian, associated with the coalescence of intrinsic eigenstates^17,18^. H-EPs, which neglect thermal and quantum jump processes, can be generalized to Liouvillian EPs (L-EPs) in a full quantum description^19–21^. Such L-EPs have been introduced for both Markovian^22^ and non-Markovian^23^ systems, and experimentally demonstrated on various quantum platforms^19,24^. When considering multi-port scattering problems, nevertheless, Hamiltonian could not directly describe the behavior of scattering modes^18^. Open structures such as metagratings^5,25^ are more appropriately modeled by a scattering matrix, which fully characterizes the wave-structure interactions in free space. In light of this, the Hamiltonian-centric view has been fruitfully complemented by the concept of the scattering EP (S-EP)^26,27^.

**Fig. 1:**
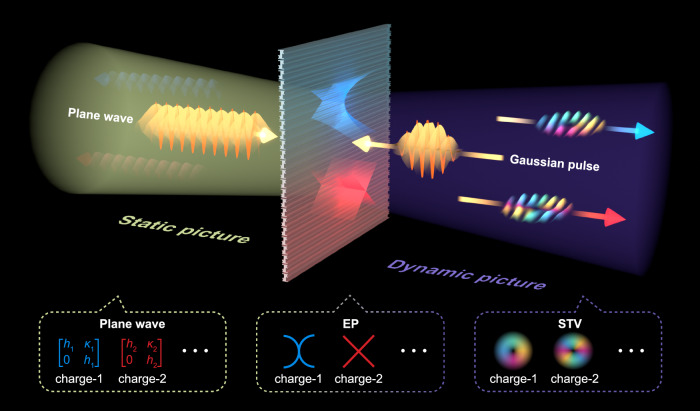
The two facets of scattering EPs. The central metagrating host EPs with distinct topology in its respective subspaces (blue: charge-1; red: charge-2). Left: the static picture. Under steady-state plane wave incidence, both EPs produce similar far-field scattering patterns and share the same Jordan-block matrix forms, hiding their topological differences. Right: the dynamic picture. Under pulsed wave-packet excitation, each EP imprints its topology onto the scattered wave, generating distinct spatiotemporal vortices: a charge-1 STV (blue) and a charge-2 STV (red), as revealed in their phase structures. Note that the left-right division represents different scattering regimes, not different spatial incidence conditions.

S-EPs provide a powerful paradigm for controlling wave scattering, enabling effects such as coherent perfect absorption^18,28^ and reflectionless scattering modes^25,29^. Metagratings, with their versatile capability for wave scattering manipulation^30,31^, allow substantial control over each element of the S-matrix via tailoring every scattering channel. They provide a crucial pathway to precisely synthesize these scattering singularities and have thus emerged as an ideal platform for engineering S-EPs. However, current research—whether on the scattering of plane waves, vortex beams, or polarized waves—has focused exclusively on their steady-state response^25,29,32,33^, leaving the time-evolving interaction of waves and S-EPs largely unexplored. Moreover, from this steady-state perspective, even distinct EPs with fundamentally different topological structures remain largely hidden, exhibiting only similar asymmetric scattering behavior characterized by the same Jordan-block forms (left part of Fig. 1).

The very nature of a scattering event is inherently dynamic and unfolds in both space and time^34^, whereby the perspective of spatiotemporal dynamic offers new degrees of freedom for scattering manipulation^35,36^. In this work, we transcend the steady-state picture of scattering EPs by investigating their spatiotemporal consequences, with these EPs constructed by projecting the full unitary scattering matrix onto lower-dimensional subspaces. We demonstrate that a metagrating designed to host distinct EPs in the momentum-frequency domain can imprint their exceptional topology onto propagating wave packets. Excitation of each static S-EP by a pulsed wave leads to a dynamic scattering singularity in space-time, manifested unequivocally by the formation of a phase vortex in the scattered field—a spatiotemporal vortex (STV) characterized by a quantized topological charge^37–39^. Unlike conventional vortices defined solely in space^40–42^, a STV represents a complex wave packet with intertwined phase singularities in both space and time, carrying transverse orbital angular momentum (OAM). The generation of such intricate waveforms has traditionally been a significant challenge^34,43–51^, making their subsequent control, such as steering the vortex direction, even more difficult. Here, we establish a direct cross-domain correspondence (from momentum-frequency to space-time) between a static EP and the resulting dynamic STV. This connection ensures the STV’s central frequency and wave vector are topologically locked to those of the EP, while its direction can be freely steered by engineering the momentum of the EP within the metagrating. Using a liquid-surface-wave platform, we simultaneously observe a charge-1 STV in the anomalous-transmitted mode and a disintegrated charge-2 STV in the ordinary-transmitted mode, generated by distinct EPs in a single metagrating, thus providing compelling experimental evidence for the spatiotemporal projection of an EP’s topology (right part of Fig. 1).

## Results

### The static picture of EPs—plane wave scattering in the momentum-frequency domain

**Fig. 2:**
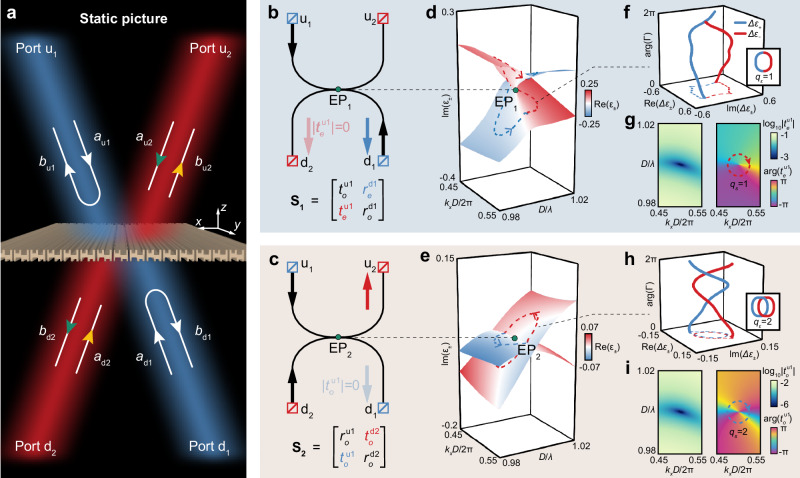
Static picture of scattering EPs under plane-wave excitation. **a** Schematic of the four-port steady-state scattering governed by the full matrix **S**_**p**_ at the target momentum-frequency point (*k*_*x*0_, *ω*_0_). Waves from ports u_1_ or d_1_ are perfectly retroreflected, while those from u_2_ or d_2_ are transmitted to each other across the metagrating. **b**, **c** Inter-port scattering behaviors of the two subsystems described by the sub-matrices **S**_**1**_ and **S**_**2**_, each hosting a projected EP (EP_1_ and EP_2_) at the operating point. **d**, **e** Eigenvalue Riemann surfaces for EP_1_ (**d**) and EP_2_ (**e**), revealing distinct topological structures: a two-sheeted branch cut (EP_1_) and a Dirac-like degeneracy (EP_2_). **f**, **g** Topological characterization of EP_1_. Adiabatic eigenvalue evolution around EP_1_ (**f**) confirms a two-band charge *q*_*ϵ*_ = 1, while the amplitude and phase distributions of the critical matrix element ${t}_{e}^{{{{\rm{u}}}}1}$ (**g**) exhibit a phase vortex with winding number *q*_*s*_ = 1, both validating the topological charge of 1 for EP_1_. **h**, **i** Corresponding analysis for EP_2_ reveals a higher charge of 2, visualized by a Hopf-link braiding (**h**) and a doubly-wound phase structure of the element ${t}_{o}^{{{{\rm{u}}}}1}$ (**i**). All results are obtained from numerical simulations.

We begin by considering a metagrating characterized by broken mirror-*x* and -*z* symmetry, but preserved C_2*y*_ rotational symmetry (Fig. 2a). The metagrating consists of a periodic array of meta-units, each comprising a central transmission channel flanked by two reflective grooves on each surface (see details in Supplementary Notes 1 and 2). The periodicity quantizes the momentum space, discretizing the scattering continuum into distinct diffraction orders. The metagrating operates in a few-diffraction-order regime^52^, where only the zeroth- and first-order transmissions and reflections are propagating, corresponding to ordinary and anomalous scattering, respectively. In this regime, the scattering can be effectively described by a four-port model, even in free space. These ports are defined as: left-upper (port u_1_), right-upper (u_2_), left-lower (d_2_), and right-lower (d_1_), where u (d) denotes the upper (lower) space and the subscripts identify the corresponding ports in that half space.

In the momentum-frequency domain of *k*_*x*_ (the *x*-component wavevector) and *ω* (the angular frequency), the steady-state scattering behavior of the metagrating is governed by

1 $${{{\bf{b}}}}={{{{\bf{S}}}}}_{{{{\bf{p}}}}}({k}_{x},\omega ){{{\bf{a}}}}=\left[\begin{array}{cccc}{r}_{e}^{{{{\rm{u}}}}1}&{r}_{o}^{{{{\rm{u}}}}2}&{t}_{o}^{{{{\rm{d}}}}1}&{t}_{e}^{{{{\rm{d}}}}2}\\ {r}_{o}^{{{{\rm{u}}}}1}&{r}_{e}^{{{{\rm{u}}}}2}&{t}_{e}^{{{{\rm{d}}}}1}&{t}_{o}^{{{{\rm{d}}}}2}\\ {t}_{o}^{{{{\rm{u}}}}1}&{t}_{e}^{{{{\rm{u}}}}2}&{r}_{e}^{{{{\rm{d}}}}1}&{r}_{o}^{{{{\rm{d}}}}2}\\ {t}_{e}^{{{{\rm{u}}}}1}&{t}_{o}^{{{{\rm{u}}}}2}&{r}_{o}^{{{{\rm{d}}}}1}&{r}_{e}^{{{{\rm{d}}}}2}\end{array}\right]{{{\bf{a}}}},$$

where the scattering matrix **S**_**p**_ is composed of reflection and transmission coefficients (denoted *r* and *t*) of ordinary and anomalous scattering modes (identified by subscripts *o* and *e*) under incidence from different ports (see details in Supplementary Note 1). **S**_**p**_ relates the output state ${{{\bf{b}}}}={({b}_{{{{\rm{u}}}}1},{b}_{{{{\rm{u}}}}2},{b}_{{{{\rm{d}}}}1},{b}_{{{{\rm{d}}}}2})}^{{{{\rm{T}}}}}$ to the input ${{{\bf{a}}}}={({a}_{{{{\rm{u}}}}1},{a}_{{{{\rm{u}}}}2},{a}_{{{{\rm{d}}}}1},{a}_{{{{\rm{d}}}}2})}^{{{{\rm{T}}}}}$, both representing the complex pressure amplitudes at the four ports. The specific ordering of input and output states is chosen so that the relevant subspaces are aligned directly to blocks of the full **S**_**p**_ matrix. This simplifies the subsequent analysis by avoiding explicit projection operators^53^.

The four-port scattering matrix **S**_**p**_ in Eq. (1) spans a four-dimensional parameter space that fully characterizes the global scattering behavior of the metagrating. At the operating momentum (${k}_{x0}=\frac{\pi }{D}$, *D* being the period) and frequency (${\omega }_{0}=\frac{2\pi c}{D}$, *c* being the wave speed), the designed metagrating exhibits distinctive steady-state scattering properties: a wave incident from port u_1_ undergoes perfect retroreflection (back along the same port), while incidence from the opposite side of surface normal (u_2_) results in straight transmission to port d_2_, as illustrated in Fig. 2a. The C_2*y*_ rotational symmetry implies that port d_1_ behaves identically to u_1_, and port d_2_ identically to u_2_. Owing to the negligible intrinsic loss of the metagrating, the scattering matrix **S**_**p**_ is unitary, i.e. ${{{{\bf{S}}}}}_{{{{\bf{p}}}}}{{{{{\bf{S}}}}}_{{{{\bf{p}}}}}}^{{{\dagger}} }={{{\bf{I}}}}$ († denoting conjugate transposition). Notably, despite the passive and lossless nature of the metagrating—a condition typically prohibiting the emergence of non-Hermitian degeneracies—projected EPs can be observed within its lower-dimensional subspaces^53^.

To reveal these hidden degeneracies, we project the full scattering matrix onto two distinct subsystems. The first, characterized by the block $${{{{\bf{S}}}}}_{{{{\bf{1}}}}}({k}_{x},\omega )=\left[\begin{array}{cc}{t}_{o}^{{{{\rm{u}}}}1}&{r}_{e}^{{{{\rm{d}}}}1}\\ {t}_{e}^{{{{\rm{u}}}}1}&{r}_{o}^{{{{\rm{d}}}}1}\end{array}\right]$$, describes the scattering from the diagonal space (e.g. ports u_1_ and d_1_) to the lower-half plane (e.g. ports d_1_ and d_2_), as illustrated in Fig. 2b. In this subspace, the scattering behavior is described by **b**_**1**_ = **S**_**1**_**a**_**1**_, where ${{{{\bf{b}}}}}_{{{{\bf{1}}}}}={({b}_{{{{\rm{d}}}}1},{b}_{{{{\rm{d}}}}2})}^{{{{\rm{T}}}}}$ and ${{{{\bf{a}}}}}_{{{{\bf{1}}}}}={({a}_{{{{\rm{u}}}}1},{a}_{{{{\rm{d}}}}1})}^{{{{\rm{T}}}}}$. The second one, given by the block $${{{{\bf{S}}}}}_{{{{\bf{2}}}}}({k}_{x},\omega )=\left[\begin{array}{cc}{r}_{o}^{{{{\rm{u}}}}1}&{t}_{o}^{{{{\rm{d}}}}2}\\ {t}_{o}^{{{{\rm{u}}}}1}&{r}_{o}^{{{{\rm{d}}}}2}\end{array}\right]$$, maps inputs from the left-half plane to outputs in the right-half plane, i.e. **b**_**2**_ = **S**_**2**_**a**_**2**_, where ${{{{\bf{b}}}}}_{{{{\bf{2}}}}}={({b}_{{{{\rm{u}}}}2},{b}_{{{{\rm{d}}}}1})}^{{{{\rm{T}}}}}$ and ${{{{\bf{a}}}}}_{{{{\bf{2}}}}}={({a}_{{{{\rm{u}}}}1},{a}_{{{{\rm{d}}}}2})}^{{{{\rm{T}}}}}$. In our design, the scattering matrices of both subsystems, **S**_**1**_ and **S**_**2**_, are engineered to take the Jordan canonical form at the target operating point, indicating the presence of a second-order EP in each projected subspace (denoted EP_1_ and EP_2_). Remarkably, although these two subsystems exhibit similar steady-state scattering behavior (Figs. 2b, c), both described by the same Jordan form, e.g. $${{{\rm{abs}}}}({{{{\bf{S}}}}}_{{{{\bf{1}}}}})={{{\rm{abs}}}}({{{{\bf{S}}}}}_{{{{\bf{2}}}}})=\left[\begin{array}{cc}0&1\\ 0&0\end{array}\right]$$, their topological characteristics differ fundamentally. Crucially, these specific subspaces are deliberately chosen because they share a common input channel (the top-left port, u_1_). Then, the key elements responsible for the occurrence of the projected EPs (EP_1_ and EP_2_), ${t}_{e}^{{{{\rm{u}}}}1}$ and ${t}_{o}^{{{{\rm{u}}}}1}$, scatter waves from the same shared input (u_1_) to entirely different output channels: ${t}_{e}^{{{{\rm{u}}}}1}$ routes to the bottom-left port (d_2_), while ${t}_{o}^{{{{\rm{u}}}}1}$ routes to the bottom-right port (d_1_). This facilitates the simultaneous and spatially separated experimental observation of EP_1_ and EP_2_.

To unveil the distinct topology of **S**_**1**_ and **S**_**2**_, we examine their eigenvalues *ϵ*_±_ in the momentum-frequency domain. The simulated Riemann surface for EP_1_ of **S**_**1**_ exhibits a classic two-sheeted branch-cut singularity, signifying a conventional second-order EP (Fig. 2d). In contrast, the eigenvalue manifold for EP_2_ of **S**_**2**_ reveals an unconventional, Dirac-like degeneracy topology (Fig. 2e). This marked difference is further quantified by calculating the topological charge carried by each EP via the adiabatic evolution of the eigenvalue differences Δ*ϵ*_±_ ≔ *ϵ*_±_ − *ϵ*_∓_^8,9,54^. When the system parameters encircle EP_1_ along a counterclockwise loop Γ, the two eigenvalues are exchanged after one cycle, and the system returns to its original state only after two cycles (Fig. 2f). This confirms a half-integer single-band topological charge *v*_±(∓)_ = 1/2, yielding a two-band topological charge of *q*_*ϵ*_ = 1, termed a charge-1 EP (see Supplementary Note 4 for details on EP topological charges). For EP_2_, however, the eigenvalue evolution shows that the system recovers its initial state after a single cycle, implying an integer single-band topological charge *v*_±(∓)_ = 1 and hence a two-band charge *q*_*ϵ*_ = 2 (charge-2 EP), as visualized by a Hopf-link braiding structure (Fig. 2h). This unique topology identifies EP_2_ as a Dirac EP formed by the merging of two conventional charge-1 EPs^55,56^, which is protected by C_2*y*_ symmetry (see Supplementary Note 7 for details). We further investigate the behavior of the corresponding eigenstates near the singularities. The phase rigidity strictly approaches zero at both EP_1_ and EP_2_, which is the definitive mathematical hallmark of eigenvector coalescence (see Supplementary Note 8 for details). This also highlights the topological uniqueness of EP_2_: as a Dirac EP, it is cloaked in a diabolic-point^57,58^ facade through its eigenvalue degeneracy, yet retains the nature of an EP through eigenvector coalescence. Note that the observed topological features are tied to the specific choice of subsystem decomposition, as each subsystem corresponds to a distinct set of physical excitation and observation channels. Hence, the existence of EP_1_ and EP_2_ in the specific subspaces implies that their topological features can only be excited by the eigen-input states corresponding to those chosen subsystems.

Importantly, the distinct topology of these EPs can be further elucidated by examining the phase singularities of the matrix elements whose vanishing values are responsible for the formation of the Jordan blocks—specifically, elements ${t}_{e}^{{{{\rm{u}}}}1}$ in **S**_**1**_ and ${t}_{o}^{{{{\rm{u}}}}1}$ in **S**_**2**_. We calculate the amplitude and phase distributions of these elements across the (*k*_*x*_, *ω*) domain within the few-diffraction-order regime. As shown in Figs. 2g and 2i, the amplitude spectra for both ${t}_{e}^{{{{\rm{u}}}}1}$ and ${t}_{o}^{{{{\rm{u}}}}1}$ are nearly identical, reaching zero at the designated EP position (*k*_*x*0_, *ω*_0_). However, their phase maps reveal a profound difference. The phase of ${t}_{e}^{{{{\rm{u}}}}1}$ around EP_1_ exhibits a clear 2*π* winding (Fig. 2g), corresponding to a phase vortex with a topological charge of *q*_*s*_ = 1. In contrast, the phase of ${t}_{o}^{{{{\rm{u}}}}1}$ around EP_2_ shows a doubly-wound 4*π* structure (Fig. 2i), signifying a higher-order phase vortex with charge *q*_*s*_ = 2. Well agreement between theoretical and simulated results is observed (see Supplementary Note 2 for details). These findings provide robust evidence that the topological charges defined by the eigenvalue winding number (*q*_*ϵ*_) and the matrix-element phase winding number (*q*_*s*_) are equivalent, i.e. *q*_*ϵ*_ = *q*_*s*_ (see Supplementary Note 4 for detailed proof), providing a complementary and effective means to characterize the topology of EPs.

### The dynamic picture of EP—EP-STV correspondence in the spatiotemporal domain

Although EP_1_ and EP_2_ possess fundamentally distinct topologies, their far-field scattering is characterized by identical matrix forms—Jordan blocks—leading to similar extreme asymmetric scattering behaviors^25,59^ (conceptually in the left half of Fig. 1, with exemplary data in Fig. 2). This demonstrates that the steady-state response fails to distinguish between distinct scattering EPs. In contrast, the dynamic scattering of a wave packet unlocks a deeper layer of information, allowing the spectral singularity of the EP to imprint its topology directly onto the spatiotemporal structure of the scattered wave. Without loss of generality, we consider the projected matrix **S**_**1**_ as an example (the analysis for **S**_**2**_ follows analogously). As demonstrated, the exceptional topology characterized by the eigenvalue winding number *q*_*ϵ*_ is equivalently encoded in the phase singularity of the key vanishing matrix element, quantified by its phase winding number *q*_*s*_. This equivalence thus provides the pivotal bridge for examining the dynamic behavior of EPs.

Since the matrix element ${t}_{e}^{{{{\rm{u}}}}1}$ of **S**_**1**_ holds phase vortex at (*k*_*x*0_, *ω*_0_), by neglecting the phase anisotropy and distortion, it can be expressed in the vicinity as

2 $${t}_{e}^{{{{\rm{u}}}}1}=f(\rho ){e}^{i{q}_{s}\phi },$$

where *ρ* and *ϕ* are polar coordinates in the momentum-frequency space centered at the EP, defined by $\rho=\sqrt{{k}_{\perp }^{2}+{(\frac{\omega -{\omega }_{0}}{c})}^{2}}$, $\phi={\tan }^{-1}(\frac{\omega -{\omega }_{0}}{c{k}_{\perp }})$, with *f*(0) = 0 pinpointing the phase singularity at EP. Here, *k*_⊥_ is the transverse component of the wavevector of the corresponding channel (see Supplementary Note 5 for details). Now, we consider a wave packet incidence from port u_1_, described by ${P}_{{{{\rm{u}}}}1}^{{{{\rm{in}}}}}={a}_{{{{\rm{u}}}}1}({{{\bf{r}}}},t){e}^{i({{{{\bf{k}}}}}_{{{{\rm{u}}}}1}^{{{{\rm{in}}}}}\cdot {{{\bf{r}}}}-{\omega }_{0}t)}$. Note that *a*_u1_(**r**, *t*) is not a constant amplitude but an envelope, featuring a pulse-like profile in both space and time. Its carrier wave propagates with a wavevector ${{{{\bf{k}}}}}_{{{{\rm{u}}}}1}^{{{{\rm{in}}}}}$, while the envelope itself encompasses a spectrum of plane-wave components around this central direction. Therefore, this incident wave packet can be expressed in a superposition way as ${a}_{{{{\rm{u}}}}1}({{{\bf{r}}}},t)=\iint {\widetilde{a}}_{{{{\rm{u}}}}1}({k}_{\perp },\omega ){e}^{i{k}_{\perp }{r}_{\perp }+i{k}_{\parallel }{r}_{\parallel }-i\omega t}{{{\rm{d}}}}{k}_{\perp }{{{\rm{d}}}}\omega$, where ${\widetilde{a}}_{{{{\rm{u}}}}1}$ is the complex spectrum of wave packet envelope, with a Gaussian amplitude distribution, and *k*_∥_ is the parallel component of the wavevector satisfying ${{k}_{\parallel }}^{2}+{k}_{\perp }^{2}={(\frac{\omega }{c})}^{2}$. The scattering process through ${t}_{e}^{{{{\rm{u}}}}1}$ produces an output wave packet in the corresponding channel (port d_2_) given by ${P}_{{{{\rm{d}}}}2}^{{{{\rm{out}}}}}={b}_{{{{\rm{d}}}}2}({{{\bf{r}}}},t){e}^{i({{{{\bf{k}}}}}_{{{{\rm{d}}}}2}^{{{{\rm{out}}}}}\cdot {{{\bf{r}}}}-{\omega }_{0}t)}$, where ${{{{\bf{k}}}}}_{{{{\rm{d}}}}2}^{{{{\rm{out}}}}}$ is the wavevector aligned with port d_2_. As we proved in the Supplementary Note 4, the scattered envelope, distinct from the simply pulse-like incident one, takes the form

3 $${b}_{{{{\rm{d}}}}2}(\zeta,{r}_{\perp })={e}^{i{\omega }_{0}\zeta }2\pi {i}^{{q}_{s}}{e}^{i{q}_{s}\theta }\int_{0}^{\delta \rho }f(\rho ){J}_{{q}_{s}}(\rho r){\widetilde{a}}_{{{{\rm{u}}}}1}(\rho )\rho {{{\rm{d}}}}\rho$$

where ${J}_{{q}_{s}}$ is the *q*_*s*_th-order Bessel function of the first type, and *δρ* is the upper limit of integration. Note that the output wave packet is now defined in the co-moving coordinate system (*ζ*, *r*_⊥_) tied to the corresponding output port, where $\zeta=\frac{{r}_{\parallel }}{c}-t$ is not merely a spatial dimension, but a hybrid space-time axis tracking the wave packet’s propagation. To characterize the topology of the scattered wave, we further introduce polar coordinate in the (*ζ*, *r*_⊥_) plane in Eq. (3), with polar radius $r=\sqrt{{\zeta }^{2}+{r}_{\perp }^{2}}$ and azimuthal angle $\theta={\tan }^{-1}(\zeta /{r}_{\perp })$.

Remarkably, the topological signature of a static EP, the term ${e}^{i{q}_{s}\phi }$ originally defined in the momentum-frequency domain (Eq. (2)), is now imprinted onto the spatiotemporal profile of a scattered wave packet as ${e}^{i{q}_{s}\theta }$ (Eq. (3)). This provides a definitive signature of a spatiotemporal vortex lying in the co-moving framework. In contrast to conventional spatial vortices, where the phase singularity is defined in a plane orthogonal to the propagation direction^40–42^, the vortex described by Eq. (3) resides in the space-time plane (*ζ*, *r*_⊥_), within which the propagation axis *r*_∥_ itself lies. As a result, the phase singularity in Eq. (3) is not static but dynamically evolves, causing the wave packet to roll like a wheel along its trajectory. The term *θ* directly encapsulates this rolling motion, mixing space (*r*_⊥_) and the propagation coordinate (*ζ*) into a single phase angle. Meanwhile, the integral in Eq. (3) governs the radial structure of the envelope, which does not alter the topological charge *q*_*s*_ defined by the phase winding, thereby ensuring the robustness of the spatiotemporal vortex against perturbations in the packet’s profile (see robustness analysis of charge mapping in Supplementary Note 4). Note that to render the mathematical formalism concise and to make the underlying physical picture of the correspondence intuitive, the derivation above relies on certain approximations, including the local phase-vortex description of the scattering coefficient near the EP and neglecting the anisotropy in the phase distribution. The former is well-justified since the exceptional topologies are strictly localized to the vicinity of the singularity. For the latter, we have further discussed the scattering patterns of the STVs in the presence of anisotropy (see Supplementary Note 4 for details).

**Fig. 3:**
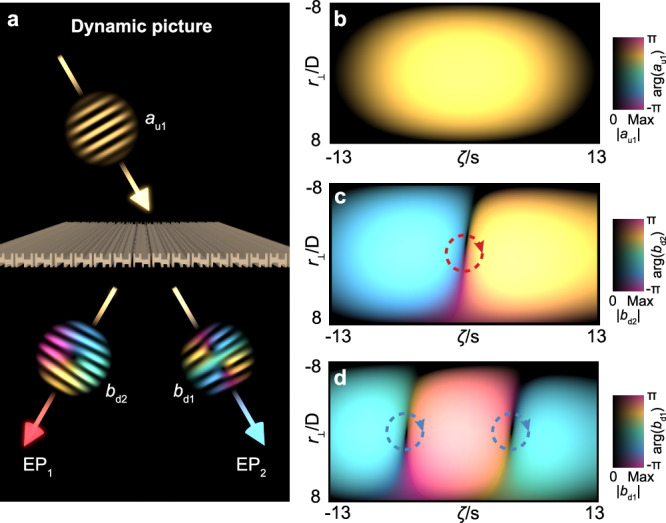
Dynamic picture of scattering EPs under pulsed excitation. **a** Scattering of a Gaussian pulse incident from port u_1_ generates two distinct STVs: a stable charge-1 STV in the anomalous-transmitted mode governed by ${t}_{e}^{{{{\rm{u}}}}1}$ from **S**_**1**_, and a charge-2 STV (metastable, disintegrated into two sequential charge-1 STVs) in the ordinary-transmitted mode governed by ${t}_{o}^{{{{\rm{u}}}}1}$ from **S**_**2**_. **b**–**d** Amplitude (brightness) and phase (color) distributions of the incident pulse envelope *a*_u1_, the anomalous-transmitted envelope *b*_d2_, and the ordinary-transmitted envelope *b*_d1_, in the co-moving space-time coordinates (*ζ*, *r*_⊥_). The field patterns are obtained via the Fourier transform of the simulated ${t}_{e}^{{{{\rm{u}}}}1}$ and ${t}_{o}^{{{{\rm{u}}}}1}$ spectra.

The transition from Eqs. (2) to (3) establishes a direct EP-STV correspondence, enabling the exceptional topology of an EP (with its specific topological charge) to be encoded into the dynamic structure of a STV. We present theoretical results for the designed metagrating, revealing the dynamic consequences of EP_1_ (a charge-1 STV propagating in the anomalous transmission channel) and EP_2_ (a charge-2 STV residing in the ordinary transmission channel) under pulse incidence from port u_1_ (Fig. 3). Remarkably, such a distinctive dynamic picture of EPs (Fig. 3a) is in stark contrast to the widely-explored static picture (Fig. 2a).

Notably, driven by the inherent structural instability of higher-order singularities under unavoidable physical perturbations^38,39^, the genuine charge-2 STV generated by EP_2_ inevitably splits into two stable charge-1 STVs while strictly conserving the global topological charge (see details in Supplementary Note 4). By assuming an incident Gaussian pulse with the amplitude and phase distribution of *a*_u1_ shown in Fig. 3b and performing Fourier transformation on the spectra of the simulated transmission coefficients ${t}_{e}^{{{{\rm{u}}}}1}$ and ${t}_{o}^{{{{\rm{u}}}}1}$, we obtain two theoretically predicted scattered envelope patterns, *b*_d2_ for the anomalous transmission mode and *b*_d1_ for the ordinary mode, confirming the unique spatiotemporal singularities in the (*ζ*, *r*_⊥_) plane (Figs. 3c, d).

As theoretically predicted by Eq. (3) and exemplified by Fig. 3, the EP-STV correspondence enables the realization of an STV whose momentum and frequency are firmly anchored at the EP. By further combining the capacity of metagratings on tailoring EPs in the momentum-frequency domain, this correspondence opens a pathway to fully explore the spatiotemporal signatures of distinct EPs and to achieve powerful control over multiple STVs including their propagation directions and topological charges.

### Experimental observation of the spatiotemporal signatures of EPs

**Fig. 4:**
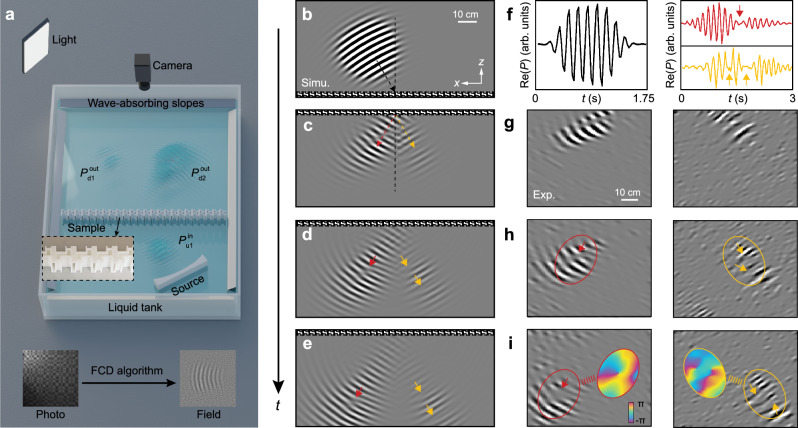
Experimental demonstration of EP-STV correspondence. **a** Schematic of the liquid-surface-wave platform. A metagrating immersed in a tank filled with C_2_H_3_ClF_2_ is illuminated by an incident Gaussian pulse from a concave wave source. A high-speed camera captures the full wave-field evolution, with the surface wave patterns extracted via the FCD algorithm (inset). **b**–**e** Simulated time sequence of field patterns: **b** incident pulse; **c**–**e**, scattered wave packets showing a charge-1 STV in the anomalous-transmitted mode (EP_1_, red arrows) and a disintegrated charge-2 STV in the ordinary-transmitted mode (EP_2_, yellow arrows) at successive time steps. **f** Measured time signals of the incident (black), anomalous-transmitted (red), and ordinary-transmitted (yellow) pulses, acquired at the center of each port. **g**–**i** Experimental field patterns corresponding to **c**–**e** with STVs highlighted by colored circles and arrows. Insets show the measured phase structure of the envelope, confirming the charge-1 vortex (red) and the split charge-2 vortex (yellow).

We provide direct experimental evidence of the spatiotemporal signature of static EP using a liquid-surface-wave platform^60^, the setup of which is shown in Fig. 4a. In the experiment, we investigate the dynamic features of the two static EPs—EP_1_ and EP_2_—associated with the sub-blocks **S**_**1**_ and **S**_**2**_. As established, the exceptional topology of EP_1_ and EP_2_ is encoded in their respective vanishing matrix elements, ${t}_{e}^{{{{\rm{u}}}}1}$ and ${t}_{o}^{{{{\rm{u}}}}1}$, which scatter waves from the same input channel (the upper-left port, u_1_) to different output channels: ${t}_{e}^{{{{\rm{u}}}}1}$ to the lower-left port d_2_, and ${t}_{o}^{{{{\rm{u}}}}1}$ to the lower-right port d_1_. This design spatially separates the transmitted field into distinct left and right sections, enabling independent and simultaneous observation of the topological signatures from different EPs under a single wave-packet incidence, without spatial overlap.

According to our design, under illumination by a Gaussian pulse with a center frequency of 7.03 Hz and an incident angle of 30^°^ (positive to the left of the surface normal), a charge-1 STV emerges in the left half of the transmission side at 30^°^. This corresponds to the anomalous (1st-order) diffraction governed by the input-output relations of ${t}_{e}^{{{{\rm{u}}}}1}$. Simultaneously, a disintegrated charge-2 STV appears in the right half at −30^°^, corresponding to the ordinary (0th-order) diffraction via ${t}_{o}^{{{{\rm{u}}}}1}$ (simulated results in Fig. 4b-e). Figure 4f shows the experimental time-domain signals of the incident pulse (the left panel), the 1st-order transmitted pulse (right-upper), and the 0th-order transmitted pulse (right-lower), measured at the central points of their respective spatially separated ports (u_1_, d_2_, d_1_). In contrast to the simple Gaussian profile of the incident signal, the scattered pulses clearly exhibit the topological imprint of the static EPs: a single dip observed in the right-upper panel originating from EP_1_ of **S**_**1**_, and two consecutive dips in the right-lower panel imprinted by EP_2_ of **S**_**2**_. Notably, the advantage of our experimental system lies in its ability to capture the evolution of the scattered wave packet in spatiotemporal coordinates (*ζ*, *r*_⊥_), whether on spatial cross-sections (fixed *t*) or space-time cross-sections (fixed *r*_∥_). The measured transmitted patterns, in left- and right-half of the lower plane, illustrate the propagation of the singularities carried by the STVs (Figs. 4g-i). The insets in Fig. 4i present the phase maps of the spatial envelope around the singularity, confirming a single charge-1 STV (reflecting the topology of EP_1_) and two sequential charge-1 STVs (reflecting the topology of EP_2_). Spatial sequences of transmitted waves in the space-time domain and the full scattering behavior of the total experimental system are provided in the Supplementary Note 6. We also extract the topological charge of scattering pulses from the experimental results, quantitatively demonstrating the topological features of the STVs in both the purely spatial and the mixed spatiotemporal domains (see Supplementary Note 6 for details). The overall agreement between numerical simulations and experimental results is evident. These observations provide definitive evidence of the established EP-STV correspondence across domains (from frequency-momentum space to spatial-temporal domain), thereby validating the dynamic picture of EPs.

## Discussion

To conclude, we have demonstrated that the spatiotemporal scattering dynamics of wave packets can reveal hidden topological information of static EPs, which is elusive in conventional steady-state field observations. We establish a direct correspondence between a scattering EP in the momentum-frequency domain and the STV it generates. This connection, rigorously derived and experimentally verified, goes beyond the conventional steady-state perspective and provides a new paradigm for probing dynamic non-Hermitian topology. Notably, the resulting STVs inherit the salient feature of EPs, i.e., simultaneous topological robustness and high sensitivity: while STVs corresponding to EP_1_ and EP_2_ exhibit pronounced spectral shifts in response to external perturbations, their centers are firmly anchored together at the updated location, preserved by C_2_ symmetry without being destroyed. Combined with the flexible wave-manipulation capability of metagratings, this approach significantly enriches both the topological properties and steering capabilities of STVs. Furthermore, while the non-Hermiticity in this work arises from projecting a unitary scattering matrix, introducing controlled genuine losses could unlock a profoundly richer landscape of spectral degeneracies. Such lossy systems would support global higher-order EPs that map onto even more complex spatiotemporal dynamics under wave-packet incidence, including higher-charge STVs, STV clusters, or interacting vortex networks. Our findings pave the way for exploiting fixed structures to control dynamic topological waves in space and time, with potential implications for wave routing, sensing, and fundamental studies in diverse physical systems.

## Methods

### Theory and simulation of liquid surface wave

In the liquid-surface wave system, surface wave propagation obeys a nonlinear dispersion relation^61^, which differs from the linear cone-type dispersion found in systems such as acoustics or electromagnetism in free space. The dispersion relation is given by ${\omega }^{2}=\left(gk+\frac{\sigma {k}^{3}}{\rho }\right)\tanh (k{h}_{0})$, where *ω* is the angular frequency, *g* is the gravitational acceleration (9.8067 m/s^2^), *σ* is the surface tension (0.0187 N/m for C_2_H_3_ClF_2_), *ρ* is the liquid density (1249 kg/m^3^), and *h*_0_ is the undisturbed liquid depth (2.5 cm). In our experiments, we consider small wave amplitudes (much smaller than the wavelength) and a low-frequency range (6–10 Hz) to minimize nonlinear and capillary effects.

### Numerical methods to obtain exceptional topologies, spectrum of transmitted coefficients and corresponding STVs

Under liquid-surface-wave dispersion conditions, the scattering behaviors of the metagrating under different incident conditions, corresponding to different *k*_*x*_ and *ω*, were obtained using the frequency-domain solver in the finite-element software COMSOL Multiphysics. Floquet periodic boundary conditions were applied to both sides of the metagrating, and perfectly matched layers were implemented at the top and bottom of the computational domain. Through Fourier analysis of the scattering field, the reflection and transmission coefficients of each diffraction order of the metagrating were extracted, from which the total scattering matrix **S**_**p**_ was derived. Projection of **S**_**p**_ onto the specific subsystems **S**_**1**_ and **S**_**2**_ yields the eigenvalue topologies associated with EP_1_ and EP_2_. Using the transient solver in COMSOL Multiphysics, a Gaussian excitation pulse was configured with a center frequency and incident angle matched to the operating point (*k*_*x*0_, *ω*_0_), and an appropriate simulation time range was defined. The resulting time-domain evolution was then visualized from pulse incidence to scattering, demonstrating the STVs in the anomalous and specular channels induced by EP_1_ and EP_2_.

The theoretical results were obtained using coupled-mode theory, described in Supplementary Note 2. Further details about numerical simulation are provided in Supplementary Note 5.

### Experimental setup and method to visualize STVs

The experimental setup consists of a metagrating fabricated by resin material, designed to realize the required scattering matrix **S**_**p**_, and immersed in a liquid tank filled with C_2_H_3_ClF_2_. Wave-absorbing slopes are installed at the tank boundaries to suppress unwanted reflections. A concave wave source generates an incident Gaussian pulse covering the operational momentum-frequency range, with its customized curvature shaping the desired spatial wavefront. A high-speed camera captures the full wave-field evolution with high spatial and temporal resolution. The surface wave patterns are then extracted using a Fast Checkerboard Demodulation (FCD) algorithm^62^ for precise signal analysis (inset in Fig. 4a).

In experiments, we employ an incident Gaussian pulse with a waist of 6.8 cm and a duration of 0.3 s. Owing to perturbations from the sample, environment, and the inherent sensitivity of EPs, the operational parameters shift slightly: angle and center frequency for EP_1_ are adjusted to 32^°^ and 6.60 Hz, while for EP_2_ they become 32^°^ and 6.83 Hz, respectively. All experimental data are bandpass-filtered for enhanced clarity.

## Supplementary information

Supplementary Information

Description of Additional Supplementary Files

Supplementary Data 1-11

Transparent Peer Review file

## Data Availability

The data supporting the findings of this study are available within the main text and Supplementary Information. The data of experimental graph are available in Supplementary Data. Additional data are available from the corresponding author upon request.
