# Supplementary material for: Spatiotemporal dynamics of scattering exceptional points
Source: Nat Commun. 2026 Jul 7;17:8373. doi: 10.1038/s41467-026-75187-2 (PMC13473472; doi:10.1038/s41467-026-75187-2)
Supplement: Supplementary file 1 [file 41467_2026_75187_MOESM1_ESM.pdf]

Supplementary Information for Spatiotemporal Dynamics of Scattering Exceptional Points

Yu Xiao[1,#], Zhiyuan Che[1,2,3,#], Lei Shi[2,3,4,5,*], Xu Wang[1,*], & Jie Zhu[1,*]

[1]*Institute of Acoustics, School of Physics Science and Engineering, Tongji University, Shanghai, 200092, China*

[2]*State Key Laboratory of Surface Physics, Department of Physics, Fudan University, Shanghai, 200433, China*

[3]*Key Laboratory of Micro- and Nano-Photonic Structures (Ministry of Education), Department of Physics, Fudan University, Shanghai, 200433, China*

[4]*Institute for Nanoelectronic devices and Quantum computing, Fudan University, Shanghai 200438, China*

[5]*Shanghai Research Center for Quantum Sciences, Shanghai, 201315, China*

# Contributed equally to this work

** Corresponding author*

E-mail: lshi@fudan.edu.cn; xuwang@tongji.edu.cn; jiezhu@tongji.edu.cn

Contents

Supplementary Note 1. S-matrix of metagrating defining in momentum-frequency space

Supplementary Note 2. Coupled mode theory (CMT)

Supplementary Note 3. Constructing rule of projected EP

Supplementary Note 4. Derivation about EP-STV correspondence

Supplementary Note 5. Details of simulation and experiment setups

Supplementary Note 6. Additional experimental results

Supplementary Note 7. Evolution of projected EPs governed by C_2-symmetry perturbations

Supplementary Note 8. Eigenvector coalescence of EP_1 and EP_2

Supplementary Note 9. Extended simulation in acoustic system

Supplementary References

1 Supplementary Note 1. S-matrix of metagrating defining in momentum-frequency space

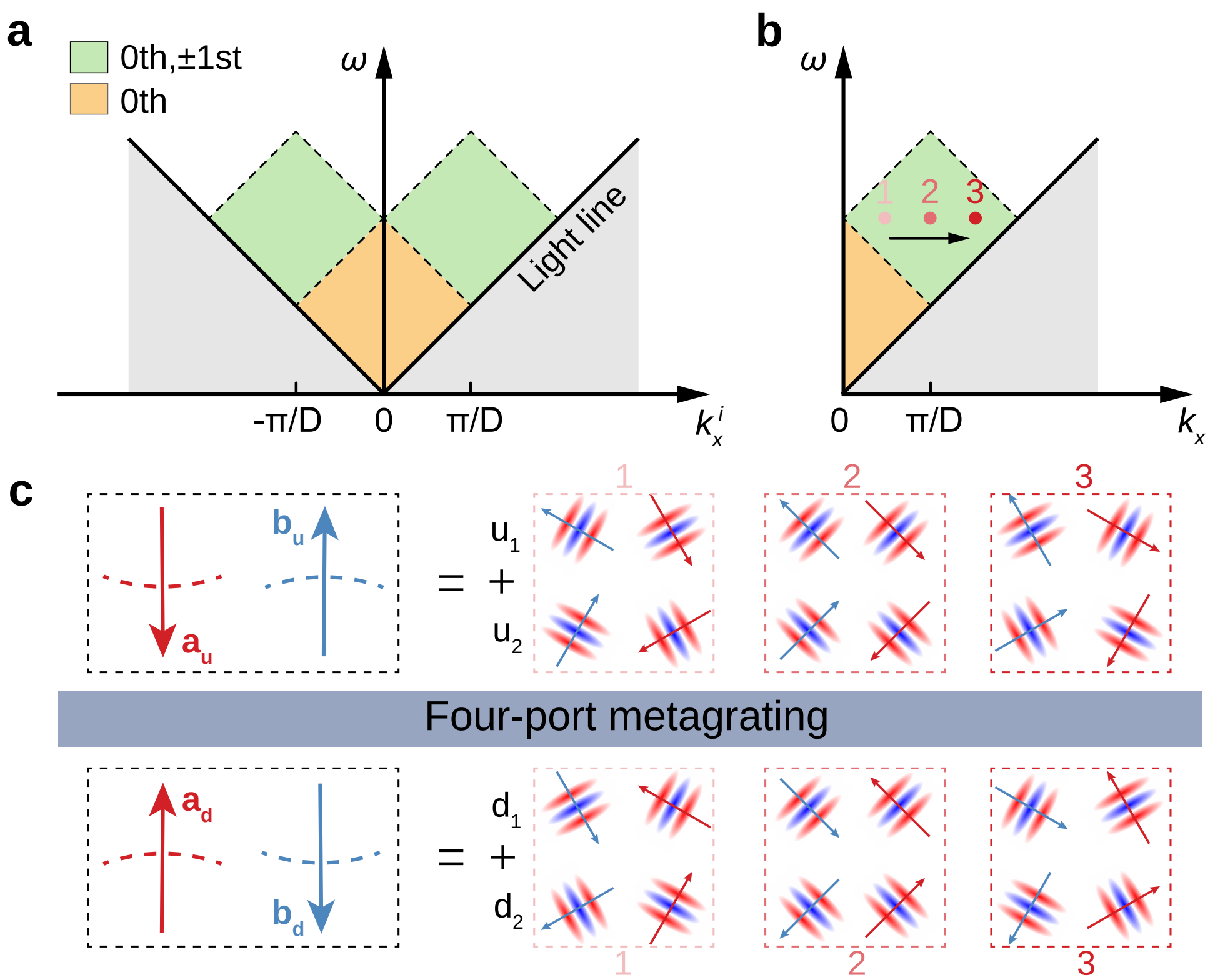

Supplementary Figure 1. **a**, Diffraction order chart, the gray region denotes the evanescent regime where only surface modes exist, the yellow region indicates the regime supporting solely 0th-order diffraction modes, and the green region represents the regime permitting both 0th- and $\pm$1st- order diffraction modes. **b**, The coordinates transformation from k_x^i to k_x. **c**, The input-output scattering behaviors for exemplary points 1-3 marked in **b**.

We start by a general framework of 2-dimensional (2D) metagrating, which is constructed by a periodic grating in x-direction, and open in z-direction. With the plane-wave input, the metagrating becomes a multi-port scattering system consisting of different diffraction modes above and below the metagrating. The scattering behaviors of this system could be described by the grating equation:

$$k_x^m = k_x^i + mG, \tag{S1}$$

where m represents the order of diffraction modes ($m = 0, \pm1, \cdots$), k_x^i and k_x^m are the x-

component wave vector of input wave and mth-order output wave (including reflection and transmission), and $G = \frac{2\pi}{D}$ is the amplitude of reciprocal lattice vector (D being the lattice constant). The diffraction modes of the metagrating are divided into propagable modes within scattering continuum (the z-component wave vector of mth-order output wave $k_z^m = \sqrt{k^2 - (k_x^m)^2}$ is real, k is the wavenumber of input wave, shown by the region above the light line in Supplementary Fig. 1a) and evanescent modes existing on structural surface (k_z^m is imaginary, shown by the gray region below the light line in Supplementary Fig. 1a). We focus solely on propagable modes, which is divided into different cones by Wood's anomaly (WA) lines $\omega = c(\frac{m}{|m|}k_x^i + |m|G)$ displayed in Supplementary Fig. 1a, c is the velocity of plane wave and ω is angular frequency. The mth-order diffraction modes arise at the cone above the mth WA line, constructing the scattering ports. The number of scattering ports N is determined by the number of cones on the same horizontal line within diffraction order chart. We assume the plane-wave input from all ports (regarding the right-most cone as the first port), thereby, the total pressure field above and below the metagrating can be described by

$$\begin{aligned} P_{\mathrm{u}}(x,z) &= \sum_{n=1}^{N}(a_{\mathrm{u}n}\frac{e^{ik_z^n z}}{\sqrt{k_z^n}} + b_{\mathrm{u}n}\frac{e^{-ik_z^n z}}{\sqrt{k_z^n}})e^{ik_x^n x} \text{ (upper space)} \\ P_{\mathrm{d}}(x,z) &= \sum_{n=1}^{N}(a_{\mathrm{d}n}\frac{e^{-ik_z^n z}}{\sqrt{k_z^n}} + b_{\mathrm{d}n}\frac{e^{ik_z^n z}}{\sqrt{k_z^n}})e^{ik_x^n x} \text{ (lower space)} \end{aligned} \quad \text{(S2)}$$

where $a_{\mathrm{u(d)}n}$ and $b_{\mathrm{u(d)}n}$ are the input and output complex amplitudes of port-n plane wave at upper (lower) half-space. $k_z^n = \sqrt{k^2 - (k_x^n)^2}$ and $k_x^n = k_x - (n-1)G$ are z- and x-component wave vectors of port-n plane-wave modes. $k_x \in [\frac{(N-2)G}{2}, \frac{NG}{2}]$ is the common input wave vector shared by all scattering ports as a port-dependent momentum. $\frac{1}{\sqrt{k_z^n}}$ is a normalized factor to ensure the energy conservation of the input-output process and the unity of system without loss/gain [1], that is, η (energy efficiency) $= \frac{\sum_{n=1}^{N}(|b_{\mathrm{u}n}|^2+|b_{\mathrm{d}n}|^2)/k_z^n}{\sum_{n=1}^{N}(|a_{\mathrm{u}n}|^2+|a_{\mathrm{d}n}|^2)/k_z^n} = 1$.

For simplicity, we set the port number $N = 2$ ($n = 1, 2$), thus we construct a few-diffraction-order metagrating so that only ordinary (port-switching) and extraordinary (port-preserving) scattering should be considered, in which $k_x \in [0, 2\pi/D]$, as demonstrated in main text. Considering the full space, a four-port scattering system could be defined in momentum space spanned by k_x and ω shown in Supplementary Fig. 1b: $\mathbf{b}(k_x, \omega) = \mathbf{S}(k_x, \omega) \cdot \mathbf{a}(k_x, \omega)$, where $\mathbf{a} = (\mathbf{a_u}, \mathbf{a_d})^{\mathrm{T}} = (a_{\mathrm{u}1}, a_{\mathrm{u}2}, a_{\mathrm{d}1}, a_{\mathrm{d}2})^{\mathrm{T}}$ and $\mathbf{b} = (\mathbf{b_u}, \mathbf{b_d})^{\mathrm{T}} = (b_{\mathrm{u}1}, b_{\mathrm{u}2}, b_{\mathrm{d}1}, b_{\mathrm{d}2})^{\mathrm{T}}$ are 4D column vectors, demonstrating the input-output process between four ports. $\mathbf{S}(k_x, \omega)$ could be written by a matrix composed by four sub-blocks: $\begin{bmatrix} \mathbf{r^u} & \mathbf{t^d} \\ \mathbf{t^u} & \mathbf{r^d} \end{bmatrix}$, where $\mathbf{r^{u(d)}} = \begin{bmatrix} r_e^{\mathrm{u1(d1)}} & r_o^{\mathrm{u2(d2)}} \\ r_o^{\mathrm{u1(d1)}} & r_e^{\mathrm{u2(d2)}} \end{bmatrix}$ and $\mathbf{t^{u(d)}} = \begin{bmatrix} t_o^{\mathrm{u1(d1)}} & t_e^{\mathrm{u2(d2)}} \\ t_e^{\mathrm{u1(d1)}} & t_o^{\mathrm{u2(d2)}} \end{bmatrix}$. The entries r and t specify the ordinary/extraordinary (subscript o/e) reflection and transmission coefficient under the input of port u_1, u_2, d_1 and d_2. The scattering behaviors of this scattering system is illustrated in Supplementary Fig. 1c. To describe the four-port metagrating in detail when k_x changes in it's domain, we select three exemplary points denoted in Supplementary Fig. 1b and exhibit their input-output scattering behaviors of all ports in Supplementary Fig. 1c. One could observe that only when $k_x = G/2$ the input and output paths of each port coincide. When k_x deviates from $G/2$, the input and output path of each port undergo deflection, yet they still maintain mathematical consistency between four inputs with four outputs.

2 Supplementary Note 2. Coupled mode theory (CMT)

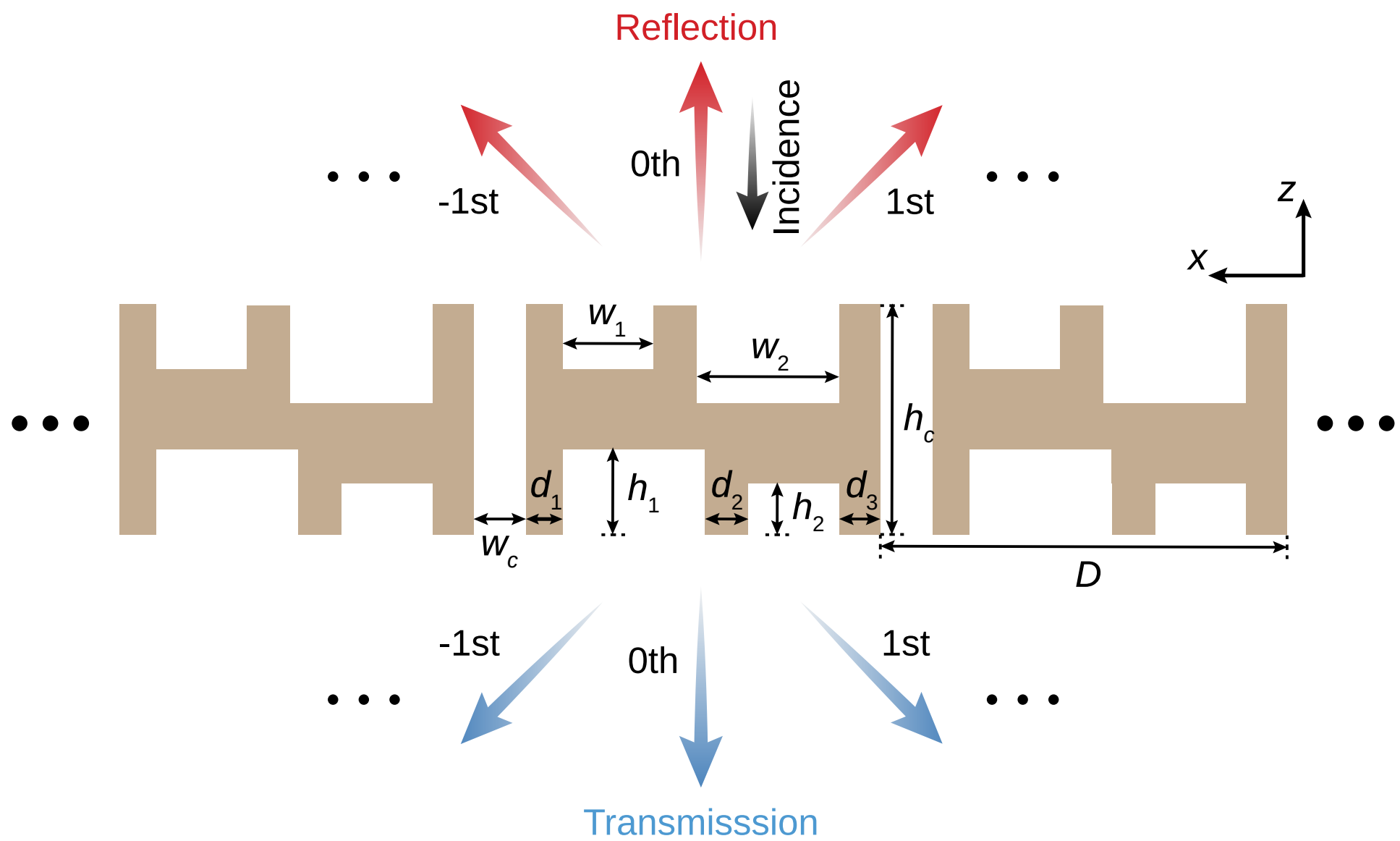

Supplementary Figure 2. Schematic of a zigzag passive-lossless metagrating where CMT is used to solve the coefficients of each reflection and transmission modes.

Supplementary Fig.2 shows the schematic of the zigzag passive-lossless metagrating in main text, and the geometric parameters of which are listed in Table 1. For solving the scattering behaviors of this metagrating system, CMT could be used [2]. Firstly, we rewrite the Eq. S2 as the diffraction-modes-form:

$$p^{\mathrm{u(d)}}(x,z) = \sum_m e^{ik_x^m x}[\Psi_m^{\mathrm{u(d)}+} e^{ik_z^m z} + \Psi_m^{\mathrm{u(d)}-} e^{-ik_z^m z}], \quad \text{(S3)}$$

where $\Psi_m^{\mathrm{u}+(-)}$ and $\Psi_m^{\mathrm{d}-(+)}$ are the normalized amplitude of mth-order input (output) wave pressure from upside and downside ports, and the superscript $+$ $(-)$ indicates downward- (upward-) propagating waves. The input-output relationship is $\Psi_m^{\mathrm{u}-} = r_m^{\mathrm{u}} \Psi_m^{\mathrm{u}+} + t_m^{\mathrm{d}} \Psi_m^{\mathrm{d}-}$ and $\Psi_m^{\mathrm{d}+} = t_m^{\mathrm{u}} \Psi_m^{\mathrm{u}+} + r_m^{\mathrm{d}} \Psi_m^{\mathrm{d}-}$, $r_m^{\mathrm{u(d)}}$ and $t_m^{\mathrm{u(d)}}$ indicate the mth order reflection and transmission coefficients of upper (lower) half-space. $k_x^m = k \sin\theta_i + mG$, θ_i is the incident angle. We consider only a plane wave impinges upon the metagrating from the upside port, that is, $\Psi_0^{\mathrm{u}+} = 1$ but $\Psi_{\pm1,\pm2,\cdots}^{\mathrm{u}+} = 0$, and

$\Psi_m^{\mathrm{d}-} = 0$ for all m. Obviously we can control the number of propagable reflected and transmitted modes by tuning k, θ_i and D. If only four propagable modes exist: two reflected modes at upper space and two transmitted modes at lower space, the four-port scattering system in main text is established.

By the relation $v = \frac{-1}{i\omega\rho}\frac{\partial p}{\partial z}$, the velocity field in z-direction are described as:

$$v(x,z) = \frac{-1}{\omega\rho}\sum_m k_z^m e^{ik_x^m x}[\Psi_m^{\mathrm{u(d)}+} e^{ik_z^m z} - \Psi_m^{\mathrm{u(d)}-} e^{-ik_z^m z}], \tag{S4}$$

where ρ is the density of background medium.

The wave pressure and z-component velocity field inside the closed-end groove and transmitted channel are the superposition of multiple waveguide modes:

$$p_c(x,z) = \sum_s \cos\alpha_{sc}(x - x_c)[H_{sc}^+ e^{i\beta_{sc}z} + H_{sc}^- e^{-i\beta_{sc}z}], \tag{S5}$$

$$v_c(x,z) = \frac{-\beta_{sc}}{\omega\rho}\sum_s \cos\alpha_{sc}(x - x_c)[H_{sc}^+ e^{i\beta_{sc}z} - H_{sc}^- e^{-i\beta_{sc}z}] \tag{S6}$$

and

$$p_l^{\mathrm{u(d)}}(x,z) = \sum_s \cos\alpha_{sl}(x - x_l^{\mathrm{u(d)}})[H_{sl}^{\mathrm{u(d)}+} e^{i\beta_{sl}z} + H_{sl}^{\mathrm{u(d)}-} e^{-i\beta_{sl}z}], \tag{S7}$$

$$v_l^{\mathrm{u(d)}}(x,z) = \frac{-\beta_{sl}}{\omega\rho}\sum_s \cos\alpha_{sl}(x - x_l^{\mathrm{u(d)}})[H_{sl}^{\mathrm{u(d)}+} e^{i\beta_{sl}z} - H_{sl}^{\mathrm{u(d)}-} e^{-i\beta_{sl}z}] \tag{S8}$$

where p_c and v_c ($p_l^{\mathrm{u(d)}}$ and $v_l^{\mathrm{u(d)}}$) are the wave pressure and z-component velocity field inside the channel (lth groove of upper (lower) surface), $H_{sc}^{\pm}$ ($H_{sl}^{\pm}$) is the amplitude of the s-th wave guide mode along $\mp z$ direction(normal direction), $\alpha_{sc}(\alpha_{sl})$ and $\beta_{sc}(\beta_{sl})$ indicate the x- and z-component wave vectors of the s-th wave guide mode inside the channel (l-th groove). $\alpha_{sc} = s\pi/w_c$ ($\alpha_{sl} = s\pi/w_l^{\mathrm{u(d)}}$), $\beta_{sc} = \sqrt{k^2 - \alpha_{sc}^2}$ ($\beta_{sl} = \sqrt{k^2 - \alpha_{sl}^2}$). w_c ($w_l^{\mathrm{u(d)}}$) and x_c ($x_l^{\mathrm{u(d)}}$) denote the width and initial position of the channel (lth groove at upper (lower) surface).

Leveraging the continuous boundary conditions of pressure field and normal velocity at the interface between upper and lower half-spaces with the channel (grooves):

$$p^{\mathrm{u(d)}} = \begin{cases} p_c & x_c < x < x_c + w_c, \\ p_l^{\mathrm{u(d)}} & x_l^{\mathrm{u(d)}} < x < x_l^{\mathrm{u(d)}} + w_l^{\mathrm{u(d)}}, l = 1, \cdots, L, \end{cases} \tag{S9}$$

$$v^{\mathrm{u(d)}} = \begin{cases} v_c & x_c < x < x_c + w_c, \\ v_l^{\mathrm{u(d)}} & x_l^{\mathrm{u(d)}} < x < x_l^{\mathrm{u(d)}} + w_l^{\mathrm{u(d)}}, l = 1, \cdots, L, \\ 0 & \text{other regions.} \end{cases} \tag{S10}$$

We firstly consider the boundary condition at the upside surface. Setting $z = 0$ at the upside surface, and substituting Eqs. S3-S8 into the first expression of Eqs. S9-S10, we could obtain the following equations after multiplying by orthogonal waveguide modes:

$$M_{11}^{C}(\mathbf{\Psi}^{\mathrm{u}+} + \mathbf{\Psi}^{\mathbf{u}-}) = M_{21}^{C}(\mathbf{H}_{\mathbf{C}}^{+} + \mathbf{H}_{\mathbf{C}}^{-}) \tag{S11}$$

and

$$M_{12}^{G\mathrm{u}}(\mathbf{\Psi}^{\mathbf{u}+} + \mathbf{\Psi}^{\mathbf{u}-}) = M_{22}^{G\mathrm{u}}(\mathbf{H}_{\mathbf{G}}^{\mathrm{u}+} + \mathbf{H}_{\mathbf{G}}^{\mathbf{u}-}), \tag{S12}$$

where $\mathbf{\Psi}^{\mathbf{u}\pm} = [\Psi_{-M}^{\mathrm{u}\pm}, \cdots, \Psi_{m}^{\mathrm{u}\pm}, \cdots, \Psi_{M}^{\mathrm{u}\pm}]^T$ represents the amplitude of $2M + 1$ orders incident and output (reflection plus transmission) wave pressure, $\mathbf{H}_{\mathbf{c}}^{\pm} = [H_{1c}^{\pm}, \cdots, H_{sc}^{\pm}, \cdots, H_{Sc}^{\pm}]^T$ and $\mathbf{H}_{\mathbf{G}}^{\mathbf{u}\pm} = [\mathbf{H}_{\mathbf{1}}^{\mathbf{u}\pm}, \cdots, \mathbf{H}_{\mathbf{l}}^{\mathbf{u}\pm}, \cdots, \mathbf{H}_{\mathbf{L}}^{\mathbf{u}\pm}]^T$ ($\mathbf{H}_{\mathbf{l}}^{\mathbf{u}\pm} = [H_{1l}^{\mathrm{u}\pm}, \cdots, H_{sl}^{\mathrm{u}\pm}, \cdots, H_{Sl}^{\mathrm{u}\pm}]^T$) represent the amplitude of wave guide mode inside channel and grooves, S and L are the number of waveguide modes and grooves. $M_{12}^{G\mathrm{u}} = [M_{12}^{1\mathrm{u}}, \cdots, M_{12}^{l\mathrm{u}}, \cdots, M_{12}^{L\mathrm{u}}]^T$ and $M_{22}^{G\mathrm{u}} = \begin{bmatrix} M_{22}^{1\mathrm{u}} & 0 & \cdots & \cdots & 0 \\ 0 & \ddots & 0 & \cdots & \vdots \\ \vdots & 0 & M_{22}^{l\mathrm{u}} & 0 & \vdots \\ \vdots & \cdots & 0 & \ddots & 0 \\ 0 & \cdots & 0 & & M_{22}^{L\mathrm{u}} \end{bmatrix}$,

where the elements are described as

$$
\begin{aligned}
M_{11}^{C}(s,m) &= \frac{1}{w_c}\int_{x_c}^{x_c+w_c} e^{ik_x^m x}\cos\alpha_{sc}(x-x_c)\mathrm{d}x,\\
M_{12}^{l\mathrm{u}}(s,m) &= \frac{1}{w_l^{\mathrm{u}}}\int_{x_l^{\mathrm{u}}}^{x_l^{\mathrm{u}}+w_l^{\mathrm{u}}} e^{ik_x^m x}\cos\alpha_{sl}(x-x_l^{\mathrm{u}})\mathrm{d}x,
\end{aligned}
\tag{S13}
$$

$$
\begin{aligned}
M_{21}^{C}(k_1,k_2) &= \frac{1}{w_c}\int_{x_c}^{x_c+w_c} \cos\alpha_{k_1c}(x-x_c)\cos\alpha_{k_2c}(x-x_c)\mathrm{d}x,\\
M_{22}^{l\mathrm{u}}(k_1,k_2) &= \frac{1}{w_l^{\mathrm{u}}}\int_{x_l^{\mathrm{u}}}^{x_l^{\mathrm{u}}+w_l^{\mathrm{u}}} \cos\alpha_{k_1l}(x-x_l^{\mathrm{u}})\cos\alpha_{k_2l}(x-x_l^{\mathrm{u}})\mathrm{d}x,
\end{aligned}
\tag{S14}
$$

$k_{1(2)} = 1,\cdots,K$. Substituting Eqs. S3-S8 into the second expression of Eqs. S9-S10, after multiplying by orthogonal plane-wave harmonic modes we can obtain:

$$
N_1(\mathbf{\Psi}^{\mathrm{u}+} - \mathbf{\Psi}^{\mathbf{u}-}) = N_{21}^{C}(\mathbf{H}_{\mathbf{C}}^{+} - \mathbf{H}_{\mathbf{C}}^{-}) + N_{22}^{G\mathrm{u}}(\mathbf{H}_{\mathbf{G}}^{\mathbf{u}+} - \mathbf{H}_{\mathbf{G}}^{\mathbf{u}-}),
\tag{S15}
$$

where $N_{22}^{G\mathrm{u}} = [N_{22}^{1\mathrm{u}},\cdots,N_{22}^{l\mathrm{u}},\cdots,N_{22}^{L\mathrm{u}}]$. The elements of Eq. S15 are derived as

$$
N_1(m,m_1) = -\frac{k_{z,m}}{D}\int_0^D e^{i(k_{x,m}-k_{x,m_1})x}\mathrm{d}x,
\tag{S16}
$$

$$
\begin{aligned}
N_{21}^{C}(m,s) &= -\frac{\beta_{sc}}{D}\int_{x_c}^{x_c+w_c}\cos\alpha_{sc}(x-x_c)e^{-ik_x^m x}\mathrm{d}x,\\
N_{22}^{l\mathrm{u}}(m,s) &= -\frac{\beta_{sl}}{D}\int_{x_l^{\mathrm{u(d)}}}^{x_l^{\mathrm{u}}+w_l^{\mathrm{u}}}\cos\alpha_{sl}(x-x_l^{\mathrm{u}})e^{-ik_x^m x}\mathrm{d}x,
\end{aligned}
\tag{S17}
$$

$m \in 2M+1$ denotes the order of reflected (transmitted) modes, and $m_1 \in 2M+1$ as the orthogonal function of m. Similarly, setting $z = 0$ at the downside surface, and substituting Eqs. S3-S8 into Eqs. S9-S10, we get the equations like above forms:

$$
M_{11}^{C}(\mathbf{\Psi}^{\mathbf{d}+} + \mathbf{\Psi}^{\mathbf{d}-}) = M_{21}^{C}(\mathbf{H}_{\mathbf{C}}^{\prime +} + \mathbf{H}_{\mathbf{C}}^{\prime -})
\tag{S18}
$$

$$
M_{12}^{G\mathrm{d}}(\mathbf{\Psi}^{\mathbf{d}+} + \mathbf{\Psi}^{\mathbf{d}-}) = M_{22}^{G\mathrm{d}}(\mathbf{H}_{\mathbf{G}}^{\mathbf{d}+} + \mathbf{H}_{\mathbf{G}}^{\mathbf{d}-}),
\tag{S19}
$$

$$
N_1(\mathbf{\Psi}^{\mathbf{d}+} - \mathbf{\Psi}^{\mathbf{d}-}) = N_{21}^{C}(\mathbf{H}_{\mathbf{C}}^{\prime +} - \mathbf{H}_{\mathbf{C}}^{\prime -}) + N_{22}^{G\mathrm{d}}(\mathbf{H}_{\mathbf{G}}^{\mathbf{d}+} - \mathbf{H}_{\mathbf{G}}^{\mathbf{d}-}).
\tag{S20}
$$

$M_{12}^{G\mathrm{d}}$, $M_{22}^{G\mathrm{d}}$, and $N_{22}^{G\mathrm{d}}$ share the similar mathematical forms with $M_{12}^{G\mathrm{u}}$, $M_{22}^{G\mathrm{u}}$, and $N_{22}^{G\mathrm{u}}$, except that the parameters are replaced with those at the lower surface. $\mathbf{H}_{\mathbf{G}}^{\mathbf{d}\pm}$ and $\mathbf{H}_{\mathbf{C}}^{\prime\pm}$ are the amplitude of

waves at the downside surface within the grooves and channel, which is related with $\mathbf{H_G^{u\pm}}$ and $\mathbf{H_C^{\pm}}$ by the phase matrices T_c and $T_l^{\mathrm{u(d)}}$:

$$\mathbf{H_C'^{+}} = \mathbf{T_c} \cdot \mathbf{H_C^{+}}, \mathbf{H_C^{-}} = \mathbf{T_c} \cdot \mathbf{H_C'^{-}}, \tag{S21}$$

$$\mathbf{H_l^{u-}} = \mathbf{T_l^{u}} \cdot \mathbf{H_l^{u+}}, \mathbf{H_l^{d+}} = \mathbf{T_l^{d}} \cdot \mathbf{H_l^{d-}}, \tag{S22}$$

where $\mathbf{T_c}$ and $\mathbf{T_l^{u(d)}}$ are diagonal matrices with diagonal elements $\mathbf{T_c}(s,s) = e^{i\beta_{sc}h_c}$ and $\mathbf{T_l^{u(d)}}(s,s) = e^{i2\beta_{sl}h_l^{\mathrm{u(d)}}}$, h_c and $h_l^{\mathrm{u(d)}}$ are the height of channel and lth upper (lower) groove. Defining $T_G^{\mathrm{u(d)}} = \begin{bmatrix} T_1^{\mathrm{u(d)}} & \cdots & 0 \\ \vdots & \ddots & \vdots \\ 0 & \cdots & T_L^{\mathrm{u(d)}} \end{bmatrix}$, we can obtain six linear equations groups:

$$M_{11}^{C}(\mathbf{\Psi}^{\mathrm{u}+} + \mathbf{\Psi^{u-}}) = M_{21}^{C}(\mathbf{H_C^{+}} + \mathbf{T_c}\mathbf{H_C'^{-}}), \tag{S23}$$

$$M_{11}^{C}(\mathbf{\Psi^{d+}} + \mathbf{\Psi^{d-}}) = M_{21}^{C}(\mathbf{T_c}\mathbf{H_C^{+}} + \mathbf{H_C'^{-}}), \tag{S24}$$

$$M_{12}^{G\mathrm{u}}(\mathbf{\Psi} + \mathbf{\Psi^{u-}}) = M_{22}^{G\mathrm{u}}(I + T_G^{\mathrm{u}})\mathbf{H_G^{u+}}, \tag{S25}$$

$$M_{12}^{G\mathrm{d}}(\mathbf{\Psi^{d+}} + \mathbf{\Psi^{d-}}) = M_{22}^{G\mathrm{d}}(T_G^{\mathrm{d}} + I)\mathbf{H_G^{d-}}, \tag{S26}$$

$$N_1(\mathbf{\Psi^{u+}} - \mathbf{\Psi^{u-}}) = N_{21}^{C}(\mathbf{H_C^{+}} - T_c\mathbf{H_C'^{-}}) + N_{22}^{G\mathrm{u}}(I - T_G^{\mathrm{u}})\mathbf{H_G^{u+}}, \tag{S27}$$

$$N_1(\mathbf{\Psi^{d+}} - \mathbf{\Psi^{d-}}) = N_{21}^{C}(T_c\mathbf{H_C^{+}} - \mathbf{H_C'^{-}}) + N_{22}^{G\mathrm{d}}(T_G^{\mathrm{d}} - I)\mathbf{H_G^{d-}}. \tag{S28}$$

where I is a unity matrix. Writing the linear equations groups in matrix form:

$$\begin{bmatrix} -M_{11}^{C} & 0 & M_{21}^{C} & M_{21}^{C}T_c & 0 & 0 \\ 0 & -M_{11}^{C} & M_{21}^{C}T_c & M_{21}^{C} & 0 & 0 \\ -M_{12}^{G\mathrm{u}} & 0 & 0 & 0 & M_{22}^{G\mathrm{u}}(I + T_G^{\mathrm{u}}) & 0 \\ 0 & -M_{12}^{G\mathrm{d}} & 0 & 0 & 0 & M_{22}^{G\mathrm{d}}(T_G^{\mathrm{d}} + I) \\ N_1 & 0 & N_{21}^{C} & -N_{21}^{C}T_c & N_{22}^{G\mathrm{u}}(I - T_G^{\mathrm{u}}) & 0 \\ 0 & N_1 & -N_{21}^{C}T_c & N_{21}^{C} & 0 & N_{22}^{G\mathrm{d}}(T_G^{\mathrm{d}} - I) \end{bmatrix} \begin{bmatrix} \mathbf{\Psi^{u-}} \\ \mathbf{\Psi^{d+}} \\ \mathbf{H_C^{+}} \\ \mathbf{H_G^{u+}} \\ \mathbf{H_C'^{-}} \\ \mathbf{H_G^{d-}} \end{bmatrix} = \begin{bmatrix} M_{11}^{C}\mathbf{\Psi^{u+}} \\ M_{11}^{C}\mathbf{\Psi^{d-}} \\ M_{12}^{G\mathrm{u}}\mathbf{\Psi^{u+}} \\ M_{12}^{G\mathrm{d}}\mathbf{\Psi^{d-}} \\ N_1\mathbf{\Psi^{u+}} \\ N_1\mathbf{\Psi^{d-}} \end{bmatrix} \tag{S29}$$

By solving Eq.S29 with incidence wave $\mathbf{\Psi^{u+}} = [0, \cdots, 1, \cdots, 0]^T$ and $\mathbf{\Psi^{d-}} = [0, \cdots, 0]^T$, the reflected and transmitted field can be decomposed with multiple modes governed by the complex coefficients $r_m^{\mathrm{u(d)}}$ and $t_m^{\mathrm{u(d)}}$. In this work, the plane-wave harmonic modes up to 20 and waveguide modes inside channel and grooves up to 30 are considered for precise. These evanescent modes can also influence the wave field near the metagrating.

Applying CMT on the four-port metagrating in main text, we can obtain the S-matrix by sweeping the k_x^i covering port 1 and port 2. Owing to the normalization relationship, the coefficients for each port in the scattering matrix must be derived from t_m and r_m through further processing. For the port 1, $k_x^i \in [0, G]$, $t^{\mathrm{u1(d1)}} = \sqrt{\frac{k_z^m}{k_z^0}} t_m^{\mathrm{u(d)}}$, $r^{\mathrm{u1(d1)}} = \sqrt{\frac{k_z^m}{k_z^0}} r_m^{\mathrm{u(d)}}$ ($m = -1$ for extraordinary scattering coefficients $t_e^{\mathrm{u1(d1)}}$ and $r_e^{\mathrm{u1(d1)}}$, $m = 0$ for ordinary scattering coefficients $t_o^{\mathrm{u1(d1)}}$ and $r_o^{\mathrm{u1(d1)}}$). For the port 2, $k_x^i \in [-G, 0]$, $t^{\mathrm{u2(d2)}} = \sqrt{\frac{k_z^m}{k_z^0}} t_m^{\mathrm{u(d)}}$, $r^{\mathrm{u2(d2)}} = \sqrt{\frac{k_z^m}{k_z^0}} r_m^{\mathrm{u(d)}}$ ($m = +1$ for $t_e^{\mathrm{u2(d2)}}$ and $r_e^{\mathrm{u2(d2)}}$, $m = 0$ for $t_o^{\mathrm{u2(d2)}}$ and $r_o^{\mathrm{u2(d2)}}$). Hence the eigenvalues of $\mathbf{S_1}$ and $\mathbf{S_2}$ in main text could be derived and the theoretical eigenvalue surfaces and winding around EP_1 and EP_2 can be obtained, shown in Supplementary Fig. 3a-d.

Supplementary Table 1: Parameters of metagrating sample

D (cm)	h_c (cm)	h_1 (cm)	h_2 (cm)	w_c (cm)	w_1 (cm)	w_2 (cm)	d_1 (cm)	d_2 (cm)	d_3 (cm)
33.34	2.90	0.35	0.96	0.46	0.73	1.52	0.20	0.20	0.22

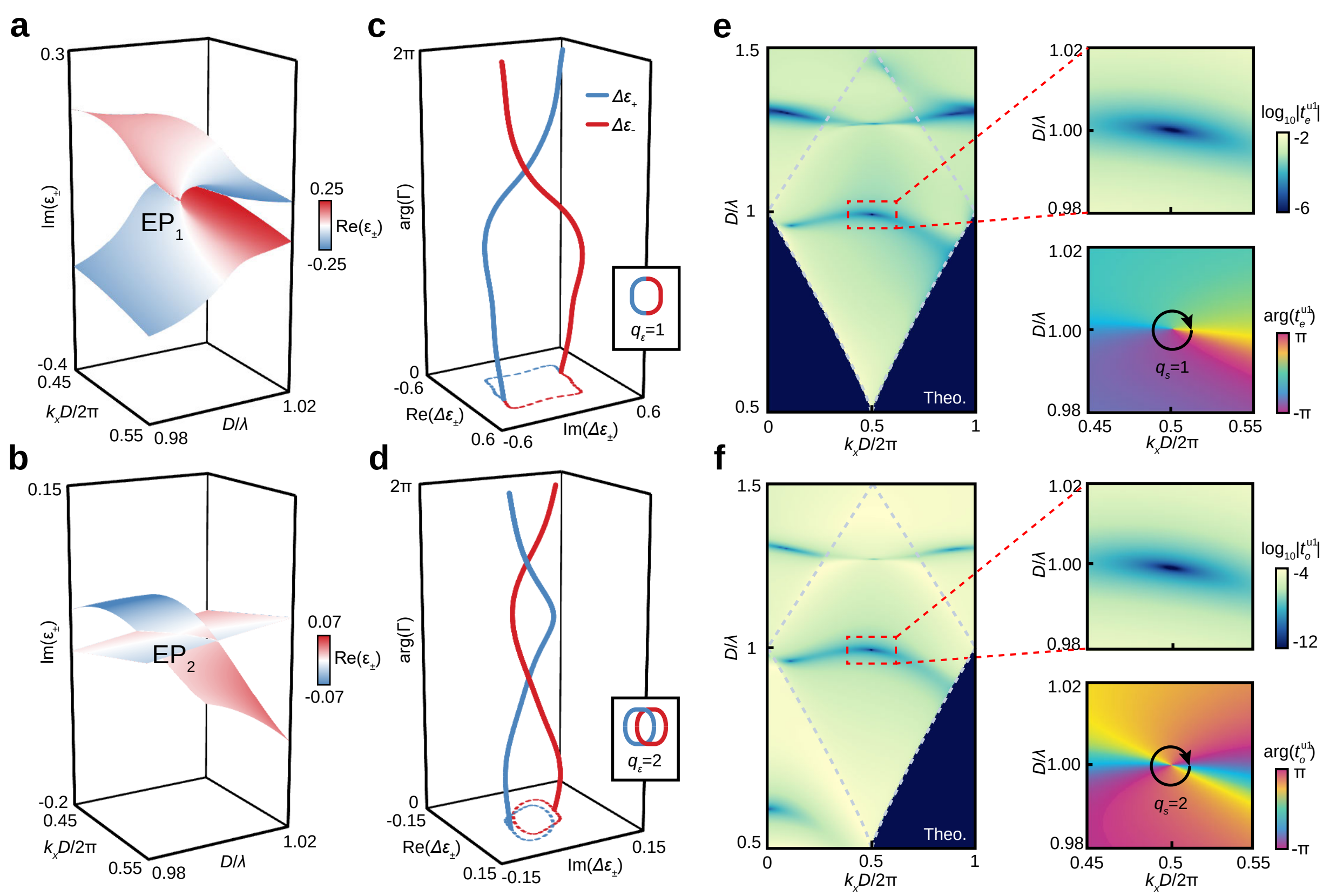

Supplementary Figure 3. a-d, Theoretical eigenvalue Riemann surfaces and winding around EP_1 and EP_2. **e-f**, Theoretical amplitude and phase map of the extraordinary coefficient t_e^{u1} and ordinary coefficient t_o^{u1}. The theoretical results show consistency with the simulated results in Fig. 2.

3 Supplementary Note 3. Constructing rule of projected EP

For the designed metagrating system shown in the main text, the element-wise absolute value of its full scattering matrix takes the form $\mathrm{abs}(\mathbf{S_p}) = \begin{bmatrix} 1 & 0 & 0 & 0 \\ 0 & 0 & 0 & 1 \\ 0 & 0 & 1 & 0 \\ 0 & 1 & 0 & 0 \end{bmatrix}$. Here we divide the full space with six 2-dimensional subspaces by the intrinsic symmetry of the metagrating, shown by Supplementary Fig. 4a. To be specific, we divide the full 4-dimentional space into paired subspaces, including the diagonal and anti-diagonal half-spaces defined by $\mathrm{C_2}$ rotation symmetry as port 1 and port 2, the upper and lower half-spaces defined by up-down mirror symmetry as port u and port d, and the left and right half-spaces defined by left-right mirror symmetry as port L and port R. In principle, by selecting 2 out of the 4 input ports and 2 out of the 4 output ports, there are in total 36 possible 2×2 sub-matrices (i.e. 36 2-dimentional subspaces). However, due to the strict mathematical requirement for the emergence of an EP (i.e., forming a non-diagonalizable Jordan block), the number of subsystems capable of hosting a projected EP is far fewer than 36. Mathematically, a second-order Jordan block requires that only one of its off-diagonal elements vanishes. Given that the scattering matrix $\mathbf{S_p}$ of our designed metagrating contains only four non-zero scattering elements, there are only two pairs of sub-matrices that support the projected EPs: $\mathbf{S_1}$, $\mathbf{S_2}$, $\mathbf{S_3}$ and $\mathbf{S_4}$. Among these, $\mathbf{S_1}$ and $\mathbf{S_2}$ have already been discussed in the main text. Below, we will detail how they are obtained through projection operations, and then we will introduce the other two EP-supporting subspaces, $\mathbf{S_3}$ and $\mathbf{S_4}$.

First, we introduce the corresponding input and output projection operators to show the projection process, using $\mathbf{S_1}$ and $\mathbf{S_2}$ as examples. Since the projected subspaces involve asym-

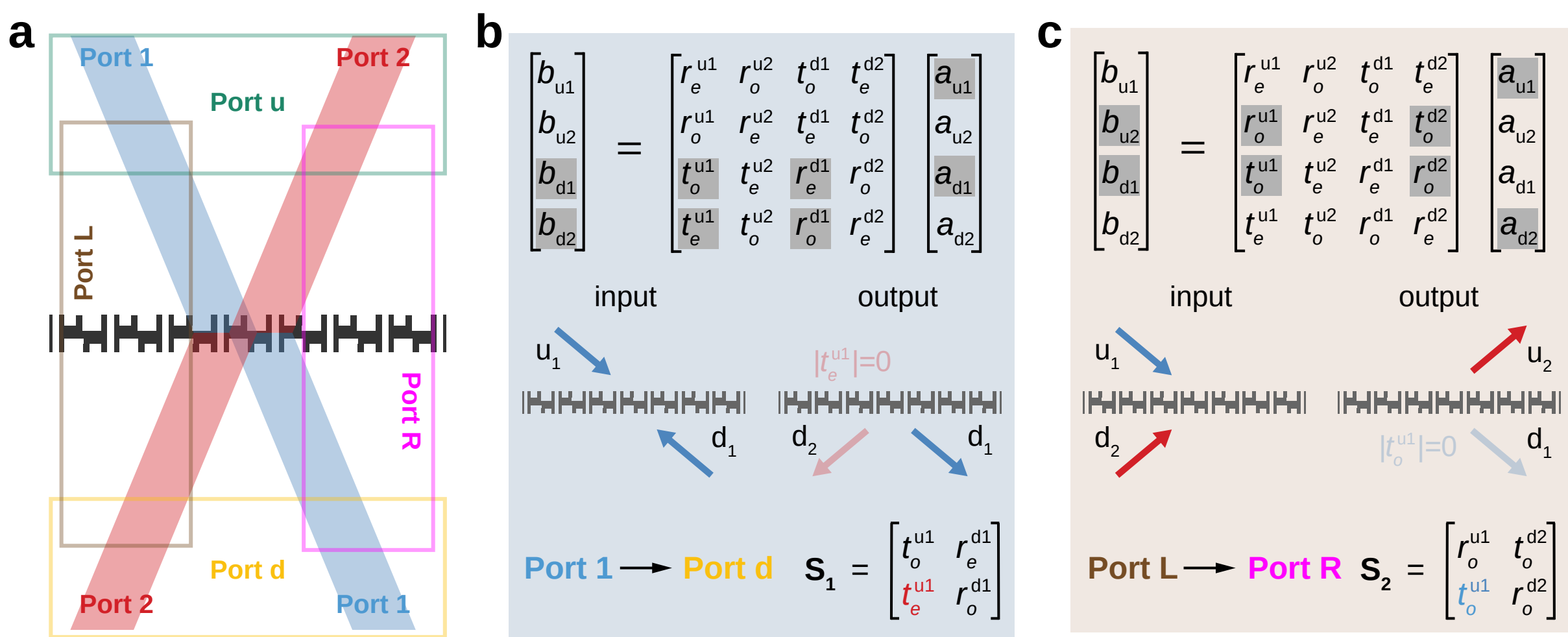

Supplementary Figure 4. **a**, The full space is divided into six 2-dimensional subspaces by the intrinsic symmetry of the metagrating. Specifically, ports u and d (vertical half spaces, colored by green and yellow), ports 1 and 2 (diagonal half-spaces, colored by blue and red), ports L and R (horizontal half spaces, colored by brown and magenta). **b** and **c**, Detailed projection process and input-output behaviors of $\mathbf{S_1}$ and $\mathbf{S_2}$ mentioned in the main text.

metric input-output channel combinations, they are defined by the asymmetric-projection relation as $\mathbf{S_n} = \mathbf{P_n^{out}} \mathbf{S_p} (\mathbf{P_n^{in}})^{\mathrm{T}}$ $(\mathrm{n} = 1, 2, 3, 4$, indicates the subsystem index), where $\mathbf{P_n^{in}}$ projects the excitation channels and $\mathbf{P_n^{out}}$ "selects" the corresponding scattering channels. For $\mathbf{S_1}$ and $\mathbf{S_2}$, $\mathbf{P_1^{in}} = \begin{bmatrix} 1 & 0 & 0 & 0 \\ 0 & 0 & 1 & 0 \end{bmatrix}$, $\mathbf{P_1^{out}} = \begin{bmatrix} 0 & 0 & 1 & 0 \\ 0 & 0 & 0 & 1 \end{bmatrix}$ and $\mathbf{P_2^{in}} = \begin{bmatrix} 1 & 0 & 0 & 0 \\ 0 & 0 & 0 & 1 \end{bmatrix}$, $\mathbf{P_2^{out}} = \begin{bmatrix} 0 & 1 & 0 & 0 \\ 0 & 0 & 1 & 0 \end{bmatrix}$. These asymmetric projection operators provide a clear delineation between the excitation channels and the scattering channels. Physically, $\mathbf{S_1}$ ($\mathbf{S_2}$) represents the input-output behaviors from port 1 (L) to port d (R), and has been discussed in main text. Supplementary Fig. 4b and 4c show the detailed projection process and input-output behaviors in subspaces of $\mathbf{S_1}$ and $\mathbf{S_2}$.

Then, we investigate the projected EPs of $\mathbf{S_3}$ and $\mathbf{S_4}$. $\mathbf{S_3}$ reveals the inter-port scattering behaviors from port L to port 2 (shown in Supplementary Fig. 5a): $\mathbf{b_3} = \mathbf{S_3 a_3}$, where $\mathbf{b_3} =$

$(b_{\mathrm{u}2}, b_{\mathrm{d}2})^{\mathrm{T}}$, $\mathbf{a_3} = (a_{\mathrm{u}1}, a_{\mathrm{d}2})^{\mathrm{T}}$ and $\mathbf{S_3} = \begin{bmatrix} r_o^{\mathrm{u}1} & t_o^{\mathrm{d}2} \\ t_e^{\mathrm{u}1} & r_e^{\mathrm{d}2} \end{bmatrix}$. Similarly, $\mathbf{S_4}$ defines the scattering behaviors from port u to port d (shown in Supplementary Fig. 5b): $\mathbf{b_4} = \mathbf{S_4}\mathbf{a_4}$, where $\mathbf{b_4} = (b_{\mathrm{d}2}, b_{\mathrm{d}1})^{\mathrm{T}}$, $\mathbf{a_4} = (a_{\mathrm{u}1}, a_{\mathrm{u}2})^{\mathrm{T}}$ and $\mathbf{S_4} = \begin{bmatrix} t_e^{\mathrm{u}1} & t_o^{\mathrm{u}2} \\ t_o^{\mathrm{u}1} & t_e^{\mathrm{u}2} \end{bmatrix}$.

Supplementary Figures 5c-d illustrate the simulated eigenvalue Riemann surfaces of $\mathbf{S_3}$ and $\mathbf{S_4}$ around the operated point in momentum-frequency domain. The EPs of $\mathbf{S_1}$ (main text, Fig. 2d) and $\mathbf{S_3}$ (Supplementary Fig. 5c) share the identical branch-cut topologies; both are charge-1 EPs. Likewise, the EPs encoded of $\mathbf{S_2}$ ((main text, Fig. 2e) and $\mathbf{S_4}$ (Supplementary Fig. 5d) share the identical Dirac-like intersecting topologies; both are charge-2 EPs. Notwithstanding that the two Riemann surfaces of the EPs from $\mathbf{S_1}$ and $\mathbf{S_3}$ are not exactly the same due to differences in their entries, they share the same topological nature; and the same holds for $\mathbf{S_2}$ and $\mathbf{S_4}$. Encircling the charge-1 EP and charge-2 EP yield the adiabatic evolution of eigenvalue differences for $\mathbf{S_3}$ and $\mathbf{S_4}$ as shown in Supplementary Figs. 5e and 5f, consistent with the results of Fig. 2f and 2h in main text. These comparisons between $\mathbf{S_1}$ ($\mathbf{S_2}$) and $\mathbf{S_3}$ ($\mathbf{S_4}$) demonstrate that the topological invariants of these projected EPs are determined by the vanishing element in their sub-matrices and remain robust regardless of the choice of other elements, which will be proved in Supplementary Note 4. Noted that due to the preserved C_2 symmetry, the conclusions obtained by the subsystem with $t_e^{\mathrm{u}1}$ ($t_o^{\mathrm{u}1}$) as vanishing element are identical to those for the subsystem $t_e^{\mathrm{d}1}$ ($t_o^{\mathrm{d}1}$) as vanishing element. In addition, by appropriately selecting the projected subsystems, it is also possible to generate other degeneracies besides EPs, such as diabolic points (DPs).

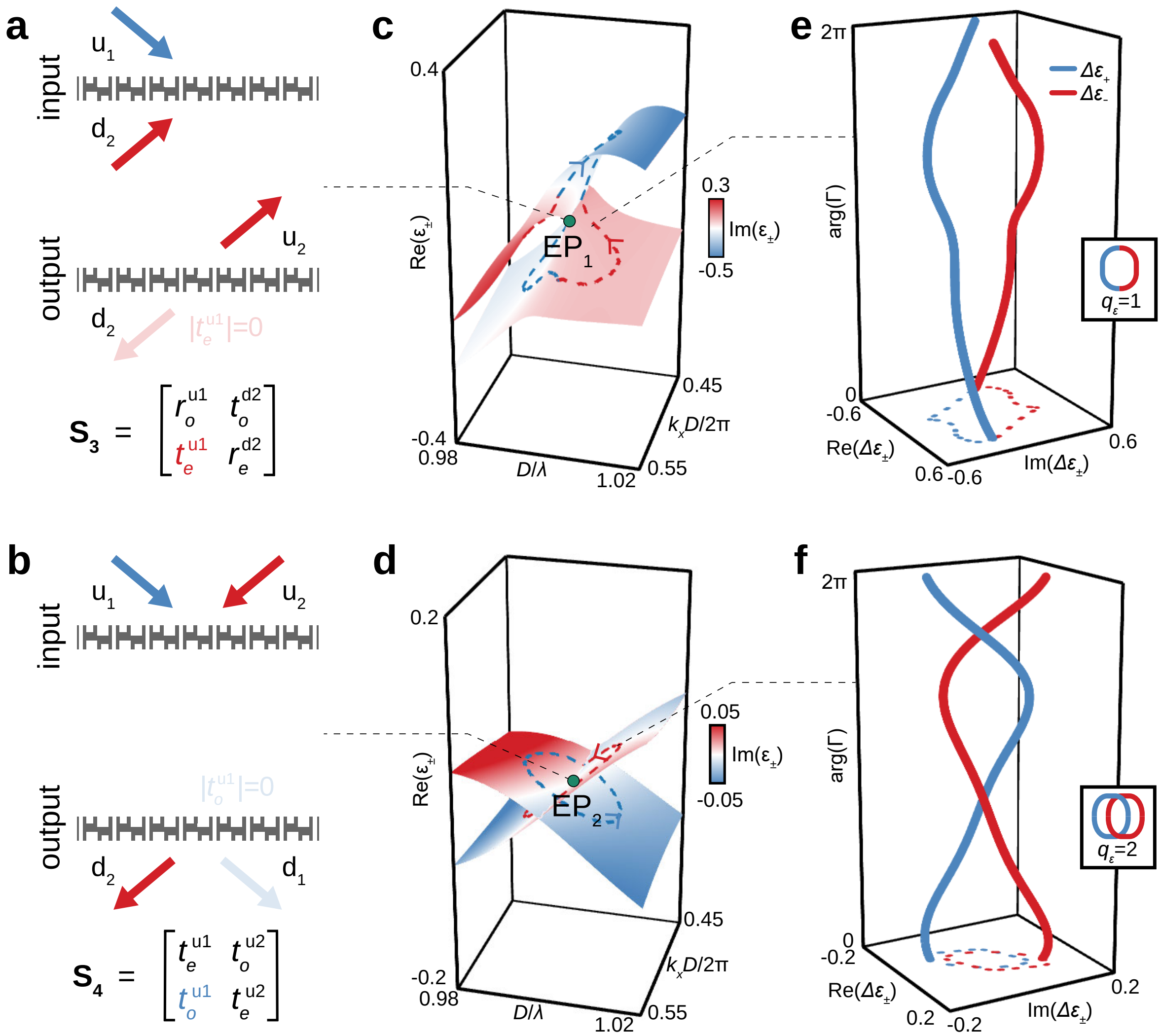

Supplementary Figure 5. **a** and **b**, Inter-port scattering behaviors of the two subsystems described by the sub-matrices $\mathbf{S_3}$ and $\mathbf{S_4}$. **c** and **d**, Eigenvalue Riemann surfaces of $\mathbf{S_3}$ and $\mathbf{S_4}$, which exhibit the topological structures of EP_1 (**c**) and EP_2 (**d**), similar with the results of $\mathbf{S_1}$ and $\mathbf{S_2}$ shown in Fig. 2(d-e). **e**-**f**, Adiabatic eigenvalue evolution around EP_1 (**e**) for $\mathbf{S_3}$ and EP_2 (**f**) for $\mathbf{S_4}$, confirming the two-band topological charge $q_\epsilon = 1$ and 2, consistent with the results in Fig. 2f and 2h.

4 Supplementary Note 4. Derivation about EP-STV correspondence

Topological charge of EP To reach the EP, the S-matrix adopts the Jordan-block form: $(h, \kappa; 0, h)$, the entries in which are transmission or reflection coefficients of associated ports. At the vicinity of EP, the S-matrix $\mathbf{S}(k_x, \omega) = [h, \kappa; \Delta\kappa, h + \Delta h]$. The eigenvalues of $\mathbf{S}$ can be derived as $\epsilon_\pm = h + \frac{\Delta h}{2} \pm \sqrt{\frac{\Delta h^2}{4} + \kappa\Delta\kappa}$. By the adiabatic eigenvalue evolution encircling EP, a two-band topological winding charge [3,4] can be defined as

$$q_\epsilon = v_\pm + v_\mp, \tag{S30}$$

where $v_{\pm(\mp)} = \frac{1}{2\pi}\oint_\Gamma \nabla_{\mathbf{m}}[\arg(\Delta\epsilon_{+(-)})] \cdot \mathrm{d}\mathbf{m}$ is the single-band topological charge, $\Delta\epsilon_{+(-)} = \epsilon_{+(-)} - \epsilon_{-(+)}$ denotes complex eigenvalues differences, $\mathbf{m}$ is an arbitrary 2D vector at k_x-ω plane. Γ represents the loop of encircling process. Substituting the elements of S-matrix and neglecting the impact of Δh and $\Delta\kappa$ (the higher-order term $\Delta h^2 \ll \kappa\Delta\kappa$ under a weakly perturbation approximation, $\Delta\kappa \ll \kappa$ and κ has no phase variation), $\arg(\Delta\epsilon_{+(-)}) = \arg(\sqrt{\Delta\kappa})$. Consequently, $q_\epsilon = q_s = \frac{1}{2\pi}\oint_\Gamma \nabla_{\mathbf{m}}[\arg(\Delta\kappa)] \cdot \mathrm{d}\mathbf{m}$, representing the phase winding number of vanishing term in S-matrix. To discriminate the polarity of topological charge, we define the phase accumulation along clockwise direction as positive.

Charge mapping from momentum space to real space For convenience, we also take the EP_1 of $\mathbf{S_1}$ as the example. Firstly, we decompose the output pulse pressure $P_{\mathrm{d2}}^{\mathrm{out}}$ governed by singular spectrum t_e^{u1} into the integral of a series of plane waves by Fourier transform:

$$P_{\mathrm{d2}}^{\mathrm{out}}(x, z, t) = \iint \widetilde{b}_{\mathrm{d2}}(k_x, k_z) e^{ik_x x + ik_z z - i\omega(k_x, k_z)t} \mathrm{d}k_x \mathrm{d}k_z, \tag{S31}$$

where $\widetilde{b}_{\mathrm{d2}}$ is the envelope spectrum of output pulse, being associated with the input pulse envelope spectrum by $\widetilde{b}_{\mathrm{d2}} = t_e^{\mathrm{u1}} \widetilde{a}_{\mathrm{u1}}$. k_x and k_z, as x- and z- component wave vectors, expand the momentum

space for each port. Considering the finite integral region $[k_{x0}-\delta k_x, k_{x0}+\delta k_x]$, $[k_{z0}-\delta k_z, k_{z0}+\delta k_z]$ and narrow band approximation ($\delta k_x \ll k_{x0}$ and $\delta k_z \ll k_{z0}$), the integral center of $\widetilde{b}_{\mathrm{d2}}$ is (k_{x0}, k_{z0}), $\omega(k_x, k_z)$ could be written as the form of first-order linear expansion near (k_{x0}, k_{z0}): $\omega(k_x, k_z) = \omega_0 + c\frac{k_{x0}}{k_0}K_x + c\frac{k_{z0}}{k_0}K_z$, where $K_x = k_x - k_{x0}$ and $K_z = k_z - k_{z0}$ are offsets versus (k_{x0}, k_{z0}) in momentum domain, $\omega_0 = ck_0$ is the center angular frequency. Then $P_{\mathrm{d2}}^{\mathrm{out}}(x, z, t)$ could be expressed as:

$$P_{\mathrm{d2}}^{\mathrm{out}}(x, z, t) = b_{\mathrm{d2}}(x, z, t)e^{ik_{x0}x+ik_{z0}z-i\omega_0 t}, \tag{S32}$$

where $b_{\mathrm{d2}}(x, z, t) = \int_{-\delta k_x}^{\delta k_x}\int_{-\delta k_z}^{\delta k_z} t_e^{\mathrm{u1}}(K_x, K_z)\widetilde{b}_{\mathrm{u1}}(K_x, K_z)e^{iK_x(x-\frac{k_{x0}}{k_0}ct)+iK_z(z-\frac{k_{z0}}{k_0}ct)}\mathrm{d}K_x\mathrm{d}K_z$ is the output pulse envelope in real space. Obviously, $b_{\mathrm{d2}}(x, z, t)$ propagates gradually in (x, z) domain as t varies. The center of wave packet is at $(\frac{k_{x0}}{k_0}ct, \frac{k_{z0}}{k_0}ct)$.

Moreover, to exhibit the propagation of the output pulse $P_{\mathrm{d2}}^{\mathrm{out}}$ in space-time domain (x, t), we transform the integration domain from (k_x, k_z) to (k_x, ω). Similarly, considering the first-order linear expansion near (k_{x0}, ω_0): $k_z(k_x, \omega) = k_{z0} - \frac{k_{x0}}{k_{z0}}K_x + \frac{k_0}{k_{z0}}\Omega$. $\Omega = \omega - \omega_0$ is the offset versus ω_0. Similar to Eq. S31, we have $b_{\mathrm{d2}}(x, z, t) = \int_{-\delta k_x}^{\delta k_x}\int_{-\delta\omega}^{\delta\omega} t_e^{\mathrm{u1}}(K_x, \Omega)\widetilde{a}_{\mathrm{u1}}(K_x, \Omega)e^{iK_x(x-\frac{k_{x0}}{k_{z0}}z)-i\Omega(t-\frac{k_0}{ck_{z0}}z)}\mathrm{d}K_x\mathrm{d}\Omega$, where $\delta\omega$ represent the integral limit in ω-dimension. With the variation of z, the output wave packet propagates in (x, t) domain, the center of which is at $(\frac{k_{x0}}{k_{z0}}z, \frac{k_0}{ck_{z0}}z)$. Adopting the accompanying transverse momentum $k_\perp$, we obtain

$$P_{\mathrm{d2}}^{\mathrm{out}}(r_\perp, \zeta) = b_{\mathrm{d2}}(r_\perp, \zeta)e^{i\omega_0\zeta} = e^{i\omega_0\zeta}\int_{-\delta k_\perp}^{\delta k_\perp}\int_{-\delta\omega}^{\delta\omega} t_e^{\mathrm{u1}}(k_\perp, \Omega)\widetilde{a}_{\mathrm{u1}}(k_\perp, \Omega)e^{ik_\perp r_\perp + i\Omega\zeta}\mathrm{d}k_\perp\mathrm{d}\Omega, \tag{S33}$$

where $(r_\perp, \zeta)$ is the co-moving coordinate. In polar coordinate, $b_{\mathrm{d2}}(r_\perp, \theta)$ could be expressed as

$$b_{\mathrm{d2}}(r, \theta) \approx \int_0^{\delta\rho}\int_0^{2\pi} t_e^{\mathrm{u1}}(\rho, \phi)\widetilde{a}_{\mathrm{u1}}(\rho, \phi)e^{i\rho r\cos(\phi-\theta)}\rho\mathrm{d}\rho\mathrm{d}\phi, \tag{S34}$$

where $r = \sqrt{r_\perp^2 + \zeta^2}$, $\theta = \tan^{-1}(\zeta/r_\perp)$, $\rho = \sqrt{(k_\perp)^2 + (\frac{\Omega}{c})^2}$, $\phi = \tan^{-1}(\Omega/ck_\perp)$. The integral limit $\delta\rho = \sqrt{(\delta k_\perp)^2 + (\frac{\delta\omega}{c})^2}$. According to the discussion of main text, we suppose that

$t_e^{\mathrm{u1}}(\rho,\phi) = f(\rho)[A\exp(iq_s\phi) + B\exp(-iq_s\phi)]$. The vortex ellipticity $\eta_s = \frac{A-B}{A+B}$ represents the anisotropy of the phase distribution. The topological charge of t_e^{u1} is q_s if $\eta_s > 0$, and $-q_s$ if $\eta_s < 0$. when $\eta_s = \pm 1$, the phase of the vortex is an isotropic circular distribution. Noted that we use $\eta_s = 1$ ($B = 0$, $t_e^{\mathrm{u1}}(\rho,\phi) = f(\rho)e^{iq_s\phi}$) as the premise for the derivation in the main text. With the Gaussian profile, the incident pulse has the form $\widetilde{a}_{\mathrm{u1}}(k_\perp,\Omega) = \exp[-\frac{k_\perp^2}{\Delta k_\perp{}^2} - \frac{\Omega^2}{\Delta\omega^2}]$, where $\Delta k_\perp$ and $\Delta\omega$ determine the ellipticity of input pulse envelope. Considering $\Delta\rho = \frac{\Delta\omega}{c} = \Delta k_\perp$, the polar coordinate form is $\widetilde{a}_{\mathrm{u1}}(\rho) = \exp(-\frac{\rho^2}{\Delta\rho^2})$, which lacks phase variation. Hence the output (transmitted) pulse envelope could be described as

$$
\begin{aligned}
b_{\mathrm{d2}}(r,\theta) &= \int_0^{\delta\rho} \widetilde{a}_{\mathrm{u1}}(\rho) f(\rho)\rho \int_0^{2\pi} [Ae^{iq_s\phi} + Be^{-iq_s\phi}] \cdot e^{i\rho r\cos(\phi-\theta)} \mathrm{d}\rho\mathrm{d}\phi \\
&= \int_0^{\delta\rho} \widetilde{a}_{\mathrm{u1}}(\rho) f(\rho)\rho \int_0^{2\pi} [Ae^{iq_s\theta} \cdot e^{-q_s(-\phi+\theta)+\rho r\cos(\phi-\theta)} + Be^{-iq_s\theta} \\
&\cdot e^{-q_s(-\phi-\theta)+\rho r\cos(\phi-\theta)}]\mathrm{d}\phi,
\end{aligned} \tag{S35}
$$

setting $\gamma = -\phi + \theta + \frac{\pi}{2}$ and $\tau = \phi - \theta + \frac{\pi}{2}$, thus $\mathrm{d}\gamma = -\mathrm{d}\phi$ and $\mathrm{d}\tau = \mathrm{d}\phi$. Substituting γ and τ, the integral of $\mathrm{d}\phi$ term in Eq. S35 becomes

$$
\begin{aligned}
& Ae^{iq_s\theta} \int_0^{2\pi} e^{-iq_s(-\phi+\theta)+i\rho r\cos(\phi-\theta)}\mathrm{d}\phi + Be^{-iq_s\theta} \int_0^{2\pi} e^{-iq_s(\phi-\theta)+i\rho r\cos(\phi-\theta)}\mathrm{d}\phi \\
&= -Ae^{iq_s\theta} \int_{\theta+\frac{\pi}{2}}^{\theta-\frac{3\pi}{2}} e^{-iq_s(\gamma-\frac{\pi}{2})+i\rho r\cos(\frac{\pi}{2}-\gamma)}\mathrm{d}\gamma + Be^{-iq_s\theta} \int_{-\theta+\frac{\pi}{2}}^{-\theta+\frac{5\pi}{2}} e^{-iq_s(\tau-\frac{\pi}{2})+i\rho r\cos(\tau-\frac{\pi}{2})}\mathrm{d}\tau \\
&= -i^{q_s}Ae^{iq_s\theta} \int_0^{-2\pi} e^{i(-q_s\gamma+\rho r\sin\gamma)}\mathrm{d}\gamma + i^{q_s}Be^{-il_s\theta} \int_0^{2\pi} e^{i(-l_s\tau+\rho r\sin\tau)}\mathrm{d}\tau \\
&= 2\pi i^{q_s}[Ae^{iq_s\theta} + Be^{-iq_s\theta}]J_{q_s}(\rho r),
\end{aligned} \tag{S36}
$$

where $J_{q_s}(\rho r) = \frac{1}{2\pi}\int_0^{2\pi} e^{i(-q_s\gamma+\rho r\sin\gamma)}$ is the q_s-th order Bessel function of the first type. Consequently, the output pulse envelope become $b_{\mathrm{d2}}(r,\theta) = 2\pi i^{q_s}[Ae^{iq_s\theta} + Be^{-iq_s\theta}] \cdot \int_0^{\delta\rho} \widetilde{a}_{\mathrm{u1}}(\rho) f(\rho) J_{q_s}(\rho r)\rho\mathrm{d}\rho$. In other words, the output pulse is the STV carrying vortex charge of q_s in $r_\perp$-ζ domain, which is a mixed spatiotemporal domain.

In summary, the exceptional topology around the EP can induce the scattering singularity

with the identical charge ($q_\epsilon = q_s$) in momentum space, hence mapping the corresponding STV into real space. The EP-STV correspondence has been proved.

Robustness of charge mapping Although Eq. S36 provides an analytical solution under specific approximations (e.g., local phase-vortex of scattering coefficients, a circular incident pulse, limited integration region), the wave structure of the scattering STV is determined by the interplay between phase-vortex of the scattering coefficient and the morphology of incident pulse. We define the ellipticity of the incident Gaussian pulse spectrum as η_i, generalizing the circular integration region in Eq. S34 to an anisotropic ellipse. In order to examine the robustness of the charge mapping process, we calculate the numerically integrated pressure and phase distributions of scattering STVs for various relationships between η_i and the ellipticity of scattering spectral vortex η_s in two cases of $q_s = 1$ and $q_s = 2$, shown in Supplementary Fig. 6.

The results reveal that η_s and η_i exert two competing stretching effects into the phase distribution of STV and the scattering pulse envelope during the mapping from momentum space to real space, respectively. Moreover, the stretching induced by η_s arises from the mapping of anisotropic parameters A and B in Eq. S36, whereas the inverse-stretching induced by η_i stems from the Fourier transform from the momentum-frequency domain to the space-time domain. Crucially, regardless of these anisotropic distortions, the charge mapping process exhibits a topologically protected robustness. For $q_s = 1$, the topological charge of the scattering STV strictly corresponds precisely to that of the scattering spectral phase-vortex. For $q_s = 2$, while the singularity of the scattering STV is unstable (as discussed in the following subsection), the total topological charge remains conserved, further validating the robustness of the charge mapping.

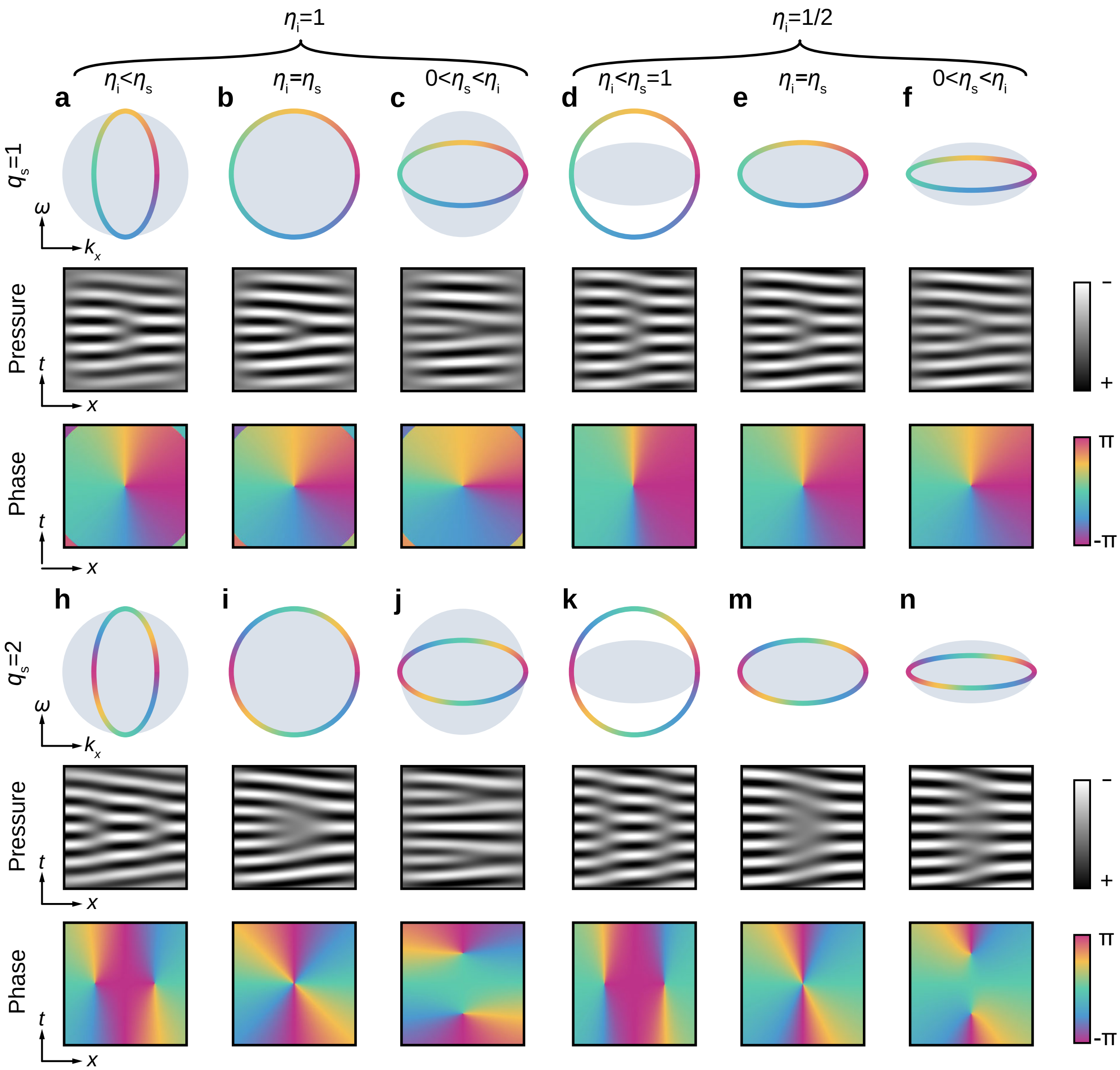

Supplementary Figure 6. The pressure and phase distribution of charge-1 ($q_s = 1$, **a-f**) and charge-2 ($q_s = 2$, **h-n**) scattering STV in space-time domain, under the different relationship between η_s (the ellipticity of the scattering coefficient vortex, denoted by the colored loop) and η_i (the ellipticity of incident pulse envelope spectrum, denoted by the gray region).

Disintegration of charge-2 STV While Eq. S36 guarantees a strict mapping of topological charges between scattering spectral phase-vortices and scattering STVs, it does not ensure the stability of higher-charge singularity. Specifically, a charge-2 STV ($q_s = 2$) is highly prone to split into two charge-1 elementary STVs, primarily due to the mismatch between the scattering spectrum (η_s) and incident pulse spectrum (η_i). This instability is investigated in Supplementary Fig. 6h-n, which display how the competing stretch leveraged by η_i and η_s affects the wave structure of charge-2 STV.

When $\eta_i = 1$, which means that the shape of incident pulse is a standard circle, we display the scattering patterns of the STV for the three cases where $\eta_i < \eta_s$ (Supplementary Fig. 6a), $\eta_i = \eta_s$ (Supplementary Fig. 6b) and $1 < \eta_s < \eta_i$ (Supplementary Fig. 6c). It is obvious that when $\eta_i < \eta_s$ or $0 < \eta_s < \eta_i$, the charge-2 STV splits into two charge-1 singularities along the space (x) or time (t) dimension. Only when $\eta_i = \eta_s$ does the charge-2 STV not split, retaining an individual charge-2 singularity. In Supplementary Fig. 6d-f, we set $\eta_i = 1/2$, under the conditions of $\eta_i < \eta_s = 1$ (Supplementary Fig. 6d), $\eta_i = \eta_s$ (Supplementary Fig. 6e) and $0 < \eta_s < \eta_i$ (Supplementary Fig. 6f), we obtain the same conclusions as Supplementary Fig. 6a-c. Consequently, the charge-2 STV remains stable only when the opposing stretching effects are perfectly matched, i.e., $\eta_i = \eta_s$. However, the perfect shapes of incident pulse and scattering spectrum vortex are virtually impossible in practical applications. It should be noted, that medium dispersion—which causes phase velocity inconsistencies among various plane-wave components—serves as an additional factor contributing to wave packet disintegration during propagation.

Theoretical prediction of STV generation According to Eq. S31, we could obtain the theoretical predicted output STV pulses - anomalous-transmitted pulse and specular-transmitted pulse by

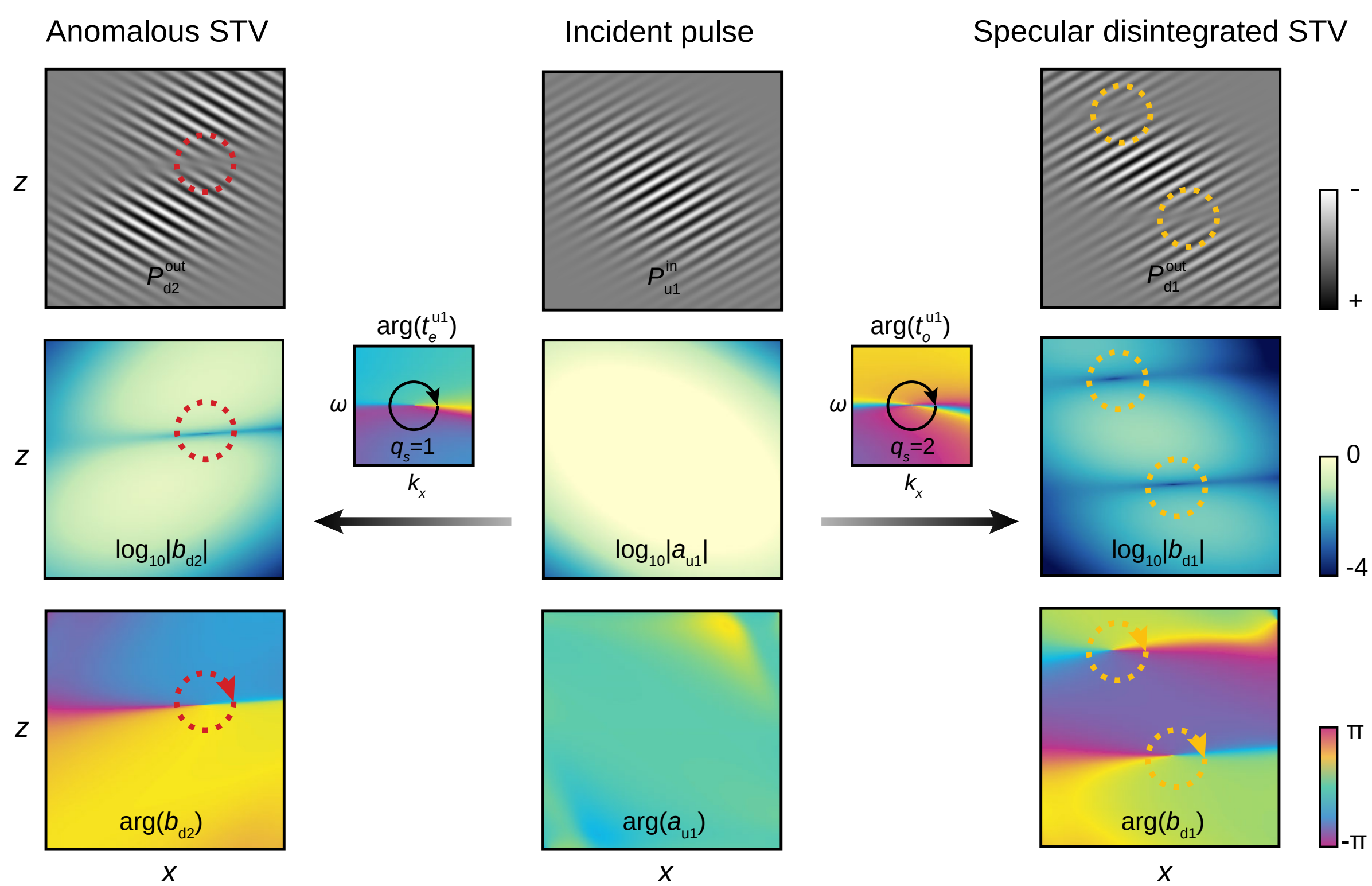

Supplementary Figure 7. The pressure field, envelope amplitude and phase of incident pulse ($P_{\mathrm{u1}}^{\mathrm{in}}$, $\log_{10}(|a_{\mathrm{u1}}|)$ and $\arg(a_{\mathrm{u1}})$ in middle panel), anomalous-transmitted pulse ($P_{\mathrm{d2}}^{\mathrm{out}}$, $\log_{10}(|b_{\mathrm{d2}}|)$ and $\arg(b_{\mathrm{d2}})$ in left panel) and specular-transmitted pulse ($P_{\mathrm{d1}}^{\mathrm{out}}$, $\log_{10}(|b_{\mathrm{d1}}|)$ and $\arg(b_{\mathrm{d1}})$ in left panel) obtained by Fourier transform to simulated 1st-order and 0th-order transmission coefficients (t_e^{u1} and t_o^{u1}) spectra, which exhibit the topological charges q_ϵ of EP_1 and EP_2

Fourier transforming to the simulated 1st-order and 0th-order transmission spectra (t_e^{u1} and t_o^{u1}), which are demonstrated in Supplementary Fig. 7. In Supplementary Fig. 7, the width of Gaussian incident pulse envelope spectrum, both Δk_x and Δk are $0.05G$ ($\Delta\omega = c\Delta k$), which means $\eta_i = 1$. It is found that the phase-vortex of t_e^{u1} and t_o^{u1} exhibits significant spectral distortions and are compressed in ω-axis ($0 < \eta_s < 1$). Based on the conclusions of previous section, since the incident pulse envelope does not match the anisotropic phase-vortex of t_e^{u1} and t_o^{u1} ($\eta_s < \eta_i$), the vortex-wave structure will be stretched in propagation direction. This is the reason of why the specular transmitted wave packet carries two split charge-1 STVs but with the total topological charge of 2 ($q_s = 2$), shown in the right panel in Supplementary Fig.7. Theoretical predictions in Supplementary Fig.7 are consistent with the time-resolved simulation results shown in Fig. 4. By coordinate transformation from (x, z, t) to $(r_\perp, \zeta)$, Supplementary Fig.7 can be exported as Fig. 3.

5 Supplementary Note 5. Details of simulation and experiment setups

Supplementary Figure 8 displays the detailed simulation setup. The setup of infinite-period model is shown in Supplementary Fig. 8a, to derive the decomposed reflection and transmission coefficients obtained by Fourier transforming to the signals in scan lines.

In order to visualizing the STV generation, the transformation from the generalized cartesian coordinate to the pulse-centered coordinate should be considered, shown in Supplementary Fig. 8b, that is, (k_x, k_z)→($k_\perp$, $k_\parallel$) and (x, z)→($r_\perp$, $r_\parallel$). $k_\perp$ and $k_\parallel$ ($r_\perp$ and $r_\parallel$) are the transverse and longitudinal wavevectors (space coordinates), which are perpendicular and parallel to propagation axis. The transformation could be defined as:

$$\begin{aligned} k_\perp &= k\sin[\sin^{-1}\left(\frac{k_x}{k}\right) - \sin^{-1}(\frac{k_{x0}}{k_0})], \\ r_\perp &= \sqrt{x^2+z^2}\sin[\tan^{-1}\left(\frac{x}{z}\right) - \sin^{-1}(\frac{k_{x0}}{k_0})]. \end{aligned} \tag{S37}$$

In time-domain simulation presented in main text, we use a finite-period structure consisting of 100 periods and define a rectangular area as solution domain with the upper boundary to generate incident pulse and other three boundaries as scattering boundaries, which is shown in Supplementary Fig. 8c. The form of Gaussian incident pulse at incident boundary is $\Phi(r_\perp, r_\parallel, t) = \Phi(r_\perp, r_\parallel)\cdot T(t)$, where

$$\Phi(r_\perp, r_\parallel) = \sqrt{\frac{w_0}{w(r_\parallel)}}\cdot e^{-\frac{r_\perp^2}{w(r_\parallel)^2}}\cdot e^{i(kr_\parallel + \tan^{-1}\frac{r_\parallel}{r_\parallel^R} + \frac{k_\perp^2}{2R(r_\parallel)})}, \tag{S38}$$

and

$$T(t) = e^{-(\alpha+i\beta)t^2}\cdot e^{-i\omega_0 t}. \tag{S39}$$

w_0 is the radius of beam waist, $w(r_\parallel) = w_0\sqrt{1+\frac{r_\parallel}{r_\parallel^R}}$, $r_\parallel^R = \frac{\pi w_0^2}{\lambda_0}$ denotes the Rayleigh range. $\omega_0 = 2\pi f_0$ and λ_0 are center angular frequency and wavelength of the pulse. $R(r_\parallel) = r_\parallel[1+(\frac{r_\parallel^R}{r_\parallel})^2]$ is the wavefront curvature radius, $\tan^{-1}(\frac{r_\parallel}{r_\parallel^R})$ is the Gouy phase. $\alpha = \frac{1}{\delta t^2}$, where δt is the time

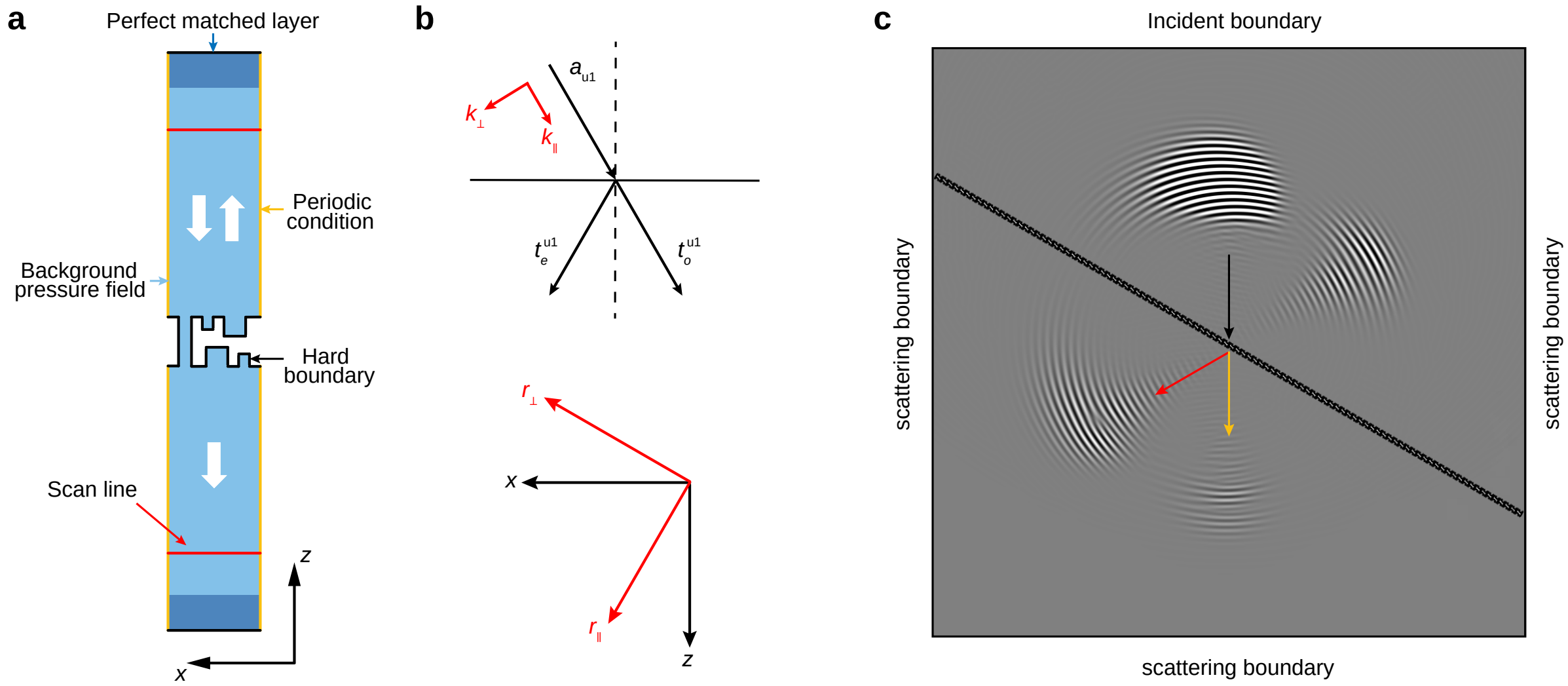

Supplementary Figure 8. Numerical simulation schematic. **a**, Setup of infinite-period model. **b**, the coordinates transformations. **c**, The setup of time-domain simulation of visualizing STV generation in finite-period model.

duration of pulse, β indicates the linear chirping factor. $|\alpha + i\beta|$ represents the bandwidth of pulse spectrum. For simulation of this work, w_0 is set as $2\lambda_0$, $\delta t = 0.4$ second, $\beta = 0$ with no chirping, the center frequency $f_0 = 7.03$ Hz.

Supplementary Figure 9 shows the detailed experimental platform and data processing from photo to signal field. By 2D Fourier transformation of signal field, we could obtain the center wavelength of the pulse, denoted in the inset of Supplementary Fig. 9d.

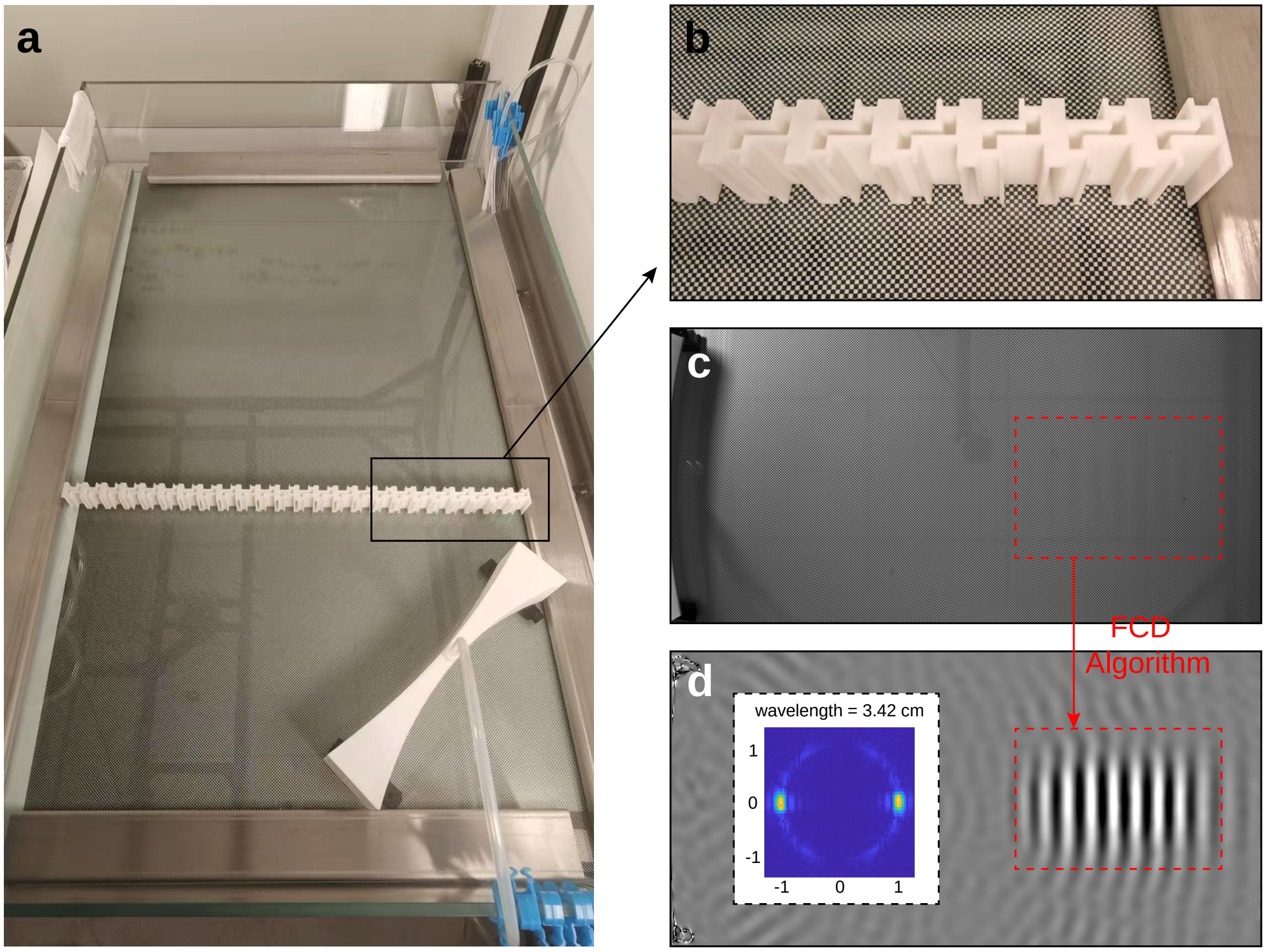

Supplementary Figure 9. **a**, Photo of experimental setup, the background area under the liquid tank is a black-and-white checkerboard pattern, which is required in Fast Checkerboard Demodulation (FCD) algorithm for obtaining the 2D field of the liquid surface wave. **b**, Details of meta-grating sample. **c-d**, Image processing for a pulse signal with FCD Algorithm [5], the inset indicates the 2D Fourier transformation of signal field, which could obtain the approximate center wavelength of the pulse.

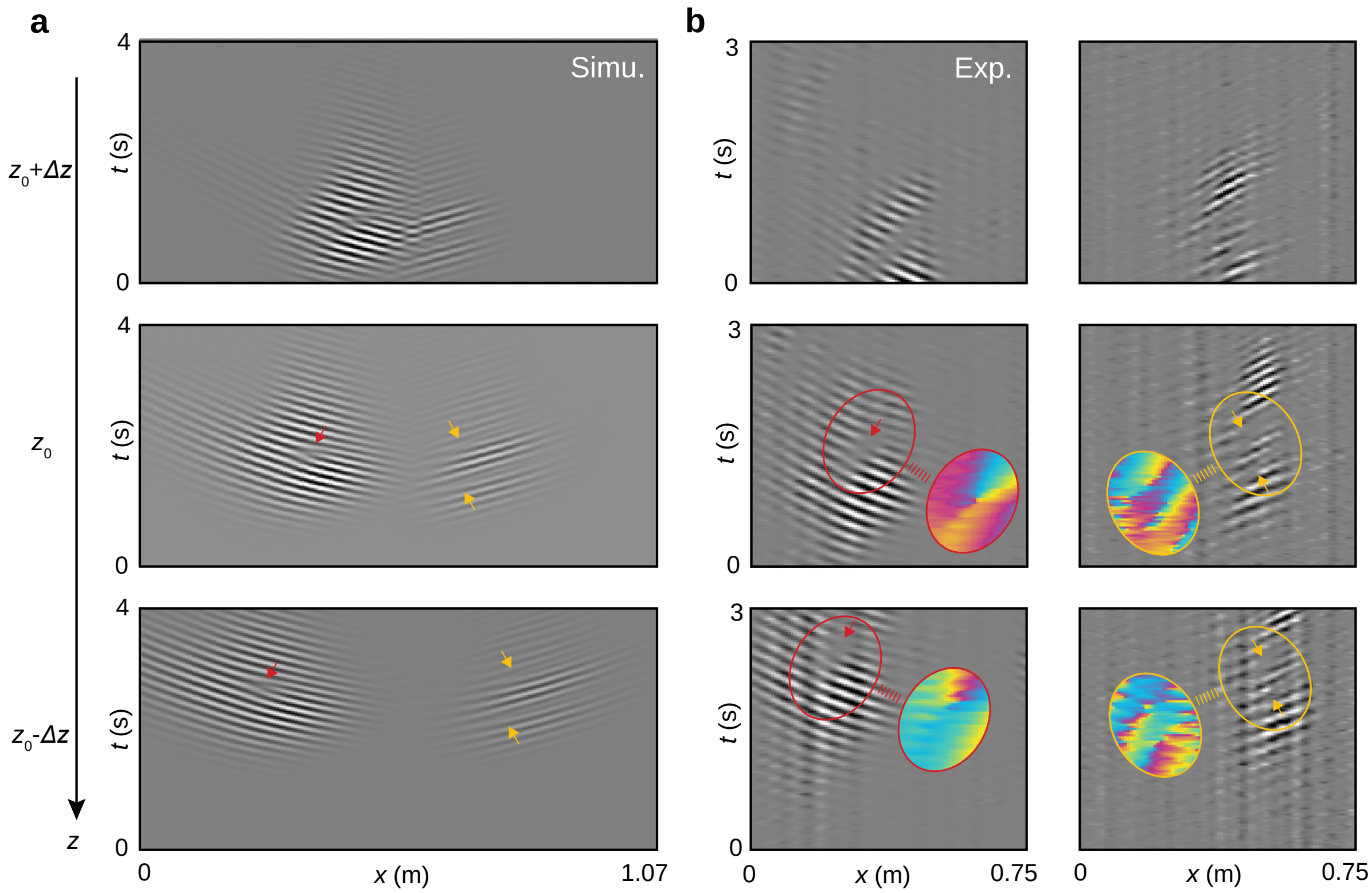

Supplementary Figure 10. Observation of charge-1 STV in anomalous-transmitted side (EP_1) and disintegrated charge-2 STV in specular-transmitted side (EP_2) in space-time domain, as a supplement of Fig. 4. **a**, simulated transmitted pressure field in space-time domain at three fixed scanning lines $z = z_0 - \Delta z_0, z_0, z_0 + \Delta z_0$. **b**, experimental results compared with **a**, the space-time envelope phases of transmitted STVs are shown in insets of the middle and lower panels.

6 Supplementary Note 6. Additional experimental results

Experimental observations of STVs in space-time domain As mentioned in main text, observing STVs could be classed into two strategies: 1. time-sequences field pattern $P(x, z)$ at fixed moments; 2. space-sequences field pattern $P(x, t)$ at fixed scanning lines. The result of the first strategy has been exhibited in Fig. 4 of main text, then in Supplementary Fig. 10 we exhibit the result of the second strategy. In Supplementary Fig. 10a, we choose three scanning lines $z = z_0 + \Delta z_0, z_0, z_0 - \Delta z_0$ ($\Delta z_0 = 5D$) parallel to the metagrating as spatial sequences, then

measure the pressure at scanning lines within the same time range of 4 seconds to exhibit transmitted field $P_{\mathrm{tran}}(x,t)$ in numerical simulation. Supplementary Fig. 10b shows the experimental field patterns compared with Supplementary Fig. 10a, but the time range and Δz_0 of which are set as 3 seconds and $4D$. The envelope phases of anomalous-transmitted charge-1 STV and specular-transmitted disintegrated charge-2 STVs are plotted in insets of the middle and lower panels, showing the agreement with the result in Fig. 4 and discussion in Supplementary Note 3. Notably the intensity of t_o^{u1} spectrum is weaker than t_e^{u1} spectrum, hence the STVs in specular-transmitted side is harder to observe than in anomalous-transmitted side. In conclusion, the transmitted field patterns in both spatial and space-time domain could mirror the topological properties of EP_1 and EP_2 into the real space.

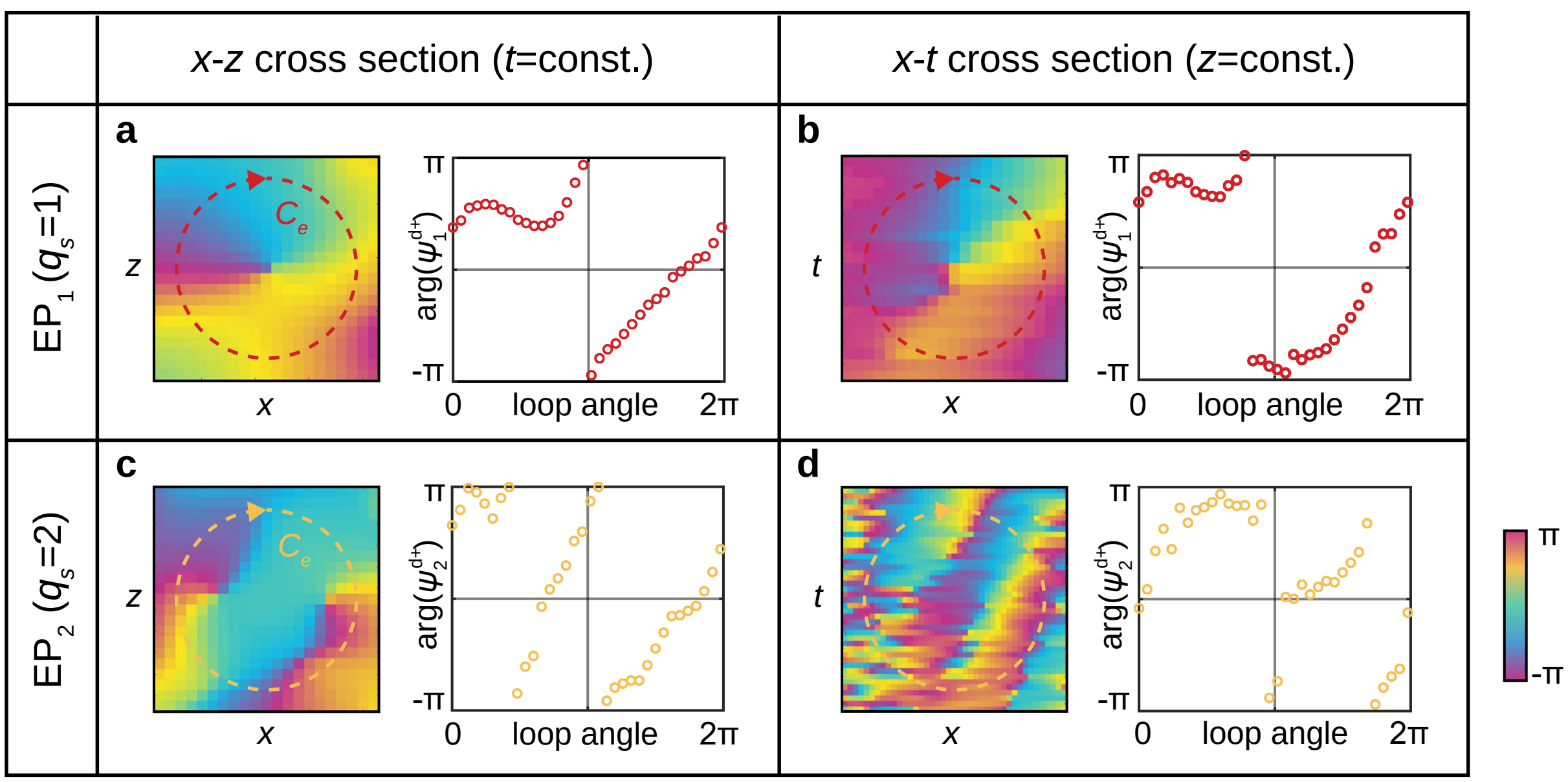

Supplementary Figure 11. Topological charge q^{exp} extracted from the transmission phase pattern in spatial (x-z, $t = \mathrm{const.}$) cross section (**a** and **c**) and space-time (x-t, $z = \mathrm{const.}$) cross section (**b** and **d**).

Topological charge of STVs extracted from experimental results In view of the experimental results, we can extract the topological charges of STVs (q^{exp}) from phase patterns of scattering pulse envelopes. For anomalous-transmitted pulse corresponding to EP_1, $q_1^{\text{exp}} = \frac{1}{2\pi}\oint_C d\arg(b_{\text{d2}})$, and for specular-transmitted pulse corresponding to EP_2, $q_2^{\text{exp}} = \frac{1}{2\pi}\oint_C d\arg(b_{\text{d1}})$, where C is a closed integral loop encircling the pulse center shown in Supplementary Fig. 11. In Supplementary Fig. 11, we exhibit the distribution of $\arg(b_{\text{d1}})$ and $\arg(b_{\text{d2}})$ in spatial section (x-z plane with t=const., the data from Fig. 4i) and space-time section (x-t plane with z=const., the data from Supplementary Fig. 10b), as well as the phase variation along the integral loop. It is evident that whether in x-z or x-t cross section, b_{d2} carries one charge-1 singularity and b_{d1} carries two charge-1 singularities. Considering one encircling cycle along C, the phase $\arg(b_{\text{d2}})$ undergoes a 2π-change, while $\arg(b_{\text{d1}})$ undergoes a 4π-change, which indicates that the topological charge of b_{d2} is $q_1^{\text{exp}} = 1$ and the total topological charge of b_{d1} is $q_2^{\text{exp}} = 2$. The results from the experimental analysis are consistent with our theory and simulations, confirming the EP-STV correspondence in our work.

Experimental scattering behaviors of the total metagrating system The simulated and experimental scattering behaviors of the total four-port metagrating illustrated by Fig. 2 is shown in Supplementary Fig. 12. Supplementary Figs. 12a and 12b indicate the results of infinite-period and finite-period simulations. The simulated and experimental results are basically consistent. Note that the specular transmitted pressure under incidence of port u_2 is weak in experiment, that is due to the capillarity caused by the micro-scales of transmissive channel, increasing the viscous dissipation.

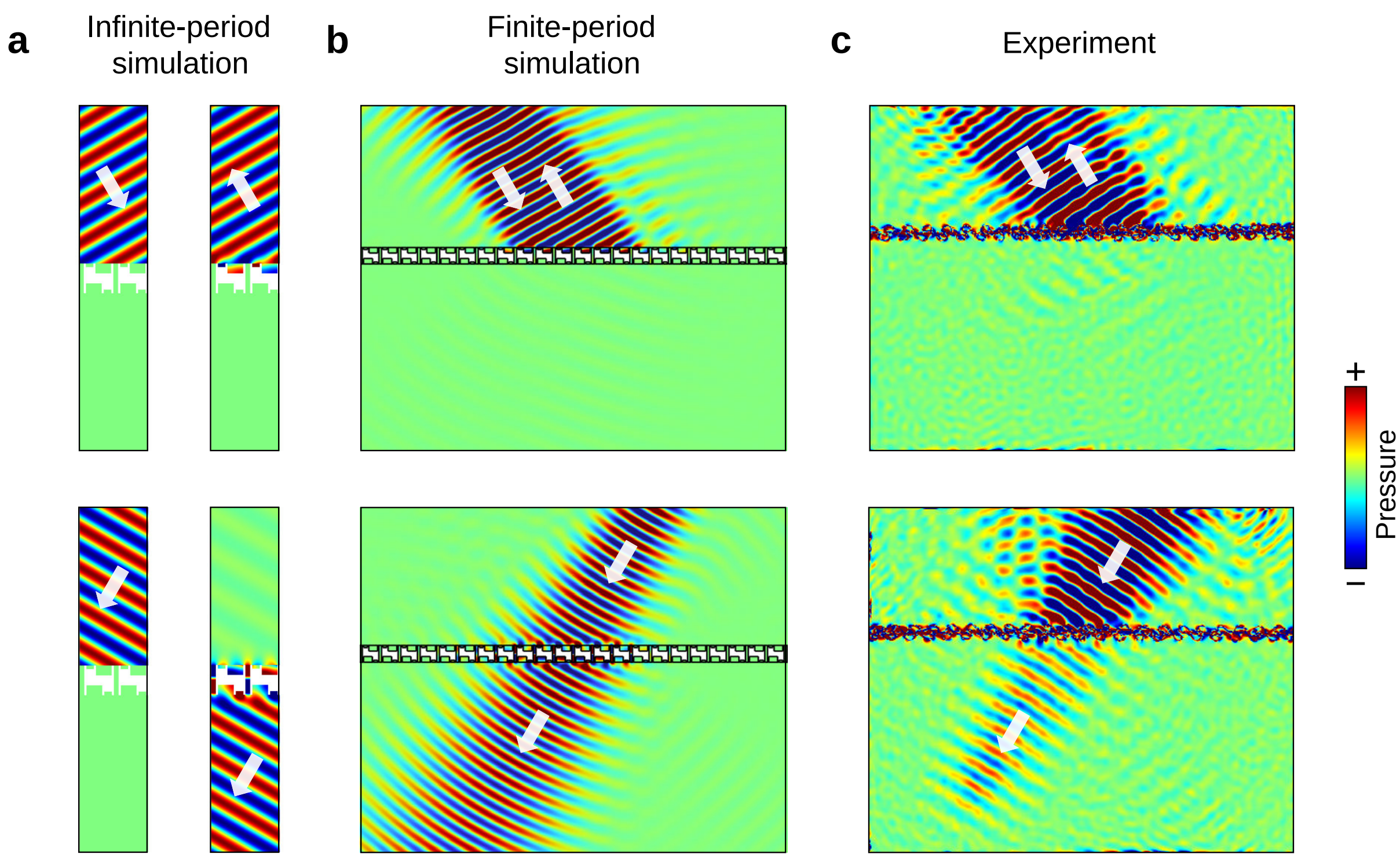

Supplementary Figure 12. Scattering behaviors of the total four-port system via infinite-period simulation (**a**), finite-period simulation (**b**) and experiment (**c**).

Supplementary Note 7. Evolution of projected EPs governed by C_2-preserving and C_2-breaking perturbations

In a lossless and passive four-port metagrating, the projected subsystems are inherently coupled due to the unitarity of scattering matrix. The structural symmetry tightly influences and modulates this inter-system coupling. The zigzag metagrating discussed in the main text breaks both left-right and up-down mirror symmetries while preserving C_2 symmetry—a feature crucial for the dynamic observation of projected EPs. Specifically, C_2 symmetry simplifies the total scattering matrix ($\mathbf{S_p}$) by mapping the scattering channels into two symmetric pairs (u_1 to d_1, u_2 to d_2). As derived in Supplementary Note 4, the exceptional topology of EP_1 and EP_2 is encoded in their respective vanishing matrix elements, t_e^{u1} and t_o^{u1}. These elements scatter waves from the same shared input channel (u_1) to entirely different output channels: t_e^{u1} routes to the bottom-left port (d_2), while t_o^{u1} routes to the bottom-right port (d_1). Therefore, this configuration significantly constrains the inter-system coupling between the projected subsystems $\mathbf{S_1}$ and $\mathbf{S_2}$. Consequently, the two induced projected EPs (EP_1 and EP_2) are firmly anchored at identical locations in the momentum-frequency domain. This spectral coincidence enables the simultaneous observation of STVs, governed by distinct exceptional topologies, along different spatial scattering channels under single-pulse excitation. When C_2 symmetry is broken, the coupling between subsystems becomes more complex, EP_2 split, destroying the location-anchoring with EP_1.

To elucidate this mechanism, we introduce a C_2 -preserving geometric perturbation Δw_1 to the original structure, as shown in Supplementary Fig. 13a. In Supplementary Fig. 13b, we illustrates the evolution of the spectral singularities (the phase vortex of vanishing matrix elements t_e^{u1} in $\mathbf{S_1}$ and t_o^{u1} in$\mathbf{S_2}$) in momentum-frequency plane as Δw_1 varies. The trajectories reveal the

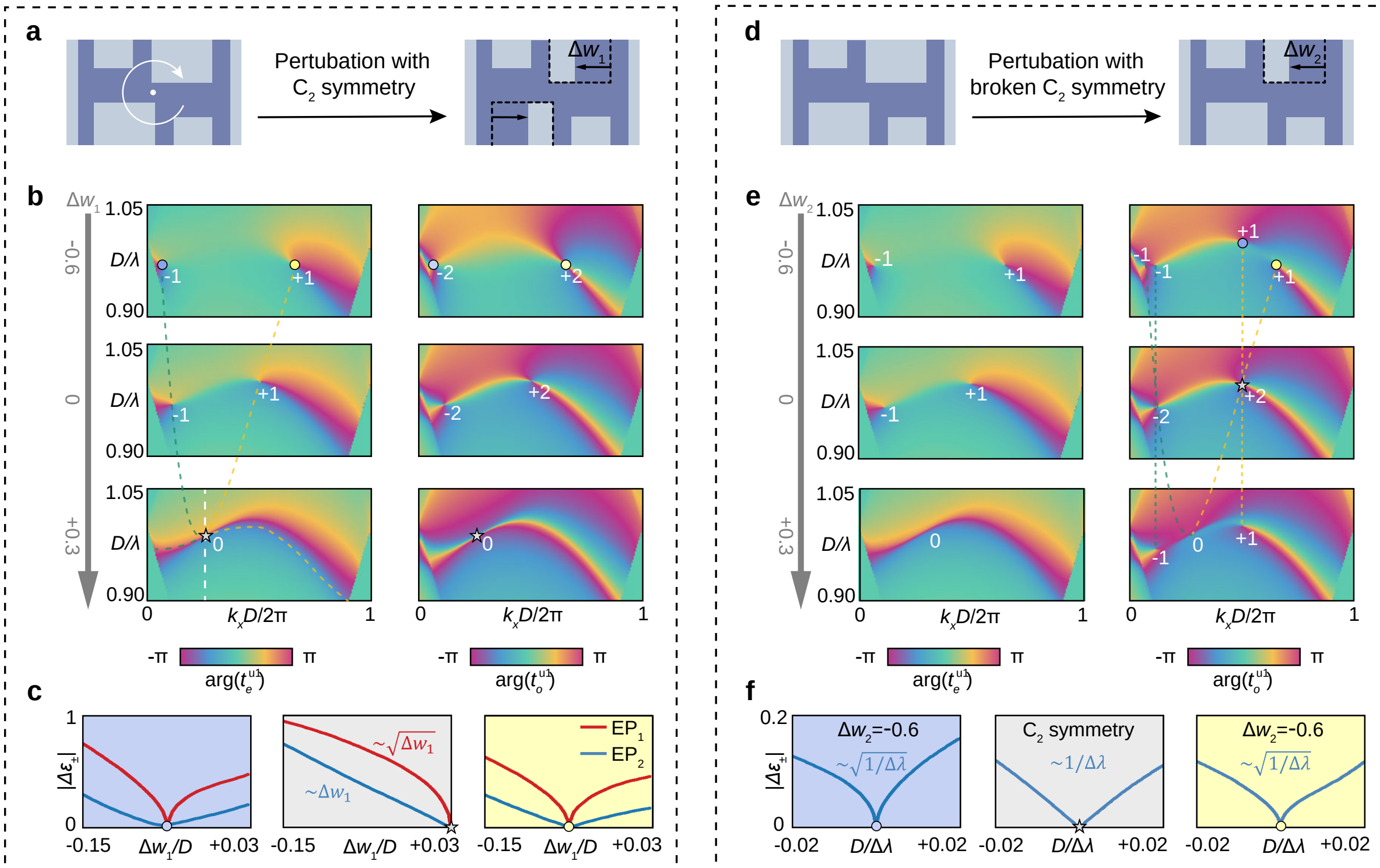

Supplementary Figure 13. **a**, Schematic of the C_2-preserving geometric perturbation Δw_1 introduced to the original metagrating. **b**, Evolution trajectories of the scattering spectral singularities (t_e^{u1} and t_o^{u1}) in momentum–frequency domain with varied Δw_1. The green and yellow dashed lines represent EAs with opposite topological charges undergoing a merging process, during which the locations of EP_1 and EP_2 are anchored together under the constraint of C_2 symmetry. The purple and yellow circles denote pre-merge points with opposite charges, and the gray star indicates the merging point. **c**, Eigenvalue sensitivity variation along Δw_1 for the three representative points marked in **b**. EP_1 and EP_2 exhibit square-root (charge-1) and linear (charge-2) sensitivities, respectively. **d**, Schematic of the C_2-breaking perturbation Δw_2 for comparison. **e**, Evolution of spectral singularities with varied Δw_2. The broken C_2 symmetry causes EP_2 to split into two EPs with identical unit-charges, destroying the location-anchoring between EP_1 and EP_2. **f**, Eigenvalue sensitivity as a function of wavenumber k for the three points marked in **e**. The split EPs possess square-root sensitivities before merging, which transitions to a linear sensitivity at the merging point ($\Delta w_2 = 0$), where C_2 symmetry is restored.

merging of exceptional arcs (EAs) carrying opposite phase-winding charges (denoted by green and yellow dashed lines), obeying the global charge conservation. Throughout this C_2-protected evolution, EA_1 (containing EP_1) and EA_2 (containing EP_2) coincide exactly. Supplementary Figure 13c tracks the eigenvalue sensitivity ($|\epsilon_+ - \epsilon_-|$) as a function of Δw_1 for three representative stages: two pre-merging points with opposite charges (purple and yellow circles in Supplementary Fig. 13b) and the merging point (gray star in Supplementary Fig. 13b). As Δw_1 varies, EP_1 possesses a square-root sensitivity, exhibiting a classic 2-order EP topology, whereas EP_2 possesses a linear sensitivity, which exhibits an unconventional Dirac-like topology.

In order to probe the impact of C_2 symmetry on the coupling between subsystems, we introduce a C_2-breaking perturbation Δw_2, for comparison with Δw_1 (shown in Supplementary Fig. 13d). The evolution of the spectral singularities t_e^{u1} and t_o^{u1} with varied Δw_2 is shown in Supplementary Fig. 13e. In stark contrast to the C_2-preserving case, breaking C_2 symmetry causes EP_2 to split into two fundamental EPs with identical topological charge. In the momentum–frequency domain, one of these split EPs remains stationary, while the other participates in the opposite-charge merging process shown in Supplementary Fig. 13a. This behavior demonstrates that the symmetry-breaking perturbations can induce the merging of projected EPs with identical topological charges, thereby forming a Dirac EP, which aligns with the previous study [6]. We demonstrate this merging process via the eigenvalue sensitivity as a function of wavenumber k (Supplementary Fig. 13f). Prior to merging, the two EPs split from EP_2 exhibit square-root sensitivities. However, at the merging point where $\Delta w_2 = 0$ with the existence of C_2 symmetry, the sensitivity becomes linear. Collectively, these results highlight a paradoxical feature of the EP-STV correspondence: while projected EPs persist over a broad perturbation range due to the topological robustness guaranteed by global charge conservation, their intrinsically divergent sensitivity makes their specific

momentum–frequency coordinates highly susceptible to shifting.

Supplementary Note 8. Eigenvector coalescence of EP_1 and EP_2

In order to investigate the eigenvector features of projected EPs, we show the phase rigidity distribution of EP_1 and EP_2 in Supplementary Fig. 14 obtained from simulated data, which characterizes the degree of "skew" between the eigenvectors, and could be defined by

$$r_{+(-)} = \frac{\langle \tilde{\psi}^L_{+(-)} | \tilde{\psi}^R_{+(-)} \rangle}{\langle \tilde{\psi}^R_{+(-)} | \tilde{\psi}^R_{+(-)} \rangle}, \tag{S40}$$

where $\langle \tilde{\psi}^L_{+(-)}| = \frac{\langle \psi^L_{+(-)}|}{\langle \psi^L_{+(-)} | \psi^L_{+(-)} \rangle}$ and $|\tilde{\psi}^R_{+(-)}\rangle = \frac{|\psi^R_{+(-)}\rangle}{\langle \psi^L_{+(-)} | \psi^L_{+(-)} \rangle}$ are the normalized biorthogonal left eigenvectors and right eigenvectors, and $\langle \psi^L_{+(-)}|$ and $|\psi^R_{+(-)}\rangle$ are left and right eigenvectors corresponding to the eigenvalues. Due to the biorthogonal condition, $\langle \tilde{\psi}^L_{+(-)} | \tilde{\psi}^R_{+(-)} \rangle = \delta_{+-}$ so that the phase rigidity can be expressed as $\frac{1}{\langle \tilde{\psi}^R_{+(-)} | \tilde{\psi}^R_{+(-)} \rangle}$. For a 2-order EP-degeneracy, $r_+ = r_- = 0$, demonstrating the coalescence of two eigenvectors at EP.

It is evident that $r_{+(-)}$ approaches 0 at EP_1 and EP_2 simultaneously, shown in Supplementary Fig. 14a and 14c. However, the trends of phase rigidity near EP_1 and EP_2 are strikingly different, displayed by Supplementary Fig. 14b and 14d. For EP_1, the phase rigidity exhibits a square-root behavior (the approximately 1/2- slope in logarithmic coordinate, the inset of Supplementary Fig. 14b), demonstrating its typical EP-like topological characteristics. For EP_2, the phase rigidity exhibits a linear behavior (the approximately 1- slope in logarithmic coordinate, the inset of Supplementary Fig. 14d), demonstrating its Dirac-like topological features, like Ref[6].

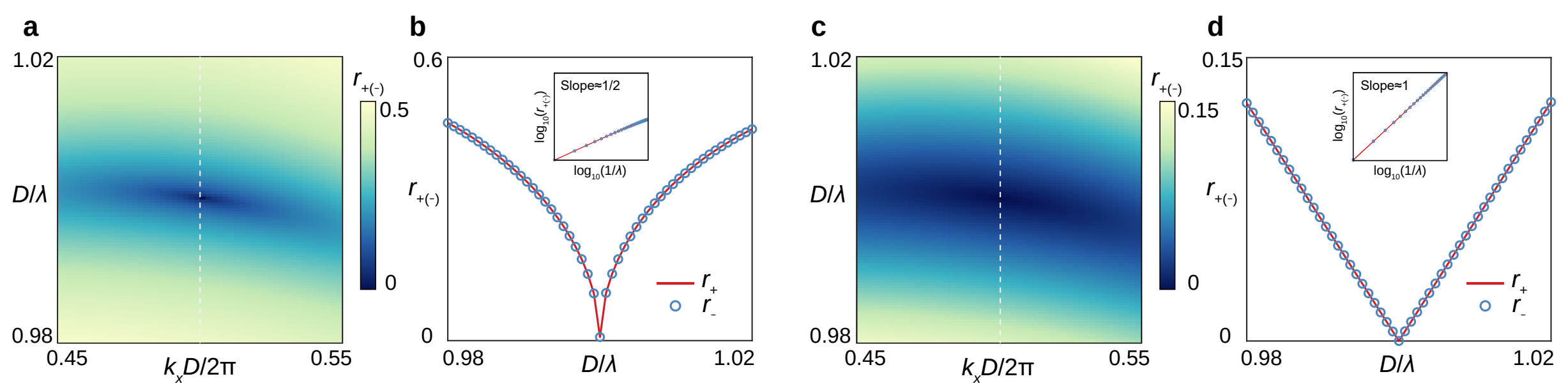

Supplementary Figure 14. Simulated phase rigidity $r_{+(-)}$ of eigenvectors around EP_1 (**a**) and EP_2 (**c**) in momentum-frequency domain. **b** and **d** are results extracted from **a** and **c** (white dash line), the insets exhibit the logarithmic plots. The *x*- and *y*-axis ranges in logarithmic insets of **b** and **d** are the same.

Supplementary Note 9. Extended simulation in acoustic system

For validating the universality of our idea in diverse wave system, we design another metagrating structure via acoustic system. The metagrating's configuration aligns with the structure of liquid-surface-wave system in main text, the geometric parameters of which are shown in Table 2. In acoustic system, free wave propagates with a linear cone-type dispersion, different from the liquid surface wave system. Whereas our structure design depends solely on the target wavelength and is dispersion-independent, it remains equally applicable in linearly dispersive wave systems (such as acoustic and optics system). By the design, we set EP_1 and EP_2 at $(\frac{\pi}{D}, \frac{\sqrt{2}\pi c}{D})$ in k_x-ω space, c is the sound velocity, so that the operated frequency f_0 is 7274.7 Hz and the operated incident angle θ_0 is 45°. Supplementary Figures 15a-d exhibit the topological structure of EP_1 and EP_2, and the singularities of scattering spectrum t_e^{u1} and t_o^{u1} in acoustic-based paradigm, similar to the results in Fig. 2 of the main text. Likewise, the simulated time sequences of field patterns including the generation of charge-1 anomalous-transmitted STV (EP_1) and disintegrated charge-2 specular-transmitted STV (EP_2) are shown by Supplementary Fig. 15e. Notably, the transmitted angles of STVs in Supplementary Fig. **??**e are ±45°, differing from the 30° mentioned in the main text. This demonstrates that our method can readily generate the spatiotemporal vortex scattering at arbitrary angles, showcasing the enormous potential in structured waves manipulation.

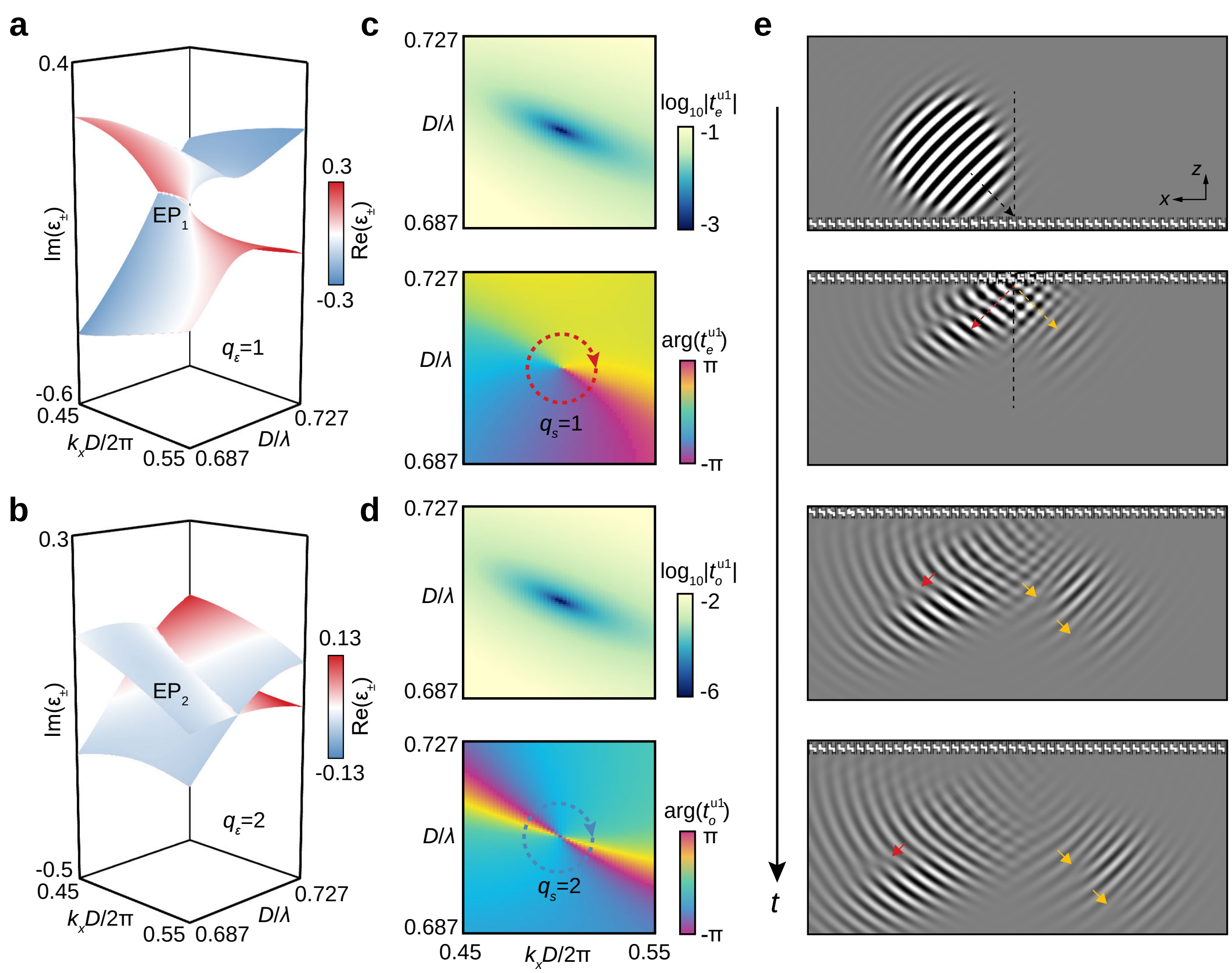

Supplementary Figure 15. EP-STV correspondence in acoustic system. **a-b**, Riemann surface of EP_1 and EP_2. **c-d**, Amplitude and phase map of t_e^{u1} and t_o^{u1}, exhibiting the topological charges of EP_1 ($q_s = 1$) and EP_2 ($q_s = 2$). **e**, The simulated time sequences of field patterns, displaying the dynamic scattering picture of EP_1 and EP_2 in acoustic system.

Supplementary Table 2: Parameters of metagrating structure in acoustic system

D (cm)	h_c (cm)	h_1 (cm)	h_2 (cm)	w_c (cm)	w_1 (cm)	w_2 (cm)	d_1 (cm)	d_2 (cm)	d_3 (cm)
33.34	4.21	0.62	1.57	0.70	0.70	1.50	0.21	0.18	0.04

Supplementary References

1. Díaz-Rubio, A. , &Tretyakov, S. A. Aoustic metasurfaces for scattering-free anomalous reflection and refraction.. *Phys. Rev. B* **96**, 125409 (2017).

2. Hou, Z., Fang, X., Li Y., &Assouar, B. Highly efficient acoustic metagrating with strongly coupled surface grooves. *Phys. Rev. Appl.* **12**, 034021 (2019).

3. Tang, W., Ding, K. & Ma, G. Direct Measurement of Topological Properties of an Exceptional Parabola. *Phys. Rev. Lett.* **127**, 034301 (2021).

4. Shen, H., Zhen, B. & Fu, L. Topological Band Theory for Non-Hermitian Hamiltonians. *Phys. Rev. Lett.* **120**, 146402 (2018).

5. Wildeman, S. Real-time quantitative Schlieren imaging by fast Fourier demodulation of a checkered backdrop. *Exp. Fluids* **59**, 97 (2018).

6. Wu, Y., Zhu, D., Wang, Y., Rong, X. & Du, J. Experimental Observation of Dirac Exceptional Points. *Phys. Rev. Lett.* **134**, 153601 (2025).
