# Supplementary material for: Spatiotemporal dynamics of scattering exceptional points
Source: Nat Commun. 2026 Jul 7;17:8373. doi: 10.1038/s41467-026-75187-2 (PMC13473472; doi:10.1038/s41467-026-75187-2)
Supplement: Supplementary file 2 [file 41467_2026_75187_MOESM2_ESM.pdf]

Description of Additional Supplementary Files

Supplementary Data 1-3: Time-domain signal corresponding to Fig. 4f in the main text. Data 1 is the incident pulse, Data 2 is the anomalous-transmitted pulse, and Data 3 is the ordinary-transmitted pulse. Columns represent time (t, in seconds) and the real part of the measured amplitude [Re(P), in arbitrary units (arb. units)].

Supplementary Data 4-5: Real-part spatial field patterns (in arb. units) corresponding to Fig. 4g, for the anomalous-transmitted (Data 4) and ordinary-transmitted (Data 5) pulses. The row and column coordinates denote relative spatial positions.

Supplementary Data 6-7: Real-part spatial field patterns (in arb. units) corresponding to Fig. 4h, for the anomalous-transmitted (Data 6) and ordinary-transmitted (Data 7) pulses.

Supplementary Data 8 and 10: Real-part spatial field patterns (in arb. units) corresponding to Fig. 4i, for the anomalous-transmitted (Data 8) and ordinary-transmitted (Data 10) pulses.

Supplementary Data 9 and 11: Phase distributions (in radians) of local pulse envelope corresponding to insets in Fig. 4i, for the anomalous-transmitted (Data 9) and ordinary-transmitted (Data 11) pulses.
