# Supplementary material for: Spatiotemporal dynamics of scattering exceptional points
Source: Nat Commun. 2026 Jul 7;17:8373. doi: 10.1038/s41467-026-75187-2 (PMC13473472; doi:10.1038/s41467-026-75187-2)
Supplement: Supplementary file 3 [file 41467_2026_75187_MOESM4_ESM.pdf]

nature portfolio

Peer Review File

Spatiotemporal Dynamics of Scattering Exceptional Points

Corresponding Author: Professor Jie Zhu

This file contains all reviewer reports in order by version, followed by all author rebuttals in order by version.

Version 0:

Reviewer comments:

Reviewer #1

(Remarks to the Author)
The manuscript has investigated the dynamic consequences of scattering exceptional points (S-EPs) in non-Hermitian wave systems. The authors have proposed a framework that connects the topology of EPs defined in momentum–frequency space with spatiotemporal singularities in real space-time. Specifically, they have demonstrated that when a metagrating supporting scattering EPs is excited by a pulsed wave packet, the exceptional topology can be transferred to the scattered wave in the form of spatiotemporal vortices (STVs) with quantized topological charge. Using a carefully designed metagrating platform, the authors have analyzed two distinct EPs with different topological charges and have shown that these EPs give rise to corresponding STV structures in the scattered field. Experimental measurements using a liquid-surface-wave platform have been presented to visualize these spatiotemporal phase singularities, including the observation of a charge-1 STV and a higher-order charge-2 structure that has manifested as sequential charge-1 vortices.

In my assessment, the manuscript presents an interesting and conceptually appealing idea. This work is likely to be of significance to researchers working in non-Hermitian physics, wave dynamics, and topological wave phenomena, as well as to related communities investigating the physics of structured light. The manuscript is well organized and clearly written. The methodology, including the theoretical formulation, numerical simulations, and experimental implementation, appears to be sound and consistent with the expected standards in this field. The figures are particularly impressive and effectively convey the main concepts. The results presented appear to support the manuscript's main conclusions and claims. Although some sections of the main text appear somewhat compressed, they remain understandable with the help of the Supplementary Information.

My comments are as follows.

Major comments:

1. While the manuscript introduces the distinction between Hamiltonian exceptional points (H-EPs) and scattering exceptional points (S-EPs), the discussion in the Introduction could be clarified further. In particular, the text around Page 2 (lines 18–22) and Page 3 (lines 1–3) may give the impression that H-EPs are always associated with closed systems while S-EPs correspond to open systems, which could be somewhat misleading (especially to unfamiliar readers). In practice, EPs arise from effective non-Hermitian Hamiltonians involving gain, loss, or radiative leakage. A clearer explanation of this distinction would strengthen the study's conceptual motivation.

2. Exceptional points are typically described through the coalescence of eigenvalues of an underlying non-Hermitian Hamiltonian. In the present work, however, the EPs correspond to the eigenvalues of the scattering submatrices S_1 and S_2, which arise from projected subspaces of the full scattering matrix. In this context, do the observed topological features remain invariant with respect to different choices of basis or subsystem decomposition?

3. While the theoretical derivation provides an intuitive and appealing explanation of this correspondence, the analysis relies on certain approximations (e.g., the local phase-vortex description of the scattering coefficient near the EP and the neglect of anisotropy in the phase distribution). It would be beneficial if the authors could clarify more explicitly the range of validity of this approximation and discuss how robust the EP–STV mapping remains under realistic conditions where anisotropy or spectral distortions may be present.

4. Regarding the generation of a charge-2 STV, the authors could elaborate on whether the observed structure should be

interpreted as a genuine higher-order STV that subsequently splits, or simply as two closely related first-order vortices.

5. The experimental confirmation of the topological charge appears somewhat qualitative (although visually convincing). Can the authors provide a quantitative extraction of topological charge from the experimental phase maps, rather than just visual phase behaviors?

Minor comments:
1. A few long sentences in the Introduction could be slightly shortened to improve flow.
2. There might be some typos (e.g., ‘metrix’ on page 11).
3. In Fig. 3, it would be helpful to include color bars for clarity

Reviewer #2

(Remarks to the Author)
The authors study scattering exceptional points (S-EPs) with their dynamic manifestations in the form of spatiotemporal vortices (STVs). They establish a clear EP–STV correspondence and provide compelling experimental evidence using a liquid-surface-wave platform. The work is timely, technically sound, and of broad interest to the spatiotemporal physics, wave dynamics, and metamaterials communities. The manuscript is generally well written and the results are convincing. However, several points require clarification or further elaboration before the paper can be considered for publication.

1. The current formulation of Eq.(1) makes it difficult for readers to clearly interpret how the 4-port scattering matrix is partitioned into the two 2×2 subsystems S1 and S2. Since these two subsystems are central to the emergence of the distinct EPs (charge-1 and charge-2), I strongly suggest that the authors explicitly define S1 and S2 in the main text, rather than relying on readers to infer them from Eq.(1).

2. The manuscript states that the metagrating preserves C2 rotational symmetry, but the axis of this C2 rotation is never explicitly stated. This is important because the projection of the full 4-port scattering matrix into two decoupled 2×2 subspaces (S1 and S2) and the existence of two independent EPs both rely critically on this symmetry. The authors should explain how this symmetry maps the four ports into two symmetric pairs, and why this guarantees that S1 and S2 remain decoupled. Does slight symmetry breaking would couple the two subsystems or modify the EP structure?

Reviewer #3

(Remarks to the Author)
The manuscript presents an analysis of projected scattering exceptional points (EPs) generated during spatiotemporal dynamics, along with their experimental implementation on a liquid surface wave platform, revealing spatiotemporal vortices.

Although the experimentally realized system described in the article is closed and Hermitian—and thus evolves unitarily—a projection onto its subsystem(s) leads to an effective non-Hermitian description, with the resulting dynamics becoming non-unitary which enables the observation of EPs. In this sense, the projection of the 4×4 scattering matrix S_p, given in Eq. (1), onto two-by-two scattering sub-matrices S_1 and/or S_2 can be interpreted as treating these subspaces as the system of interest, while the remaining degrees of freedom act as an environment, in line with standard descriptions of losses, for example via a master equation. The sub-matrices S_1 and S_2 are non-Hermitian and can therefore exhibit singularities (e.g., EPs), as studied in the manuscript.

The authors claim to establish a direct cross-domain correspondence linking a static EP to a dynamic scattering singularity. While this is an interesting perspective, connections between static and dynamic EPs (defined in various ways) have been studied previously. For example, EPs of a non-Hermitian Hamiltonian (as mentioned in the manuscript and referred to as Hamiltonian EPs, H-EPs) are typically interpreted as static EPs, whereas EPs of a Liouvillian (referred to as Liouvillian EPs, L-EPs) correspond to dynamic EPs. The relation between H-EPs and L-EPs can be formulated within a hybrid (or q-deformed) Liouvillian framework [Minganti2020, Kumar2021], which was demonstrated experimentally in [Chen2021].

Overall, I find the presented experimental results interesting, novel, and potentially of significant interest to the readership of Nature Communications. However, before recommending publication, I suggest several improvements and clarifications, including citing a few closely related papers. Specifically,

(1) I suggest explicitly indicating in the title that the work concerns projected EPs, i.e., EPs obtained by projecting the full unitary, non-singular scattering matrix S_p onto lower-dimensional 2×2 subspaces. As stated in the manuscript, the intrinsic loss in the experimental metagrating is negligible; therefore, EPs cannot occur in the full matrix S_p. However, they can emerge in its nonunitary subspaces, as demonstrated by the authors. Thus, the title could be revised by adding the word "Projected," for example: “Spatiotemporal Dynamics of Projected Scattering Exceptional Points.”

This is an important conceptual point and should be emphasized not only in the title but also in the abstract—namely, that the observed EPs arise from projections of the unitary scattering matrix onto lower-dimensional subspaces.

(2) The authors emphasize that the novelty of the paper lies in its treatment of spatiotemporal dynamics. In this context, I suggest that the authors cite related works on spatiotemporal dynamics, for example in photonic crystals [Sharabi2022, Bushuev2026], where exceptional points have also been studied.

(3) The use of terms such as “revolutionized” or “groundbreaking” appears unnecessary and somewhat overstated. For example, the statement that “exceptional points have revolutionized the control of wave–matter interactions” seems exaggerated, even though exceptional points have indeed attracted considerable interest. Note, e.g., that it remains under debate whether they can enhance sensing or precision of measurements, and there are many works indicating that this may not be the case.

Similarly, referring to demonstrations of Hamiltonian exceptional points as “groundbreaking” may be too strong. A more balanced and precise wording would improve the tone of the manuscript.

(4) The first sentence of the abstract refers to non-Hermitian physics and cites two papers by Carl Bender et al. While these works are certainly important—particularly in enabling the analysis of non-Hermitian Hamiltonians (NHHs) in terms of parity–time (PT) symmetry—non-Hermitian physics has been central to quantum research long before the 1998 paper by Bender. Indeed, non-Hermitian systems have been studied for over 60 years, typically in the context of Markovian or non-Markovian master equations (i.e., Liouvillians) or Heisenberg–Langevin equations of motion.

Moreover, NHHs have been widely used since the 1990s within the quantum trajectory approach to solve master equations [Carmichael1993, Dalibard1992]. The similarities and differences between NHHs and Liouvillians underpin the relationship between the above-mentioned H-EPs and L-EPs.

In this context, I would suggest simplifying the phrase “a foundational framework for controlling wave–matter interactions” when referring to Bender's works to simply “a framework,” which would provide a more balanced and historically accurate description.

(5) Degeneracies in the spectra of matrices do not necessarily correspond to EPs; in general, they may also correspond to diabolical points [Berry1984, Arkhipov2023], for which the corresponding eigenvectors are orthogonal. I assume that the authors have verified not only the eigenvalues but also the eigenvectors at the identified singularities, and that all the reported cases indeed correspond to exceptional points for which the corresponding eigenvectors are coalescent. Nevertheless, it would be helpful to include a clear statement confirming that no diabolical points are observed in the system.

(6) Moreover, Fig. 2 shows a Dirac-like degeneracy. Such a degeneracy does not necessarily correspond to an exceptional point; it may instead represent a diabolical point—the concept originally introduced by Michael Berry [Berry1984], named for its resemblance to a diabolo toy. This raises the question of whether the authors have verified the nature of the corresponding eigenvectors. In particular, it would be important to check whether the eigenvectors coalesce, as expected for exceptional points, or remain orthogonal, as is typical for diabolical points.

(7) The input and output wave vectors on the left- and right-hand sides of Eq. (1) should be distinguished by different symbols. Currently, they are denoted by the same wave vectors, which is misleading.

(8) It is somewhat unfortunate, from the perspective of EP studies, that the full \$4 \times 4\$ scattering matrix is unitary in their experiment. If it were nonunitary, the system could allow for a richer structure of spectral degeneracies, including exceptional points, diabolical points, and possibly hybrid exceptional–diabolical degeneracies in a genuinely lossy setting.

Thus, he question arises whether it is experimentally feasible to introduce controlled losses into the \$4 \times 4\$ system such that the resulting scattering matrix becomes non-unitary in the platform used in the manuscript. I am not suggesting that such extended experiments are necessary, but I believe it would be valuable for the reader to hear some comments on a possible generalization of the experiment—one that incorporates genuine losses in the \$4 \times 4\$ system, rather than effectively simulating losses by restricting attention to a sub-block of a larger unitary matrix \$S_p\$.

(9) The analysis of sub-matrices of a unitary \$4 \times 4\$ matrix considerably simplifies the problem, not only from a mathematical perspective but also in terms of physical interpretation. The authors focus on two sub-matrices, \$S_1\$ and \$S_2\$, presented on page 8. However, many other sub-matrices could be considered, some of which may also exhibit exceptional points. While I do not insist that the authors provide a more detailed analysis of additional sub-matrices, a few clarifying remarks would be beneficial for the reader. In particular, it would be helpful to explain why these two specific sub-matrices were chosen, and whether other sub-matrices may display interesting nontrivial features, including exceptional points and possibly the emergence of diabolical points.

(10) The authors state that "EPs are associated with the evolution of confined modes in closed spaces." on page 2. However, in most cases, EPs are demonstrated in open systems that are either dissipative or include gain. While there are theoretical predictions of non-Hermitian dynamics in effectively non-dissipative systems—for example, under the presence of squeezing [Wang2019, Wakefield2024]—I find the current statement in the manuscript somewhat misleading. I do not expect the authors to cite additional references on such systems, but it would be preferable to clarify that EPs are typically associated with open systems, in contrast to the description given here and, for instance, in Ref. [43].

(11) As mentioned above, it is worth noting in the manuscript that EPs are not limited to singularities of Hamiltonians or scattering matrices. Hamiltonian EPs, which neglect thermal and quantum jump processes, can be generalized to Liouvillian EPs in a full quantum description. Liouvillian exceptional points (L-EPs) have been introduced for both Markovian [Minganti2019] and non-Markovian [Lin2025] systems, and have been experimentally demonstrated on various platforms, including circuit QED [Chen2021, Chen2022, Zhang2025] and ion traps [Zhang2022, Bu2023, Bu2024].

(12) Various types of singularities of scattering matrices—including exceptional points, as well as diabolical points and hybrid exceptional–diabolical degeneracies—have been experimentally observed (see, e.g., [Erb2025, Shaibe2025] and the references therein).

In conclusion, I find the paper interesting, especially its experimental results. With appropriate clarifications and by citing some of the references mentioned here, I would be able to recommend the manuscript for publication in Nature Communications.

-- I include the following references for completeness of the report, but I do not require that all of them be cited in the manuscript.

[Minganti2020] F. Minganti et al., Hybrid-Liouvillian formalism connecting exceptional points of non-Hermitian Hamiltonians and Liouvillians via postselection of quantum trajectories, Phys. Rev. A 101, 062112 (2020).

[Kumar2021] P. Kumar, K. Snizhko, and Y. Gefen, Near-unit efficiency of chiral state conversion via hybrid-Liouvillian dynamics, Phys. Rev. A 104, L050405 (2021).

[Chen2021] W. Chen et al., Quantum Jumps in the Non-Hermitian Dynamics of a Superconducting Qubit Phys. Rev. Lett. 127, 140504 (2021).

[Sharabi2022] Y. Sharabi et al., Spatiotemporal photonic crystals, Optica 9, 585 (2022).

[Bushuev2026] V. A. Bushuev and B. I. Mantsyzov, Spatiotemporal dynamics of formation and propagation of spectrally singular modes in a broadband quasi-symmetric photonic crystal, Phys. Rev. A 113, 033510 (2026).

[Carmichael1993] H. J. Carmichael, Quantum Trajectory Theory for Cascaded Open Systems, Phys. Rev. Lett. 70, 2273 (1993).

[Dalibard1992] J. Dalibard, Y. Castin, and K. Molmer, Wave-Function Approach to Dissipative Processes in Quantum Optics, Phys. Rev. Lett. 68, 580 (1992).

[Arkhipov2023] I. I. Arkhipov et al., Dynamically encircling an exceptional curve by crossing diabolic points: A programmable multimode switch, Nat. Comm. 14, 2076 (2023)

[Berry1984] M. V. Berry and M. Wilkinson. “Diabolical points in the spectra of triangles”. Proc. R. Soc. London A 392, 15–43 (1984).

[Wang2019] Y.-X. Wang and A. A. Clerk, Non-Hermitian dynamics without dissipation in quantum systems, Phys. Rev. A 99, 063834 (2019).

[Wakefield2024] R. Wakefield, A. Laing, Y. N. Joglekar, Non-Hermiticity in quantum nonlinear optics through symplectic transformations, Appl. Phys. Lett. 124, 201103 (2024).

[Minganti2019] F. Minganti et al., Quantum exceptional points of non-Hermitian Hamiltonians and Liouvillians: The effects of quantum jumps, Phys. Rev. A 100, 062131 (2019).

[Lin2025] J.-D. Lin et al., Non-Markovian Quantum Exceptional Points, Nature Commun. 16, 1289 (2025).

[Chen2022] W. Chen et al., Decoherence Induced Exceptional Points in a Dissipative Superconducting Qubit, Phys. Rev. Lett. 128, 110402 (2022).

[Zhang2022] J.-W. Zhang et al., Dynamical control of quantum heat engines using exceptional points, Nat. Commun. 13, 6225 (2022).

[Bu2023] J.-T. Bu et al., Enhancement of Quantum Heat Engine by Encircling a Liouvillian Exceptional Point, Phys. Rev. Lett. 130, 110402 (2023).

[Bu2024] J.-T. Bu et al., Chiral quantum heating and cooling with an optically controlled ion, Light: Sci. App. 13, 143 (2024).

[Zhang2025] H.-L. Zhang et al., Experimental Observation of Non-Markovian Quantum Exceptional Points, Phys. Rev. Lett. 135, 230203 (2025).

[Erb2025] J. Erb et al., Topology and manipulation of scattering singularities in complex non-Hermitian systems: Two-channel case Phys. Rev. Res. 7, 023090 (2025).

[Shaibe2025] N. Shaibe et al., Superuniversal Statistics of Complex Time Delays in Non-Hermitian Scattering Systems, Phys. Rev. Lett. 134, 147203 (2025).

Version 1:

Reviewer comments:

Reviewer #1

(Remarks to the Author)
I am overall satisfied with the authors’ response to my comments and would like to support publication of the manuscript in Nature Communications.

Reviewer #2

(Remarks to the Author)
The authors have revised the manuscript in accordance with the reviewers' comments. I only have two minor remarks regarding the revision:

1. The present manuscript demonstrates the STV using a mirror-symmetry-breaking slab, which is closely related to the recent experimental work Light 15, 77 (2026).

2. There is a typo in Fig. S4: the bottom label “Port u” should be “Port d”.

Reviewer #3

(Remarks to the Author)
The authors have thoroughly revised the manuscript and have satisfactorily addressed the concerns raised in my first-round report, which was approximately 1900 words long. In addition, they have provided a very detailed and highly informative 49-page rebuttal letter, in which they carefully respond to the comments and questions of all three referees.

The revised manuscript represents a significant improvement over the original submission. The presentation is clearer, the discussion is better structured, and the main results are now more accessible to the reader. The authors have substantially strengthened the discussion of the novelty of their approach and its advantages over existing methods or even definitions of exceptional points (to mention, e.g., Liouvillian quantum exceptional points), thereby making the scientific significance and potential impact of the work much more evident.

As an example, I would like to mention my suggestion to make the study more complete by analyzing exceptional points associated with additional submatrices obtained by projecting the unitary scattering matrix onto lower-dimensional subspaces. This comment has been taken seriously by the authors, who have included a substantial amount of new material in the Supplementary Information. A similar request was also raised by another referee, and the authors have responded comprehensively by significantly extending both their analysis and discussion.

Although I have not independently verified every technical aspect of the analysis in full detail, the results appear to be physically sound and internally consistent. Moreover, the authors' responses to the referees' comments are thoughtful, comprehensive, and convincing.

In view of these improvements, I believe that the manuscript meets the high standards of Nature Communications, and I am pleased to recommend its publication.

Open Access This Peer Review File is licensed under a Creative Commons Attribution 4.0 International License, which permits use, sharing, adaptation, distribution and reproduction in any medium or format, as long as you give appropriate credit to the original author(s) and the source, provide a link to the Creative Commons license, and indicate if changes were made.
In cases where reviewers are anonymous, credit should be given to 'Anonymous Referee' and the source.
The images or other third party material in this Peer Review File are included in the article's Creative Commons license, unless indicated otherwise in a credit line to the material. If material is not included in the article's Creative Commons license and your intended use is not permitted by statutory regulation or exceeds the permitted use, you will need to obtain permission directly from the copyright holder.
To view a copy of this license, visit https://creativecommons.org/licenses/by/4.0/

Response to Referee 1 -- NCOMMS-26-011336-T

Reviewer:

The manuscript has investigated the dynamic consequences of scattering exceptional points (S-EPs) in non-Hermitian wave systems. The authors have proposed a framework that connects the topology of EPs defined in momentum–frequency space with spatiotemporal singularities in real space-time. Specifically, they have demonstrated that when a metagrating supporting scattering EPs is excited by a pulsed wave packet, the exceptional topology can be transferred to the scattered wave in the form of spatiotemporal vortices (STVs) with quantized topological charge. Using a carefully designed metagrating platform, the authors have analyzed two distinct EPs with different topological charges and have shown that these EPs give rise to corresponding STV structures in the scattered field. Experimental measurements using a liquid-surface-wave platform have been presented to visualize these spatiotemporal phase singularities, including the observation of a charge-1 STV and a higher-order charge-2 structure that has manifested as sequential charge-1 vortices.

In my assessment, the manuscript presents an interesting and conceptually appealing idea. This work is likely to be of significance to researchers working in non-Hermitian physics, wave dynamics, and topological wave phenomena, as well as to related communities investigating the physics of structured light. The manuscript is well organized and clearly written. The methodology, including the theoretical formulation, numerical simulations, and experimental implementation, appears to be sound and consistent with the expected standards in this field. The figures are particularly impressive and effectively convey the main concepts. The results presented appear to support the manuscript's main conclusions and claims. Although some sections of the main text appear somewhat compressed, they remain understandable with the help of the Supplementary Information.

Response:

Thank you for the careful review of our manuscript and the positive evaluation. Your comments have greatly contributed to strengthening our work, and we have addressed each of them below.

Reviewer:

Major comments:

1. While the manuscript introduces the distinction between Hamiltonian exceptional points (H-EPs) and scattering exceptional points (S-EPs), the discussion in the Introduction could be clarified further. In particular, the text around Page 2 (lines 18 – 22) and Page 3 (lines 1 – 3) may give the impression that H-EPs are always associated with closed systems while S-EPs correspond to open systems, which could be somewhat misleading (especially to unfamiliar readers). In practice, EPs arise from effective non-Hermitian Hamiltonians involving gain, loss, or radiative leakage. A clearer explanation of this distinction would strengthen the study's conceptual motivation.

Response:

Thank you for this professional and rigorous comment. We completely agree that our original phrasing could inadvertently be misleading. You are right, the fundamental distinction between H-EPs and S-EPs lies not in whether the system is open or closed (since both can describe open systems interacting with the environment), but rather in the physical observables they characterize, i.e. **intrinsic eigenstate (H-EPs) vs. input-output scattering responses (S-EPs)**. The H-EP picture describes the system itself, corresponding to the coalescence of the system's intrinsic eigenstates. In contrast, the S-EP picture characterizes the system's external input-output relationship, corresponding to the degeneracies of the scattering matrix at a real driving frequency, which govern the anomalous scattering behaviors in the far field. It has been shown that H-EP representation could not accurately describe the multi-port behaviors of a metagrating (Refs [14-17] in the manuscript), while S-EP can serve as a complement to H-EP in describing the degeneracy of scattering modes in multi-port scattering systems (Refs [25-29]).

In light of your valuable comment, we have carefully revised the Introduction to eliminate any ambiguity:

Changes in the abstract (page 1, lines 15-17) in revised manuscript:

"While traditional EPs are associated with the Hamiltonians, characterizing the coalescence of intrinsic eigenstates, scattering EPs shift the perspective toward input-output responses, offering a powerful framework for wave control in free space. "

Changes in the introduction (page 2, lines 21-22 and page 3, lines 1) in revised manuscript:

"Conventionally, these demonstrations predominantly revolve around Hamiltonian EPs (H-EPs), which emerge as degeneracies of the non-Hermitian effective Hamiltonian, associated with the coalescence of intrinsic eigenstates."

Changes in the introduction (page 3, lines 4-7) in revised manuscript:

"When considering multi-port scattering problems, nevertheless, Hamiltonian could not directly describe scattering modes behaviors[18]. Open structures such as metagratings[5,25] are more appropriately modeled by a scattering matrix, which fully characterizes the wave-structure interactions in free space. In light of this, the Hamiltonian-centric view has been fruitfully complemented by the concept of the scattering EP (S-EP)[28,29].…"

Reviewer:

2. Exceptional points are typically described through the coalescence of eigenvalues of an underlying non-Hermitian Hamiltonian. In the present work, however, the EPs correspond to the eigenvalues of the scattering submatrices S_1 and S_2, which arise from projected subspaces of the full scattering matrix. In this context, do the observed topological features remain invariant with respect to different choices of basis or subsystem decomposition?

Response:

This is a very good question.

Firstly, regarding the choice of basis within a fixed subspace, the observed topological features (such as the complex eigenvalue degeneracy and the associated winding numbers) are invariant under any unitary transformation. Since the eigenvalues of the scattering submatrices are the primary observables defining the S-EPs, **a change of basis within the subspace performs a similarity transformation on the submatrix**, leaving its eigenvalues and the condition for coalescence (the EP) unchanged.

On the other hand, the choice of subsystem decomposition is not an arbitrary mathematical partition; it corresponds to a specific set of physical excitation and observation channels. Therefore, if the subsystem decomposition is fundamentally changed, the observed topological features generally will not remain invariant. Rather than being a limitation, this dependence highlights a key physical property of projected-EPs: **channel-selectivity**. The existence of an EP in a specific subspace implies that these topological features can only be excited by the specific eigenstates (i.e., generally a specific combination of input channels) corresponding to that chosen subsystem.

According to your comment, **we have added a new section (Supplementary Note 3 "Constructing rule of projected EP") to explain this in more detail.** Based on the intrinsic symmetries of the metagrating system, the full 4-dimensional scattering matrix $\mathbf{S_p}$ can be mathematically projected into various 2-dimensional sub-matrices. By selecting 2 out of the 4 input ports and 2 out of the 4 output ports, there are in total 36 possible 2×2 sub-matrices. However, due to the strict mathematical requirement for the emergence of an EP (i.e., forming a non-diagonalizable Jordan block), **the number of subsystems capable of hosting a projected EP is far fewer than 36**. We show that there are only two pairs of sub-matrices supporting the projected EPs: $\mathbf{S_1}$ and $\mathbf{S_3}$ (supporting EP_1), and $\mathbf{S_2}$ and $\mathbf{S_4}$ (supporting EP_2). We compared the projected EPs (EP_1 and EP_2) induced by different sub-matrices ($\mathbf{S_1}$ and $\mathbf{S_2}$ in main text vs. $\mathbf{S_3}$ and $\mathbf{S_4}$ in Supplementary Note 3), which share identical eigenvalue topologies but possess different matrix basis choices. Despite the eigenvalue Riemann surfaces not being exactly the same, EP_1 (EP_2) derived by $\mathbf{S_1}$ and $\mathbf{S_3}$ ($\mathbf{S_2}$ and $\mathbf{S_4}$) carries the identical topological invariant of $q_\epsilon = 1$ ($q_\epsilon = 2$).

Below are the modifications we have made based on your comments:

Changes (page 9, lines 17-21) in revised manuscript:

"Note that the observed topological features are tied to the specific choice of subsystem decomposition, as each subsystem corresponds to a distinct set of physical excitation and observation channels. Hence, the existence of EP_1 and EP_2 in the specific subspaces implies that their topological features can only be excited by the eigen-input states corresponding to those chosen subsystems"

Added part in Supplementary Information as Supplementary Note 3:

Constructing rule of projected EP

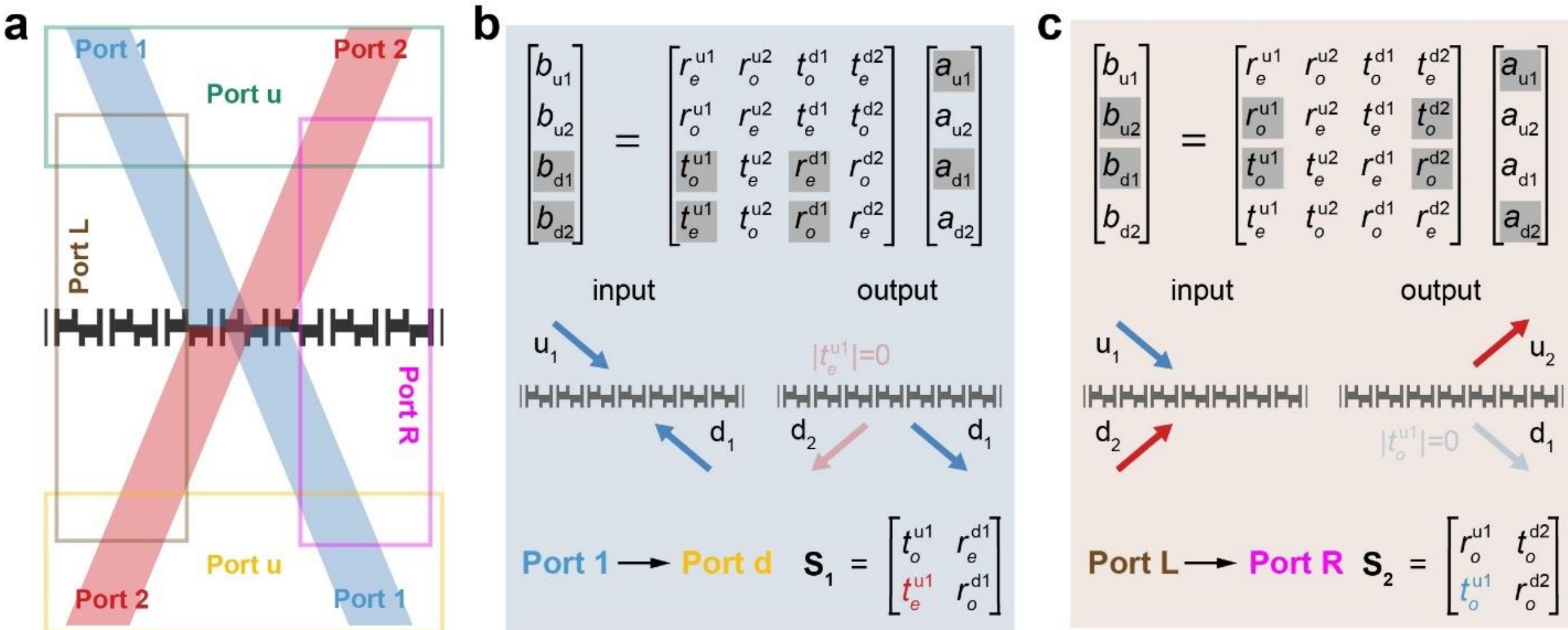

Figure S4 **a**, The full space is divided into six 2-dimensional subspaces by the intrinsic symmetry of the metagrating. Specifically, ports u and d (vertical half spaces, colored by green and yellow), ports 1 and 2 (diagonal half-spaces, colored by blue and red), ports L and R (horizontal half spaces, colored by brown and magenta). **b** and **c**, Detailed projection process and input-output behaviors of $\mathbf{S_1}$ and $\mathbf{S_2}$ mentioned in the main text.

For the designed metagrating system shown in the main text, the element-wise absolute value of its full scattering matrix takes the form abs($\mathbf{S_p}$) = [1, 0, 0, 0; 0, 0, 0, 1; 0, 0, 1, 0; 0, 1, 0, 0]. Here we divide the full space with six 2-dimensional subspaces by the intrinsic symmetry of the metagrating, shown by Fig. S4a. To be specific, we divide the full 4-dimentional space into paired subspaces, including the diagonal and anti-diagonal half-spaces defined by C_2 rotation symmetry as port 1 and port 2, the upper and lower half-spaces defined by up-down mirror symmetry as port u and port d, and the left and right half-spaces defined by left-right mirror symmetry as port L and port R. In principle, by selecting 2 out of the 4 input ports and 2 out of the 4 output ports, there are in total 36 possible 2×2 sub-matrices (i.e. 36 2-dimentional subspaces). However, due to the strict mathematical requirement for the emergence of an EP (i.e., forming a non-diagonalizable Jordan block), the number of subsystems capable of hosting a projected EP is far fewer than 36. Mathematically, a second-order Jordan block requires that only one of its off-diagonal elements vanishes. Given that the scattering matrix $\mathbf{S_p}$ of our designed metagrating contains only four non-zero scattering elements, there are only two pairs of sub-matrices that support the projected EPs: $\mathbf{S_1}$, $\mathbf{S_2}$, $\mathbf{S_3}$ and $\mathbf{S_4}$. Among these, $\mathbf{S_1}$ and $\mathbf{S_2}$ have already been discussed in the main text. Below, we

will detail how they are obtained through projection operations, and then we will introduce the other two EP-supporting subspaces, $\mathbf{S_3}$ and $\mathbf{S_4}$.

First, we introduce the corresponding input and output projection operators to show the projection process, using $\mathbf{S_1}$ and $\mathbf{S_2}$ as examples. Since the projected subspaces involve asymmetric input-output channel combinations, they are defined by the asymmetric-projection relation as $\mathbf{S_{1,2}} = \mathbf{P}_n^{\mathrm{out}}\mathbf{S_p}\left(\mathbf{P}_n^{\mathrm{in}}\right)^{\mathrm{T}}$ (n=1,2,3,4, indicates the subsystem index), where $\mathbf{P}_n^{\mathrm{in}}$ projects the excitation channels and $\mathbf{P}_n^{\mathrm{out}}$ "selects" the corresponding scattering channels. For $\mathbf{S_1}$ and $\mathbf{S_2}$, $\mathbf{P_1^{in}}$ =[1, 0, 0, 0; 0, 0, 1, 0], $\mathbf{P_1^{out}}$ =[0, 0, 1, 0; 0, 0, 0, 1] and $\mathbf{P_2^{in}}$ =[1, 0, 0, 0; 0, 0, 0, 1], $\mathbf{P_2^{out}}$ =[0, 1, 0, 0; 0, 0, 1, 0]. These asymmetric projection operators provide a clear delineation between the excitation channels and the scattering channels. Physically, $\mathbf{S_1}$ and $\mathbf{S_2}$ represents the input-output behaviors from port 1 (L) to port d (R), and has been discussed in main text. Fig. S4b and S4c show the detailed projection process and input-output behaviors in subspaces of $\mathbf{S_1}$ and $\mathbf{S_2}$.

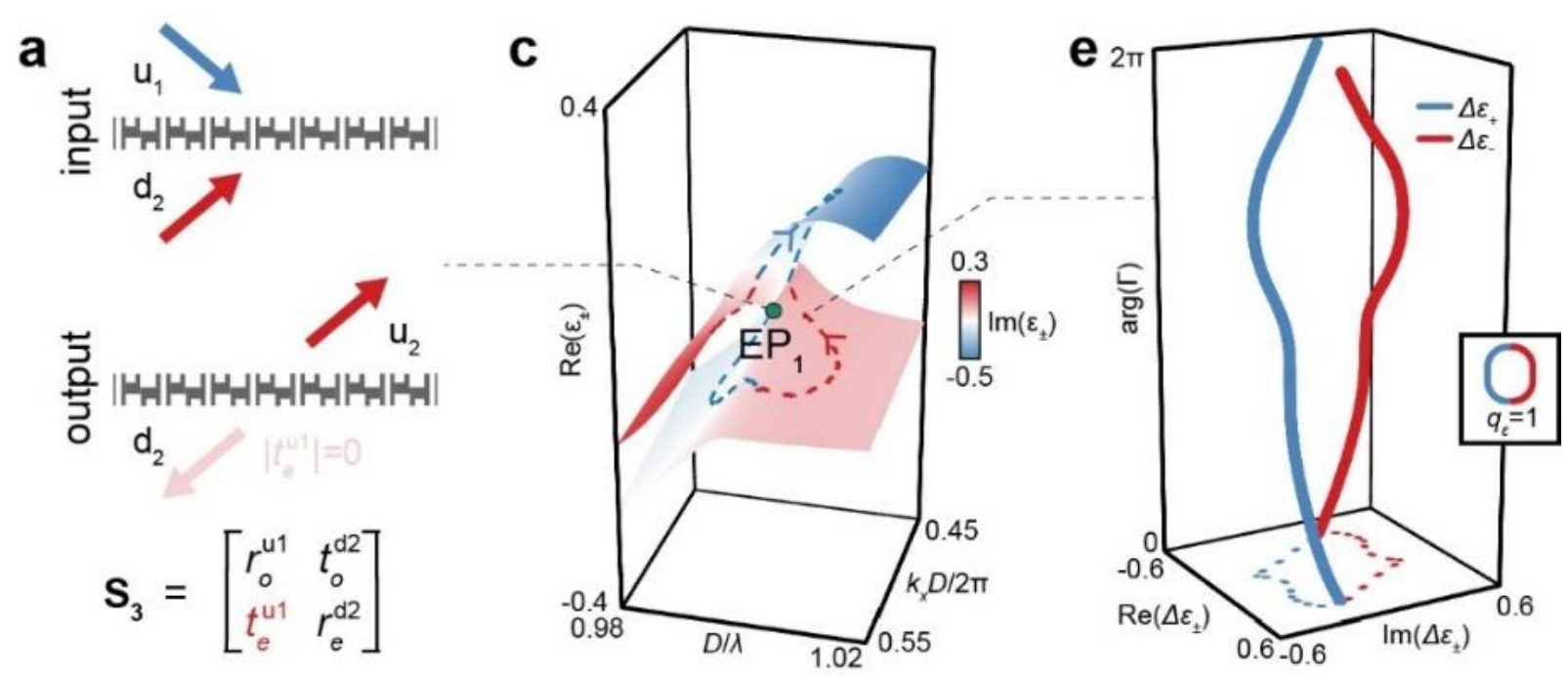

Figure S5 a and **b**, Inter-port scattering behaviors of the two subsystems described by the sub-matrices $\mathbf{S_3}$ and $\mathbf{S_4}$. **c** and **d**, Eigenvalue Riemann surfaces of $\mathbf{S_3}$ and $\mathbf{S_4}$, which exhibit the topological structures of EP_1 (**d**) and EP_2 (**e**), similar with the results of EP_1 and EP_2 shown in Fig. 2(d-e). **e-f**, Adiabatic eigenvalue evolution around EP_1 (**e**) for sub-matrix $\mathbf{S_3}$ and EP_2 (**ff**) for sub-matrix $\mathbf{S_4}$, confirming the two-band charge $q_\epsilon = 1$ and 2, consistent with the results in Fig. 2f and 2h.

Then, we investigate the projected EPs of $\mathbf{S_3}$ and $\mathbf{S_4}$. $\mathbf{S_3}$ reveals the inter-port scattering behaviors from port L to port 2 (shown in Fig. S5a): $\mathbf{b_3} = \mathbf{S_3 a_3}$, where $\mathbf{b_3} = (b_{u2}, b_{d2})^T$, $\mathbf{a_3} = (a_{u1}, a_{d2})^T$ and $\mathbf{S_3} = [r_o^{u1}, t_o^{d2}; t_e^{u1}, r_e^{d2}]$. Similarly, $\mathbf{S_4}$ defines the scattering behaviors from port u to port d (shown in Fig. S5b): $\mathbf{b_4} = \mathbf{S_4 a_4}$, where $\mathbf{b_4} = (b_{d2}, b_{d1})^T$, $\mathbf{a_4} = (a_{u1}, a_{u2})^T$ and $\mathbf{S_4} = [t_e^{u1}, t_o^{u2}; t_o^{u1}, t_e^{u2}]$.

Figures S5c-d illustrate the simulated eigenvalue Riemann surfaces of $\mathbf{S_3}$ and $\mathbf{S_4}$ around the operated point in momentum-frequency domain. The EPs of $\mathbf{S_1}$ (main text, Fig. 2d) and $\mathbf{S_3}$ (Fig. S5d) share the identical branch-cut topologies; both are charge-1 EPs. Likewise, the EPs encoded of $\mathbf{S_2}$ (main text, Fig. 2e) and $\mathbf{S_4}$ (Fig. S5d) share the identical Dirac-like intersecting topologies; both are charge-2 EPs. Notwithstanding that the two Riemann surfaces of the EPs from $\mathbf{S_1}$ and $\mathbf{S_3}$ are not exactly the same due to differences in their entries, they share the same topological nature; and the same holds for $\mathbf{S_2}$ and $\mathbf{S_4}$. Encircling the charge-1 EP and charge-2 EP yield the adiabatic evolution of eigenvalue differences for $\mathbf{S_3}$ and $\mathbf{S_4}$ as shown in Fig. S5e and S5f, consistent with the results of Fig. 2f and 2h in main text. These comparisons between $\mathbf{S_1}$ ($\mathbf{S_2}$) and $\mathbf{S_3}$ ($\mathbf{S_4}$) demonstrate that the topological invariants of these projected EPs are determined by the vanishing element in their sub-matrices and remain robust regardless of the choice of other elements, which will be proved in Supplementary Note 4. Noted that due to the preserved C_2 symmetry, the conclusions obtained by the subsystem with t_e^{u1} (t_o^{u1}) as vanishing element are identical to those for the subsystem t_e^{d1} (t_o^{d1}) as vanishing element. In addition, by appropriately selecting the projected subsystems, it is also possible to generate other degeneracies besides EPs, such as diabolic points (DPs).

Reviewer:

3. While the theoretical derivation provides an intuitive and appealing explanation of this correspondence, the analysis relies on certain approximations (e.g., the local phase-vortex description of the scattering coefficient near the EP and the neglect of anisotropy in the phase distribution). It would be beneficial if the authors could clarify more explicitly the range of validity of this approximation and discuss how robust the EP–STV mapping remains under realistic conditions where anisotropy or spectral distortions may be present.

Response:

Thank you for this professional and constructive comment. We completely agree with your assessment. During our derivation, we intentionally introduced certain necessary simplifications **to render the mathematical formalism more concise and to make the underlying physical picture of the correspondence more intuitive.**

To address your concern, we have explicitly emphasized the approximation conditions and expanded our theoretical analysis with newly added detailed discussions in Supplementary Note 4.

Specifically, in our work, the proof of the EP-STV mapping consists of two primary stages: **1)** The mapping between the eigenvalue structure of EP and the phase structure of scattering coefficients, i.e., $q_\epsilon = q_s$; and **2)** the phase-vortex mapping from the scattering coefficient singularities in momentum-frequency domain to the STV in space-time domain. Both stages employ the narrow-band (local) approximation, which make senses because the exceptional topologies are localized to the vicinity of the singularity (see Ref. 7 in the manuscript). For the second stage (charge mapping), we now explicitly clarify the range of validity: **Eq. S36 (in the Supplementary) holds perfectly under the approximations of a local phase-vortex of scattering coefficients, a circular incident pulse, and a limited integration region**. It should be noted that Eq. S36 has already considered the effect of phase-vortex anisotropy of scattering spectrum, by the mapping of anisotropic parameters A and B, which define the ellipticity of scattering spectrum phase-vortex $\eta_s= (A-B)/(A+B)$. Owing to that the wave structure of scattering STV is determined by both scattering spectrum and the incident pulse, similarly we have defined the ellipticity of the incident pulse envelope spectrum as η_i, generalizing the circular integration region in Eq. S34 to an anisotropic ellipse.

To examine the robustness under realistic anisotropic conditions, we have added a new subsection (named "Robustness of charge mapping" in Supplementary Note 4) to explore the scattering patterns of STV when η_s and η_i are in different relationship. The results obtained by numerical integration reveal that the charge mapping from momentum space to real space remains robust. However, the morphology of the scattering STV is highly sensitive. The anisotropic impact η_i and η_s introduce competing stretching effects on the wave structure of scattering STV pulses, leading to the disintegration of charge-2 STV (as detailed in our response

to the subsequent comment). Furthermore, the scattering coefficient t_e^{u1} and t_o^{u1} used in our work inherently possess significant anisotropy and spectral distortion. **In the subsection "Theoretical prediction of STV generation" (Supplementary Note 4), we demonstrate that even under these perturbations, the charge mapping remains topologically protected.**

Added part in Supplementary Note 4:

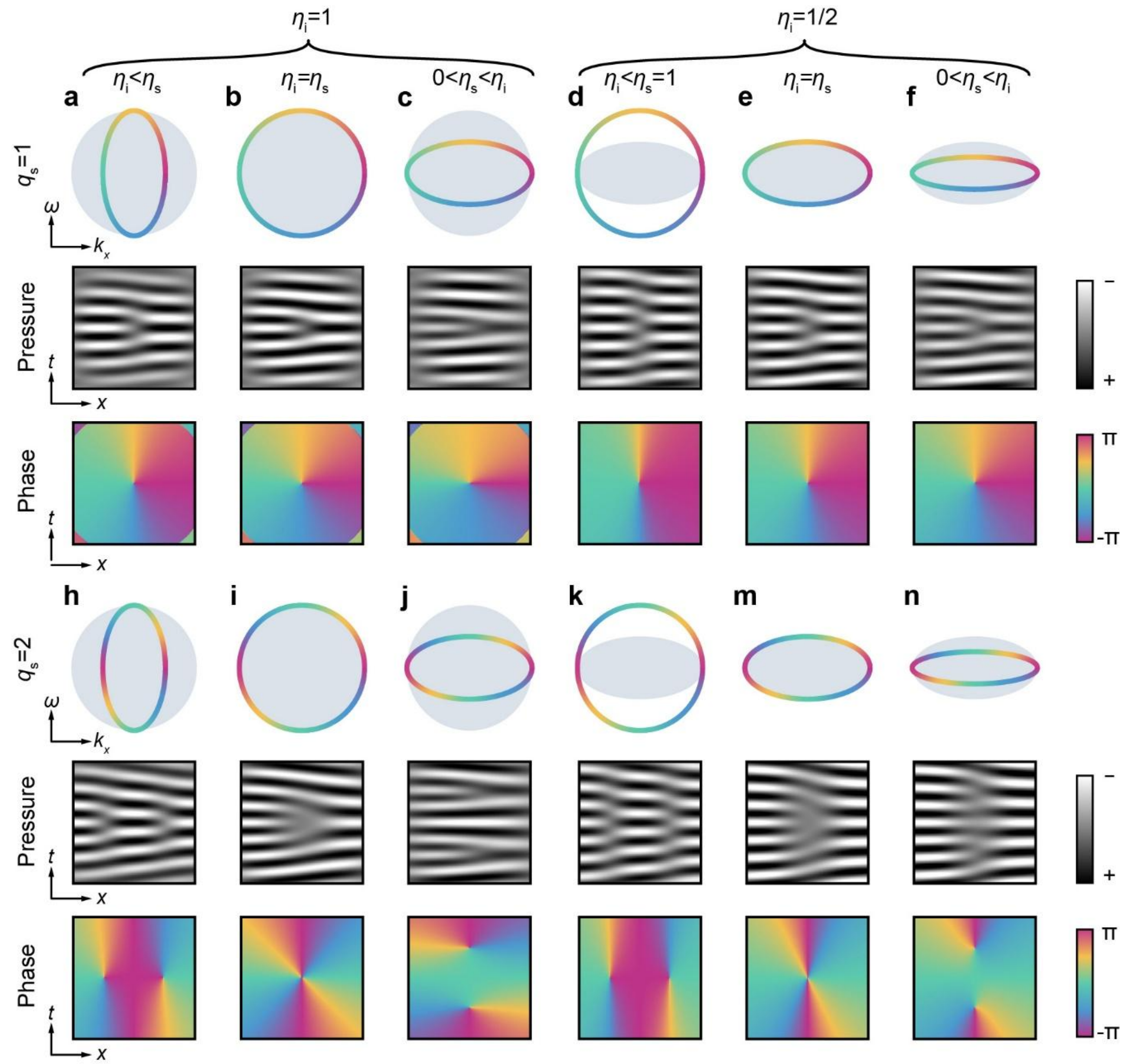

Figure S6 The pressure and phase distribution of charge-1 (q_s =1, **a-f**) and charge-2 (q_s =2, **h-n**) scattering STV in space-time domain, under the different relationship between η_s (the ellipticity of the scattering coefficient vortex, denoted by the colored loop) and η_i (the ellipticity of incident pulse envelope spectrum, denoted by the gray region).

Robustness of charge mapping: Although Eq. S36 provides an analytical solution under specific approximations (e.g., local phase-vortex of scattering coefficients, a circular incident pulse, limited integration region), the wave structure of the scattering STV is determined by the interplay between phase-vortex of the scattering coefficient and the morphology of incident pulse. We define the ellipticity of the incident Gaussian pulse spectrum as η_i, generalizing the circular integration region in Eq. S34 to an anisotropic ellipse. In order to examine the robustness of the charge mapping process, we calculate the numerically integrated pressure and phase distributions of scattering STVs for various relationships between η_i and the ellipticity of scattering coefficient vortex η_s in two cases of q_s =1 and q_s =2, shown in Fig. S6.

The results reveal that η_s and η_i exert two competing stretching effects into the phase distribution of STV and the scattering pulse envelope during the mapping from momentum space to real space, respectively. Moreover, the stretching induced by η_s arises from the mapping of anisotropic parameters A and B in Eq. S36, whereas the inverse-stretching induced by η_i stems from the Fourier transform from the momentum-frequency domain to the space-time domain. Crucially, regardless of these anisotropic distortions, the scattering STVs exhibit a robust and topologically protected charge mapping. For q_s =1, the topological charge of the scattering STV strictly corresponds precisely to that of the phase-vortex of the scattering coefficient. For q_s =2, while the singularity of the scattering STV is unstable (as discussed in the following subsection), the total topological charge remains conserved, further validating the robustness of the charge mapping.

Changes (page 13, lines 8-15) in revised manuscript:

"…Meanwhile, the integral in Eq. 3 governs the radial structure of the envelop, which does not alter the topological charge q_s defined by the phase winding, thereby ensuring the robustness of the spatiotemporal vortex against perturbations in the packet's profile (see robustness analysis of charge mapping in Supplementary Note 4). Note that to render the mathematical formalism concise and to make the underlying physical picture of the correspondence intuitive, the derivation above relies on certain approximations, including the local phase-vortex description of the scattering coefficient near the EP and neglecting the anisotropy in the phase distribution. The former is well-justified since the exceptional topologies are strictly localized to the vicinity of the singularity. For the latter, we have further discussed the scattering patterns of the STVs in the presence of anisotropy (see Supplementary Note 4 for details)."

Changes in Supplementary Note 4 (page 19, lines 1-7, in Supplementary Information):

"The vortex ellipticity $\eta_s = (A\text{-}B)/(A+B)$ represents the anisotropy of the phase distribution. The topological charge of t_e^{u1} is q_s if $\eta_s > 0$, and $-q_s$ if $\eta_s < 0$. when $\eta_s = \pm 1$, the phase of the vortex is an isotropic circular distribution. Noted that we use $\eta_s = 1$ (B=0, $t_e^{\mathrm{u1}}(\rho, \phi) = f(\rho)e^{iq_s\phi}$) as the premise for the derivation in the main text. With the Gaussian profile, the incident pulse has the form $\tilde{a}_{\mathrm{u1}}(k_\perp, \Omega) = \exp\left[-\frac{k_\perp^2}{\Delta k_\perp{}^2} - \frac{\Omega^2}{\Delta\omega^2}\right]$ where $\Delta k_\perp$ and $\Delta\omega$ determine the ellipticity of input pulse envelope. Considering $\Delta\rho = \Delta\omega/c = \Delta k_\perp$, the polar coordinate form is $\tilde{a}_{\mathrm{u1}}(\rho) = \exp\left(-\frac{\rho^2}{\Delta\rho^2}\right)$, which lacks phase variation."

Changes in Supplementary Note 4 (page 24, lines 2-6, in Supplementary Information):

"…In Fig. S6, the width of Gaussian incident pulse envelope spectrum, both Δk_x and Δk are 0.05G ($\Delta\omega = c\Delta k$), which means $\eta_i = 1$. It is found that the phase-vortex of t_e^{u1} and t_o^{u1} exhibits significant spectral distortions and are compressed in ω-axis ($0<\eta_s<1$). Based on the conclusions of previous section, since the incident pulse envelope does not match the anisotropic phase-vortex of t_e^{u1} and t_o^{u1} ($\eta_s < \eta_i$), the vortex-wave structure will be stretched in propagation direction.…"

Reviewer:

4. Regarding the generation of a charge-2 STV, the authors could elaborate on whether the observed structure should be interpreted as a genuine higher-order STV that subsequently splits, or simply as two closely related first-order vortices.

Response:

This is an excellent question. This inquiry touches upon a highly important phenomenon in topological physics and singular optics: **the structural instability of higher-order singularities**. **In wave physics, while higher-order singularities (topological charge q≥2) exist mathematically, they are inherently structurally unstable in physical systems.** This implies that any minute physical perturbation—such as slight structural asymmetries, non-ideal material dispersion, or even grid discretization errors in numerical simulations—breaks the strict symmetry

required to maintain the degeneracy, causing the higher-order singularity to unfold or split into multiple first-order singularities. As demonstrated in important works such as Ricci et al. (Opt. Express, 20(20), 22961, 2012) and Soskin & Vasnetsov (Prog. Opt., 42, 219, 2001), **higher-order optical vortices possess intrinsic instability**. Non-ideal anisotropy or minor perturbations inevitably cause these higher-order singularities to disintegrate into a series of unit-charge vortices, although the global topological charge remains strictly conserved. **This fundamental principle holds equally true for our STV**. Therefore, the observed structure should be interpreted as a genuine higher-order STV that subsequently splits, rather than coincidental first-order vortices.

Specifically, regarding our spatiotemporal vortices, the challenges in observing a stable higher-charge STV arise from two main factors: **1)** The perturbation to the higher-charge scattering STVs, caused by the mismatched shape of the scattering spectrum phase-vortex. **2)** The disintegration of the higher-charge STV during propagation. Following the discussion regarding the previous comment, **we have added a new subsection ("Disintegration of charge-2 STV") and a new figure (Fig. S5) in Supplementary Note 4 to explain the influence of the first factor on the charge-2 STV.** The second factor corresponds to the phase velocity differences of the plane-wave components with different frequencies in the scattering STV during propagation; this is due to the medium's dispersion relationship and has already been discussed extensively in previous literature (see Refs. 39 and 50 in the manuscript). According to the newly added section, we clarify that **the scattering STV originating from EP_2 should be interpreted as a disturbed charge-2 STV that subsequently splits into two closely related charge-1 STVs**, specifically considering the mismatch between the incident pulse and the phase-vortex of the scattering spectrum as an inherent perturbation.

At the parameter position corresponding to EP_2, the scattering coefficient t_o^{u1} possesses a phase-winding charge of $q_s = 2$. Thus, the initial imprint onto the scattering wave packet is indeed a true singular charge-2 vortex governed by the EP-STV correspondence. However, as elaborated in our newly added "Disintegration of charge-2 STV" section, higher-charge spatiotemporal singularities are fundamentally unstable in scattering dynamics. Under realistic experimental conditions, it is practically unattainable for the incident pulse morphology (η_i) to perfectly match the anisotropic scattering coefficient vortex (η_s). This mismatch manifests as competing stretching effects on the phase-vortex and the envelope of the scattering STV pulse, acting as an unavoidable perturbation that distorts the scattering wave structure. Consequently, the genuine charge-2 singularity cannot

maintain its single-point structural integrity and immediately disintegrates along the spatial or temporal dimension into two stable charge-1 STVs. Nevertheless, this does not indicate a failure of the charge mapping; the global topological charge of the disintegrated charge-2 STV remains strictly conserved (1 + 1 = 2). **To accurately reflect this physical reality, we have deliberately used the description "charge-2" rather than '2nd-order' throughout this work.** This choice of terminology emphasizes the conserved topological invariant while actively acknowledging the inherent physical instability of the singular point's internal structure.

Added part in Supplementary Note 4:

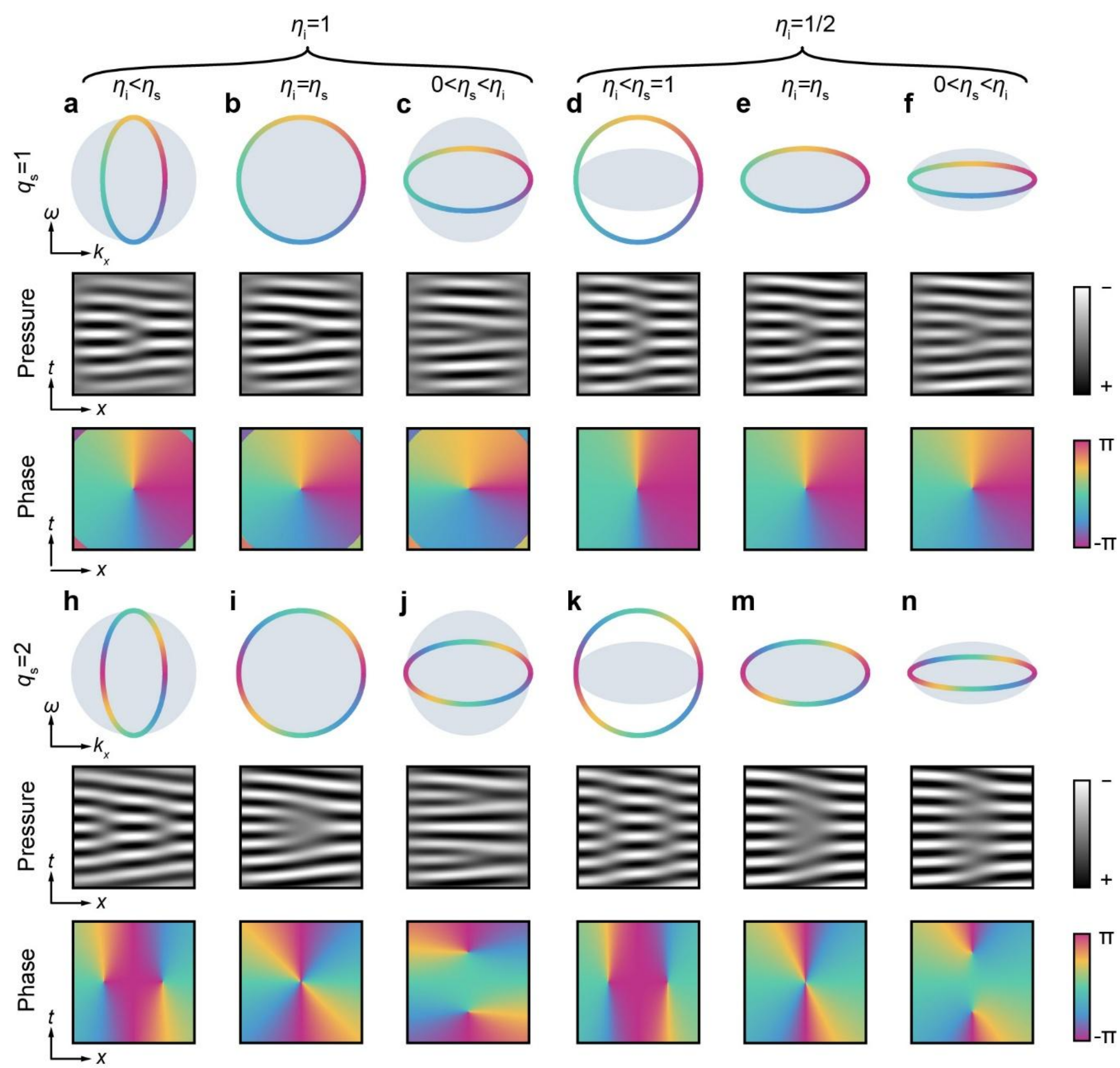

Figure S6 The pressure and phase distribution of charge-1 (q_s =1, **a-f**) and charge-2 (q_s =2, **h-n**) scattering STV in space-time domain, under the different relationship between η_s (the

ellipticity of the scattering coefficient vortex, denoted by the colored loop) and η_i (the ellipticity of incident pulse envelope spectrum, denoted by the gray region).

Disintegration of charge-2 STV: While Eq. S36 guarantees a strict mapping of topological charges between phase-vortex of scattering coefficients and STVs, it does not ensure the stability of higher-charge singularity. Specifically, a charge-2 STV ($q_s = 2$) is highly prone to split into two charge-1 elementary STVs, primarily due to the mismatch between the scattering spectrum (η_s) and incident pulse spectrum (η_i). This instability is investigated in Fig. S6h-n, which display how the competing stretch leveraged by η_i and η_s affects the wave structure of charge-2 STV.

When η_i =1, which means that the shape of incident pulse is a standard circle, we display the scattering patterns of the STV for the three cases where $\eta_i < \eta_s$ (Fig. S6a), $\eta_i = \eta_s$ (Fig. S6b) and $1< \eta_s < \eta_i$ (Fig. S6c). It is obvious that when $\eta_i < \eta_s$ or $0 < \eta_s < \eta_i$, the charge-2 STV splits into two charge-1 singularities along the space (x) or time (t) dimension. Only when $\eta_i = \eta_s$ does the charge-2 STV not split, retaining an individual charge-2 singularity. In Fig. S6d-f, we set η_i = 1/2, under the conditions of $\eta_i < \eta_s = 1$ (Fig. S6d), $\eta_i = \eta_s$ (Fig. S6e) and $0 < \eta_s < \eta_i$ (Fig.S6f), we obtain the same conclusions as Fig. S6a-c. Consequently, the charge-2 STV remains stable only when the opposing stretching effects are perfectly matched, i.e., $\eta_i = \eta_s$. However, the perfect shapes of incident pulse and scattering spectrum vortex are virtually impossible in practical applications. It should be noted, that medium dispersion—which causes phase velocity inconsistencies among various plane-wave components—serves as an additional factor contributing to wave packet disintegration during propagation.

Changes (page 13, lines 22-23 and page 14, lines 1-2) in revised manuscript:

"…Notably, driven by the inherent structural instability of higher-order singularities under unavoidable physical perturbations[38, 39], the genuine charge-2 STV generated by EP_2 inevitably splits into two stable charge-1 STVs while strictly conserving the global topological charge (see details in Supplementary Note 4).…"

Reviewer:

5. The experimental confirmation of the topological charge appears somewhat qualitative (although visually convincing). Can the authors provide a quantitative extraction of topological charge from the experimental phase maps, rather than just visual phase behaviors?

Response:

Thank you for promptly pointing out this critical aspect. Initially, we chose the water surface wave platform precisely **to provide a highly intuitive and direct "visualization" of the STVs**. However, in doing so, we admittedly overlooked the necessity of providing rigorous quantitative data to support these visual observations. We are sorry for this.

Following your advice, we have re-analyzed our experimental data to quantitatively extract the topological charges. Specifically, we performed a discrete closed-loop integral of the phase gradient around the scattering pulse centers. **This new analysis has now been incorporated as an added section in Supplementary Note 5**. The experimentally extracted topological invariant is determined by the formula: $q_{\mathrm{exp}} = (\frac{1}{2\pi}) \oint_C d[\arg(b)]$, where *b* is the complex envelope of the scattering pulse, and *C* represents a closed loop encircling the phase singularity.

Importantly, by calculating the accumulated phase along closed loops enclosing the scattering pulse centers in **both the spatial section (*x*-*z* domain at a constant *t*) and the spatiotemporal section (*x*-*t* domain at a constant *z*), we unambiguously demonstrated a unique topological feature: the STV carries the identical topological charge in both the purely spatial and the mixed spatiotemporal domains**.

As shown in our newly added quantitative results: For the anomalous-transmitted STV (generated by EP1), the values of the extracted topological charges, in both the spatial and spatiotemporal domains, are exactly close to the ideal value of $q_1^{\mathrm{exp}} = 1$. For the specular-transmitted case (generated by EP2), by integrating along a wider loop that encloses both of the split singularities, the phase accumulation yields a total topological charge of $q_2^{\mathrm{exp}} = 2$. This quantitative extraction not only strongly solidifies our experimental claims beyond visual phase behaviors but also perfectly corroborates the total charge conservation during the mapping from static exceptional topologies to dynamic STVs.

Added part in Supplementary Note 5:

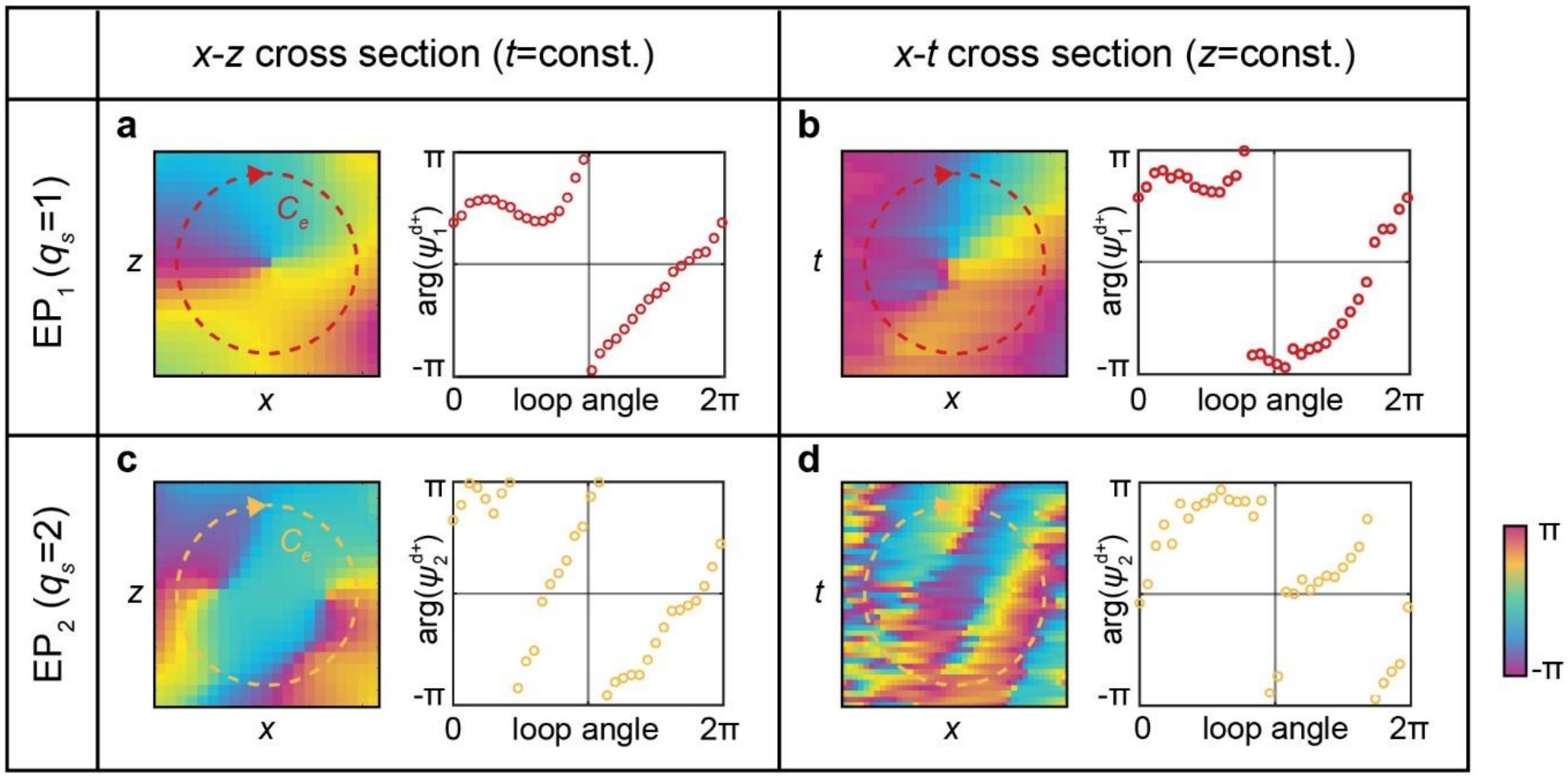

Figure S11 Topological charge q^{exp} extracted from the transmission phase pattern in spatial (x-z, t=const.) cross section (**a** and **c**) and space-time (x-t, z=const.) cross section (**b** and **d**).

Topological charge of STVs extracted from experimental results: In view of the experimental results, we can extract the topological charges of STVs (q^{exp}) from phase patterns of scattering pulse envelopes. For anomalous-transmitted pulse corresponding to EP_1, $q_1^{\mathrm{exp}} = (1/2\pi)\oint_C d\arg(b_{\mathrm{d1}})$, and for specular-transmitted pulse corresponding to EP_2, $q_2^{\mathrm{exp}} = (1/2\pi)\oint_C d\arg(b_{\mathrm{d2}})$, where C is a closed integral loop encircling the pulse center shown in Fig. S11. In Fig. S11, we exhibit the distribution of $\arg(b_{\mathrm{d1}})$ and $\arg(b_{\mathrm{d2}})$ in spatial section (x-z plane with t=const., the data from Fig. 4i) and space-time section (x-t plane with z=const., the data from Fig. S10b), as well as the phase variation along the integral loop. It is evident that whether in x-z or x-t cross section, b_{d1} carries one charge-1 singularity and b_{d2} carries two charge-1 singularities. Considering one encircling cycle along C, the phase $\arg(b_{\mathrm{d1}})$ undergoes a 2π-change, while $\arg(b_{\mathrm{d2}})$ undergoes a 4π-change, which indicates that the topological charge of b_{d1} is $q_1^{\mathrm{exp}} = 1$ and the total topological charge of b_{d2} is $q_2^{\mathrm{exp}} = 2$. The results from the experimental analysis are consistent with our theory and simulations, confirming the EP-STV correspondence in our work.

Changes (page 17, lines 5-8) in revised manuscript:

"We also extract the topological charge of scattering pulses from the experimental results, quantitatively demonstrating the topological features of the STVs in both the purely spatial and the mixed spatiotemporal domains (see Supplementary Note 6 for details)."

Reviewer:

Minor comments:

1. A few long sentences in the Introduction could be slightly shortened to improve flow.
2. There might be some typos (e.g., 'metrix' on page 11).
3. In Fig. 3, it would be helpful to include color bars for clarity.

Response:

Thanks for these comments. All of the mentioned minor issues have been carefully addressed and corrected in the revised manuscript.

Revised Figure 3:

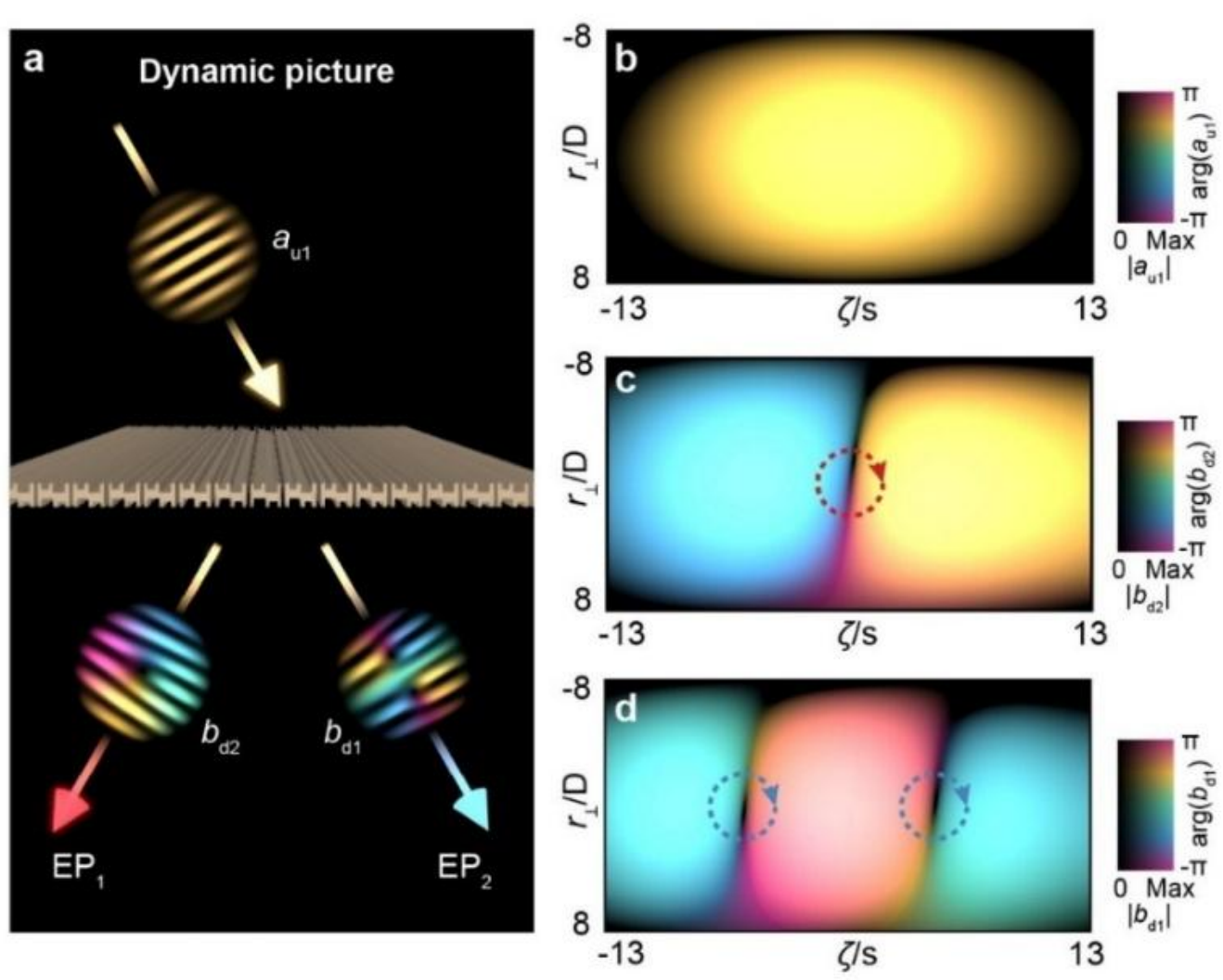

Once again, we sincerely thank you for your meticulous review. Your precise and expert comments have substantially elevated the overall quality and rigor of our manuscript. We deeply appreciate your expert guidance.

Response to Referee 2 -- NCOMMS-26-011336-T

Reviewer:

The authors study scattering exceptional points (S-EPs) with their dynamic manifestations in the form of spatiotemporal vortices (STVs). They establish a clear EP–STV correspondence and provide compelling experimental evidence using a liquid-surface-wave platform. The work is timely, technically sound, and of broad interest to the spatiotemporal physics, wave dynamics, and metamaterials communities. The manuscript is generally well written and the results are convincing. However, several points require clarification or further elaboration before the paper can be considered for publication

Response:

Thank you for the positive evaluation of our work and the constructive comments. We are encouraged by the comment "timely, technically sound, and of broad interest to the communities of spatiotemporal physics, wave dynamics, and metamaterials". Below, we provide a point-by-point response to your concerns, and we have revised the manuscript accordingly.

Reviewer:

1. The current formulation of Eq. (1) makes it difficult for readers to clearly interpret how the 4-port scattering matrix is partitioned into the two 2×2 subsystems S1 and S2. Since these two subsystems are central to the emergence of the distinct EPs (charge-1 and charge-2), I strongly suggest that the authors explicitly define S1 and S2 in the main text, rather than relying on readers to infer them from Eq. (1).

Response:

Thank you for pointing out this. In fact, this was a real "headache" for us when drafting the manuscript: To achieve projected EPs, the projection of the original scattering system (described by the full 4×4 matrix $\mathbf{S_p}$) to a lower-dimensional subsystem with a projected 2×2 submatrix can

take various forms. Except for a few intuitive cases (such as considering only the upper half-space as a 2×2 subsystem), **the vast majority of projection transformations are abstract** (like our $\mathbf{S}_1$ and $\mathbf{S}_2$ here), which indeed poses significant difficulties for readability.

Now your comment points out that we did not address this issue well in the original manuscript. You are right, since that $\mathbf{S}_1$ and $\mathbf{S}_2$ are the fundamental cornerstones for the emergence of the distinct charge-1 and charge-2 projected EPs, relying on readers to mentally reconstruct them from Eq. (1) creates an unnecessary barrier to understanding. To resolve this issue and make the definition of $\mathbf{S}_1$ and $\mathbf{S}_2$ as intuitive and explicit as possible, we have adopted a comprehensive approach in our revised manuscript:

1. Mathematical

Following Eq. (1) in the revised main text, we now explicitly write out the exact matrix formulations of $\mathbf{S}_1$ and $\mathbf{S}_2$. We show exactly how these submatrices are extracted from the full 4×4 matrix $\mathbf{S}_\mathbf{p}$, we have introduced the corresponding input and output mathematical projection operators [$\mathbf{S}_1$—Eq. (R1); $\mathbf{S}_2$—Eq. (R2)]. We hope this can provide a clear delineation between the excitation channels and the observation/scattering channels.

$$\begin{bmatrix} b_{u1} \\ b_{u2} \\ b_{d1} \\ b_{d2} \end{bmatrix} = \mathbf{S}_\mathbf{p}(k_x,\omega) \begin{bmatrix} a_{u1} \\ a_{u2} \\ a_{d1} \\ a_{d2} \end{bmatrix} = \begin{bmatrix} r_e^{\mathrm{u1}} & r_o^{\mathrm{u2}} & t_o^{\mathrm{d1}} & t_e^{\mathrm{d2}} \\ r_o^{\mathrm{u1}} & r_e^{\mathrm{u2}} & t_e^{\mathrm{d1}} & t_o^{\mathrm{d2}} \\ t_o^{\mathrm{u1}} & t_e^{\mathrm{u2}} & r_e^{\mathrm{d1}} & r_o^{\mathrm{d2}} \\ t_e^{\mathrm{u1}} & t_o^{\mathrm{u2}} & r_o^{\mathrm{d1}} & r_e^{\mathrm{d2}} \end{bmatrix} \begin{bmatrix} a_{u1} \\ a_{u2} \\ a_{d1} \\ a_{d2} \end{bmatrix}, \tag{R1}$$

$$\begin{bmatrix} b_{u1} \\ b_{u2} \\ b_{d1} \\ b_{d2} \end{bmatrix} = \mathbf{S}_\mathbf{p}(k_x,\omega) \begin{bmatrix} a_{u1} \\ a_{u2} \\ a_{d1} \\ a_{d2} \end{bmatrix} = \begin{bmatrix} r_e^{\mathrm{u1}} & r_o^{\mathrm{u2}} & t_o^{\mathrm{d1}} & t_e^{\mathrm{d2}} \\ r_o^{\mathrm{u1}} & r_e^{\mathrm{u2}} & t_e^{\mathrm{d1}} & t_o^{\mathrm{d2}} \\ t_o^{\mathrm{u1}} & t_e^{\mathrm{u2}} & r_e^{\mathrm{d1}} & r_o^{\mathrm{d2}} \\ t_e^{\mathrm{u1}} & t_o^{\mathrm{u2}} & r_o^{\mathrm{d1}} & r_e^{\mathrm{d2}} \end{bmatrix} \begin{bmatrix} a_{u1} \\ a_{u2} \\ a_{d1} \\ a_{d2} \end{bmatrix}, \tag{R2}$$

2. Textual

Textually, we have added detailed physical descriptions to clarify what these abstract submatrices actually represent in real space. **Specifically, we explicitly state that $\mathbf{S}_1$ describes the scattering from the "diagonal" half-space (i.e., top-left and bottom-right ports) to the lower half-space. Similarly, $\mathbf{S}_2$ maps the inputs from the left half-space to the outputs in the right half-space.** This provides a clear physical picture of the chosen sub-spaces. <u>And the reason we chose these two subsystems is that these specific channel combinations directly correspond to the excitation</u>

configurations for observing anomalous transmission/reflection encoding projected EPs in our experiments.

3. Visual

To provide an immediate and intuitive visual guide, we added dedicated schematic illustrations that explicitly highlight the specific input and output channels corresponding to $\mathbf{S_1}$ and $\mathbf{S_2}$, respectively (Fig. R1). By isolating these active ports visually, readers can bridge the mathematical submatrices with the physical geometric configurations.

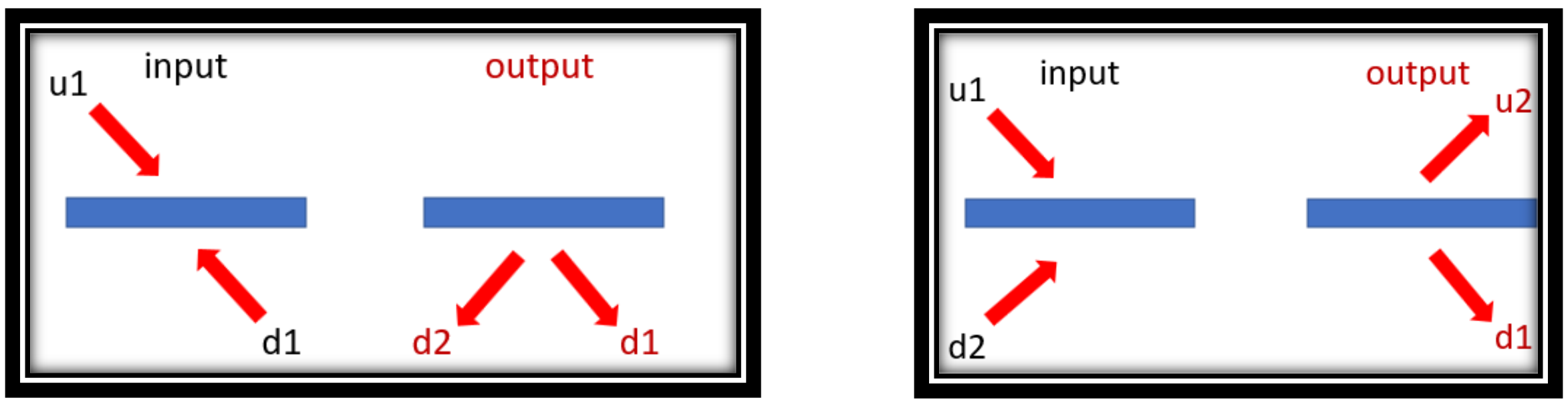

Fig. R1. Schematics of the input-output relationships of subspaces of $\mathbf{S_1}$ (left) and $\mathbf{S_2}$ (right)

According to your comment, we have added a new section ("Constructing rule of projected EP") as the Supplementary Note 3, in which we divide the four-port scattering system into different 2-dimensional subspaces. We also revised the text below Eq. (1) on Page 8 and exhibited the detailed projection process in Fig. S4. We believe that these combined modifications help in eliminating the abstraction and will improve the readability of the theoretical framework.

Changes (page 8, lines 3-8) in revised manuscript:

"⋯To reveal these hidden degeneracies, we project the full scattering matrix onto two distinct subsystems. The first, characterized by the block $\mathbf{S_1}$ $(\boldsymbol{k_x}, \boldsymbol{\omega}) = [t_o^{u1}, r_e^{d1}; t_e^{u1}, r_o^{d1}]$, describes the scattering from the "diagonal" space (e.g. ports u_1 and d_1) to the lower-half plane (e.g. ports d_1 and d_2), as illustrated in Fig. 2b. In this subspace, the scattering behavior is described by $\mathbf{b_1} = \mathbf{S_1 a_1}$, where $\mathbf{b_1} = (b_{d1}, b_{d2})^T$, $\mathbf{a_1} = (a_{u1}, a_{d1})^T$. The second one, given by the block $\mathbf{S_2}$ $(\boldsymbol{k_x}, \boldsymbol{\omega}) = [r_o^{u1}, t_o^{d2}; t_o^{u1}, r_o^{d2}]$ maps inputs from the left half-space to outputs in the right half-space, i.e., $\mathbf{b_2} = \mathbf{S_2 a_2}$, where $\mathbf{b_2} = (b_{u2}, b_{d1})^T$, and $\mathbf{a_2} = (a_{u1}, a_{d2})^T$. ⋯"

Added part in Supplementary Information as Supplementary Note 3:

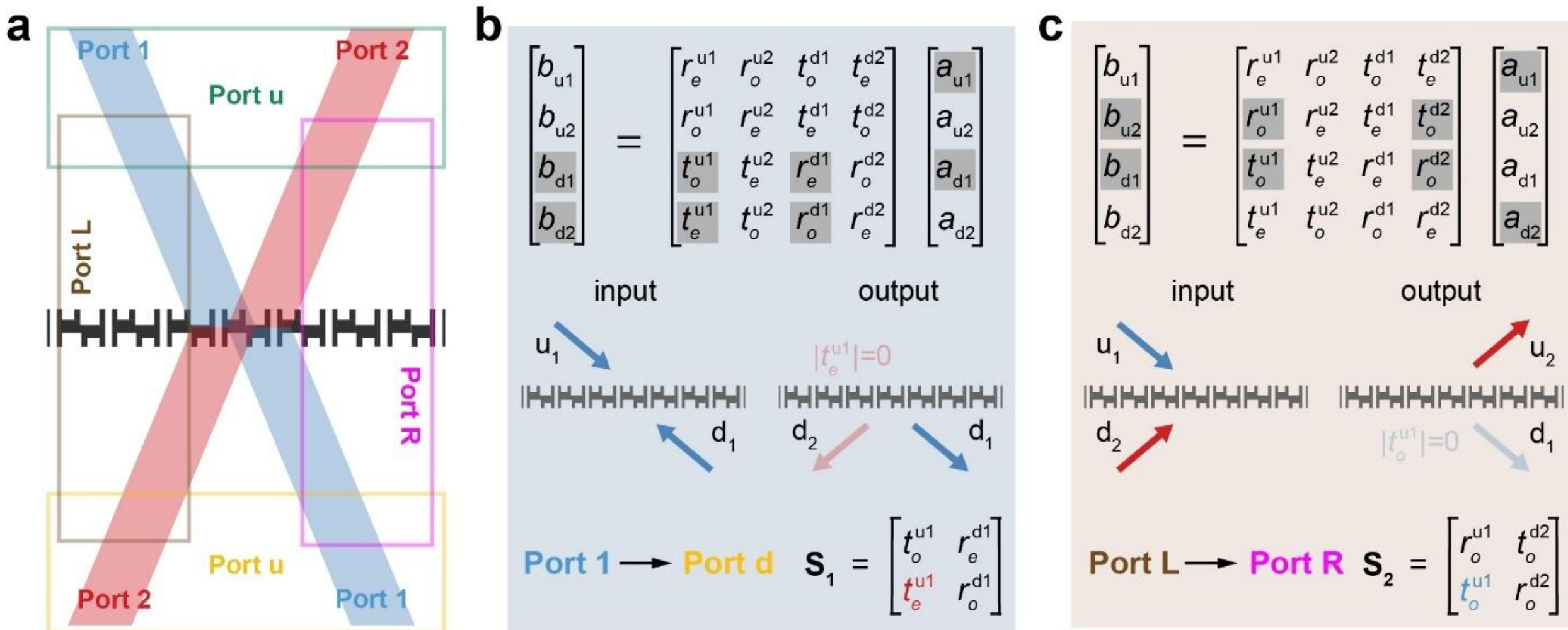

Figure S4 **a**, The full space is divided into six 2-dimensional subspaces by the intrinsic symmetry of the metagrating. Specifically, ports u and d (vertical half spaces, colored by green and yellow), ports 1 and 2 (diagonal half-spaces, colored by blue and red), ports L and R (horizontal half spaces, colored by brown and magenta). **b** and **c**, Detailed projection process and input-output behaviors of $\mathbf{S_1}$ and $\mathbf{S_2}$ mentioned in the main text.

For the designed metagrating system shown in the main text, the element-wise absolute value of its full scattering matrix takes the form abs($\mathbf{S_p}$) = [1, 0, 0, 0; 0, 0, 0, 1; 0, 0, 1, 0; 0, 1, 0, 0]. Here we divide the full space with six 2-dimensional subspaces by the intrinsic symmetry of the metagrating, shown by Fig. S4a. To be specific, we divide the full 4-dimentional space into paired subspaces, including the diagonal and anti-diagonal half-spaces defined by C_2 rotation symmetry as port 1 and port 2, the upper and lower half-spaces defined by up-down mirror symmetry as port u and port d, and the left and right half-spaces defined by left-right mirror symmetry as port L and port R. In principle, by selecting 2 out of the 4 input ports and 2 out of the 4 output ports, there are in total 36 possible 2×2 sub-matrices (i.e. 36 2-dimentional subspaces). However, due to the strict mathematical requirement for the emergence of an EP (i.e., forming a non-diagonalizable Jordan block), the number of subsystems capable of hosting a projected EP is far fewer than 36. Mathematically, a second-order Jordan block requires that only one of its off-diagonal elements vanishes. Given that the scattering matrix $\mathbf{S_p}$ of our designed metagrating contains only four non-zero scattering elements, there are only two pairs of sub-matrices that support the projected EPs: $\mathbf{S_1}$, $\mathbf{S_2}$, $\mathbf{S_3}$ and $\mathbf{S_4}$. Among these, $\mathbf{S_1}$ and $\mathbf{S_2}$ have already been discussed in the main text. Below, we will detail how they are obtained through projection operations, and then we will introduce the other two EP-supporting subspaces, $\mathbf{S_3}$ and $\mathbf{S_4}$.

First, we introduce the corresponding input and output projection operators to show the projection process, using $\mathbf{S_1}$ and $\mathbf{S_2}$ as examples. Since the projected subspaces involve asymmetric input-output channel combinations, they are defined by the asymmetric-projection relation as $\mathbf{S}_{1,2} = \mathbf{P}_n^{\text{out}}\mathbf{S}_\mathbf{p}\left(\mathbf{P}_n^{\text{in}}\right)^{\text{T}}$ (n=1,2,3,4, indicates the subsystem index), where $\mathbf{P}_n^{\text{in}}$ projects the excitation channels and $\mathbf{P}_n^{\text{out}}$ "selects" the corresponding scattering channels. For $\mathbf{S_1}$ and $\mathbf{S_2}$, $\mathbf{P_1^{in}}$ =[1, 0, 0, 0; 0, 0, 1, 0], $\mathbf{P_1^{out}}$ =[0, 0, 1, 0; 0, 0, 0, 1] and $\mathbf{P_2^{in}}$ =[1, 0, 0, 0; 0, 0, 0, 1], $\mathbf{P_2^{out}}$ =[0, 1, 0, 0; 0, 0, 1, 0]. These asymmetric projection operators provide a clear delineation between the excitation channels and the scattering channels. Physically, $\mathbf{S_1}$ and $\mathbf{S_2}$ represents the input-output behaviors from port 1 (L) to port d (R), and has been discussed in main text. Fig. S4b and S4c show the detailed projection process and input-output behaviors in subspaces of $\mathbf{S_1}$ and $\mathbf{S_2}$.

Reviewer:

2. The manuscript states that the metagrating preserves C2 rotational symmetry, but the axis of this C2 rotation is never explicitly stated. This is important because the projection of the full 4-port scattering matrix into two decoupled 2×2 subspaces (S1 and S2) and the existence of two independent EPs both rely critically on this symmetry. The authors should explain how this symmetry maps the four ports into two symmetric pairs, and why this guarantees that S1 and S2 remain decoupled. Does slight symmetry breaking would couple the two subsystems or modify the EP structure?

Response:

Thank you for raising this important issue. We apologize for not explicitly defining the axis of the C_2 rotation in our original manuscript, which may have led to some ambiguity regarding the subsystem decomposition. We completely agree that this symmetry is crucial to our system. Below, we detail our response to address your queries regarding the symmetry axis, the subsystem relations, and the effects of symmetry breaking:

1. The Axis of C_2 Rotational Symmetry and Port Mapping

In our zigzag metagrating system, **the axis of the C_2 rotational symmetry is the out-of-plane axis (i.e., the *y*-axis, corresponding to C_{2y} symmetry)**, which passes through the incident *x*-*z* plane (as marked in the coordinates of Fig. 2a of the manuscript). Under this 180° rotation, the

spatial coordinates transform as (x, y, z) to ($-x$, y, $-z$), effectively mapping the top surface structure perfectly onto the bottom surface structure. Consequently, this operation maps the input/output ports in a crossed manner: Port u_1 (top-left) is mapped to Port d_1 (bottom-right), and Port u_2 (top-right) is mapped to Port d_2 (bottom-left). **Thus, the C_2 symmetry rigorously maps the four ports into two symmetric top-bottom pairs.**

2. The Shared-Port Nature of S_1 and S_2 and the Role of Symmetry

Regarding the decoupling of the subsystems, we realize our original text did not convey our design clearly. **The two 2×2 projected subspaces (S_1 and S_2) are actually not mutually decoupled complementary spaces**. On the contrary, **S_1** and **S_2** deliberately share a common input channel (the top-left port, u_1). As established in our theoretical framework, the exceptional topology of EP_1 and EP_2 is encoded in their respective vanishing matrix elements, t_e^{u1} and t_o^{u1}. These elements scatter waves **from the same shared input channel (u_1) to entirely different output channels:** t_e^{u1} routes to the bottom-left port (d_2), while t_o^{u1} routes to the bottom-right port (d_1). This specific design spatially separates the transmitted fields into distinct left and right sections. This enables the independent and simultaneous **observation of topological signatures from different EPs under a single wave-packet incidence, without any spatial overlap**. Therefore, rather than guaranteeing that **S_1** and **S_2** remain completely decoupled, the C_2 symmetry strictly constrains the inter-system coupling between these overlapping scattering channels.

3. The Effect of C_2 Symmetry Breaking

Your question "Does slight symmetry breaking would couple the two subsystems or modify the EP structure?" points to a critical mechanism in our system that we had not previously recognized: When a slight C_2-breaking perturbation (denoted as Δw_2 in our work) is introduced, the symmetry-protected coupling constraints are altered, **leading to a fundamental change in the EP topology**. As illustrated in our newly-added eigenvalue sensitivity analysis (Fig. S13), once the C_2 symmetry is broken ($\Delta w_2 \neq 0$), **the charge-2 EP_2 immediately splits into two fundamental charge-1 EPs, implying its structurally unstable.** This highlights the extreme sensitivity of the higher-order EP to the C_2 symmetry, meaning that even a slight symmetry-breaking perturbation results in its unfolding into two closely spaced charge-1 EPs (although the total topological charge remains strictly conserved).

In response to your comments, we have revised the main text to explicitly define the C_{2y} axis and clarify the shared-port mechanism between $\mathbf{S_1}$ and $\mathbf{S_2}$. Additionally, we have revised the subsection "Evolution of projected EPs governed by C_2-preserving and C_2-breaking perturbations" and Figure S13 in Supplementary Note 7 to thoroughly explain the C_2-protected EP topological robustness.

Added part in Supplementary Information as Supplementary Note 6:

Evolution of projected EPs governed by C_2-preserving and C_2-breaking perturbations

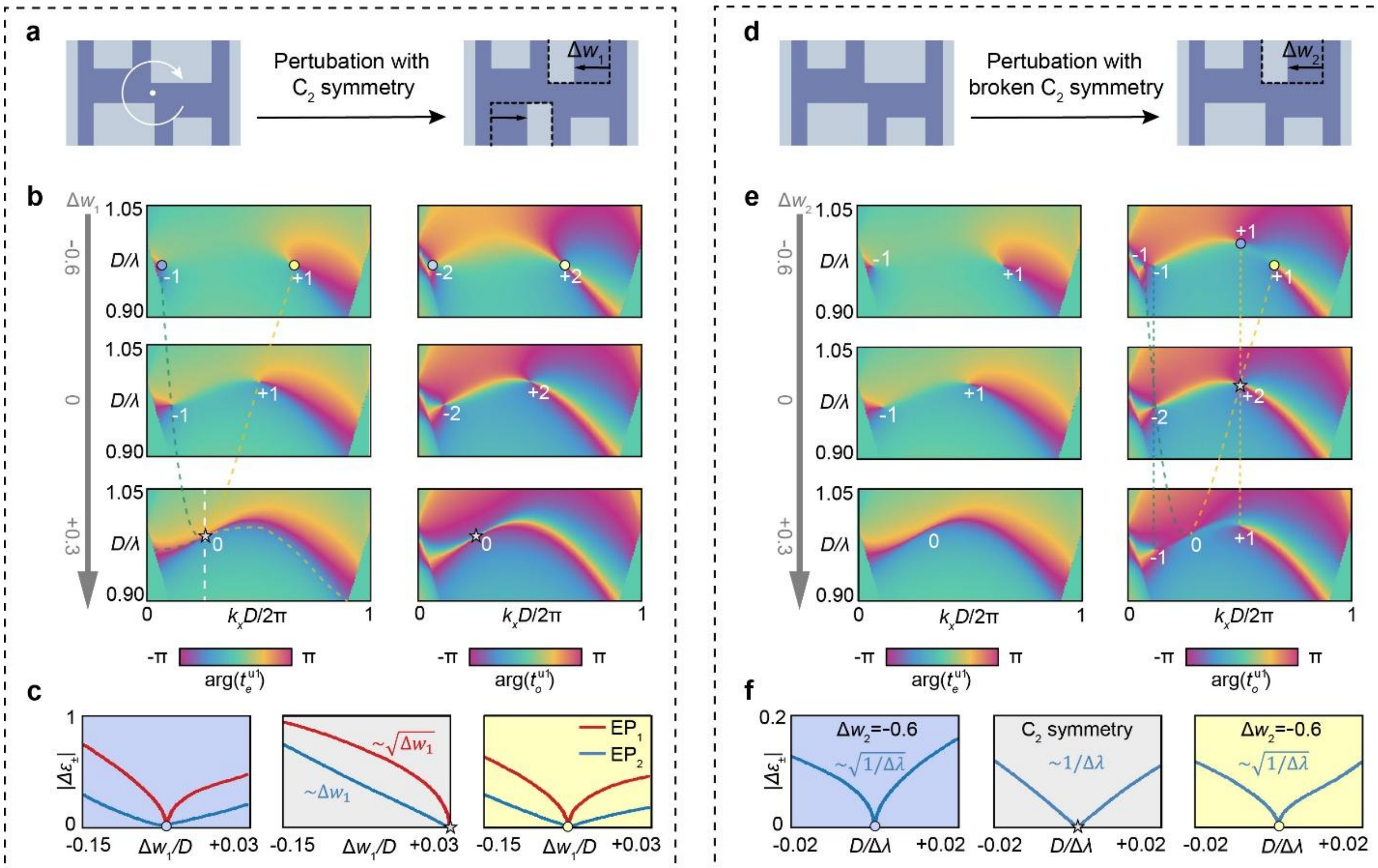

Figure S13 a, Schematic of the C_2-preserving geometric perturbation Δw_1 introduced to the original metagrating. **b**, Evolution trajectories of the scattering spectral singularities (t_e^{u1} and t_o^{u1}) in momentum–frequency domain with varied Δw_1. The green and yellow dashed lines represent EAs with opposite eigen-winding charges undergoing a merging process, during which the locations of EP_1 and EP_2 are locked together under the constraint of C_2 symmetry. The purple and yellow circles denote pre-merge points with opposite charges, and the gray star indicates the merging point. **c**, Eigenvalue sensitivity variation along Δw_1 for the three representative points marked in **b**. EP_1 and EP_2 exhibit square-root (charge-1) and linear (charge-2) sensitivities, respectively. **d**, Schematic of the C_2-breaking perturbation Δw_2 for comparison. **e**, Evolution of spectral singularities with varied Δw_2. The broken C_2 symmetry causes EP_2 to split into two EPs with identical topological charges, destroying the location-anchoring between EP_1 and EP_2. **f**, Eigenvalue sensitivity as a function of wavenumber k for the three points marked in **e**. The split EPs possess square-root sensitivities before merging, which transitions to a linear sensitivity at the merging point (Δw_2=0), where C_2 symmetry is restored.

In a lossless and passive four-port metagrating, the projected subsystems are inherently coupled due to scattering-matrix unitarity. The structural symmetry tightly influences and modulates this inter-system coupling. The zigzag metagrating discussed in the main text breaks both left-right and up-down mirror symmetries while preserving C_2 symmetry—a feature crucial for the dynamic observation of projected EPs. Specifically, C_2 symmetry simplifies the total scattering matrix ($\mathbf{S_p}$) by mapping the scattering channels into two symmetric pairs (u_1 to d_1, u_2 to d_2). As derived in Supplementary Note 4, the exceptional topology of EP_1 and EP_2 is encoded in their respective vanishing matrix elements, t_e^{u1} and t_o^{u1}. These elements scatter waves from the same shared input channel (u_1) to entirely different output channels: t_e^{u1} routes to the bottom-left port (d_2), while t_o^{u1} routes to the bottom-right port (d_1). Therefore, this configuration significantly constrains the inter-system coupling between the projected subsystems $\mathbf{S_1}$ and $\mathbf{S_2}$. Consequently, the two induced projected EPs (EP_1 and EP_2) are firmly anchored at identical locations in the momentum-frequency domain. This spectral coincidence enables the simultaneous observation of STVs, governed by distinct exceptional topologies, along different spatial scattering channels under single-pulse excitation. When C_2 symmetry is broken, the coupling between subsystems becomes more complex, EP_2 split, destroying the location-anchoring with EP_1.

To elucidate this mechanism, we introduce a C_2 -preserving geometric perturbation Δw_1 to the original structure, as shown in Fig. S13a. In Fig. S13b, we illustrate the evolution of the spectral singularities (the phase vortex of vanishing entries t_e^{u1} in $\mathbf{S_1}$ and t_o^{u1} in $\mathbf{S_2}$) in momentum-frequency plane as Δw_1 varies. The trajectories reveal the merging of exceptional arcs (EAs) carrying opposite phase-winding charges (denoted by green and yellow dashed lines), obeying the global charge conservation. Throughout this C_2-protected evolution, EA_1 (containing EP_1) and EA_2 (containing EP_2) coincide exactly. Figure S13c tracks the eigenvalue sensitivity ($|\varepsilon_+ - \varepsilon_-|$) as a function of Δw_1 for three representative stages: two pre-merging points with opposite charges (purple and yellow circles in Fig. S13b) and the merging point (gray star in Fig. S13b). As Δw_1 varies, EP_1 possesses a square-root sensitivity, whereas EP_2 possesses a linear sensitivity, exhibiting their charge-1 and charge-2 topological properties.

In order to probe the impact of C_2 symmetry on the coupling between subsystems, we introduce a C_2-breaking perturbation Δw_2, for comparison with Δw_1 (shown in Fig. S13d). The evolution of the spectral singularities t_e^{u1} and t_o^{u1} with varied Δw_2 is shown in Fig. S13e. In stark contrast to the

C_2-preserving case, breaking C_2 symmetry causes EP_2 to split into two EPs with identical topological charges. In the momentum-frequency domain, one of these split EPs remains stationary, while the other participates in the opposite-charge merging process shown in Fig. S13a. This behavior demonstrates that the symmetry-breaking perturbations can induce the merging of projected EPs with identical topological charges, thereby forming a Dirac EP, which aligns with the previous study[1]. We demonstrate this merging process via the eigenvalue sensitivity as a function of wavenumber k (Fig. S13f). Prior to merging, the two EPs split from EP_2 exhibit square-root sensitivities. However, at the merging point where Δw_2=0 with the existence of C_2 symmetry, the sensitivity becomes linear. Collectively, these results highlight a paradoxical feature of the EP-STV correspondence: while projected EPs persist over a broad perturbation range due to the topological robustness guaranteed by global charge conservation, their intrinsically divergent sensitivity makes their specific momentum-frequency coordinates highly susceptible to shifting.

<u>Changes (page 5, 15-17) in revised manuscript:</u>

"We begin by considering a metagrating characterized by broken mirror-x and -z symmetry, but preserved C_{2y} rotational symmetry (Fig. 2a). …"

<u>Changes (page 8, 13-18) in revised manuscript:</u>

"Crucially, these specific subspaces are deliberately chosen because they share a common input channel (the top-left port, u_1). Then, the key elements responsible for the occurrence of the projected EPs (EP_1 and EP_2), t_e^{u1} and t_o^{u1}, scatter waves from the same shared input (u_1) to entirely different output channels: t_e^{u1} routes to the bottom-left port (d_2), while t_o^{u1} routes to the bottom-right port (d_1). This facilitates the simultaneous and spatially separated experimental observation of EP_1 and EP_2…"

<u>Changes (page 9, lines 11-12) in revised manuscript:</u>

"…This unique topology identifies EP_2 as a Dirac EP formed by the merging of two conventional charge-1 EPs [54, 55], which is protected by C_{2y} symmetry (see Supplementary Note 7 for details). …"

---

[1] Wu, Y., Zhu, D., Wang, Y., Rong, X. & Du, J. Experimental Observation of Dirac Exceptional Points. Phys. Rev. Lett. 134, 153601 (2025).

Changes (page 17, lines 18-22) in revised manuscript:

"⋯Notably, the resulting STVs inherit the salient feature of EPs, i.e., simultaneous topological robustness and high sensitivity: while STVs corresponding to EP_1 and EP_2 exhibit pronounced spectral shifts in response to external perturbations , their centers are firmly anchored together at the updated location, preserved by C_2 symmetry without being destroyed.⋯"

Response to Referee 3 -- NCOMMS-26-011336-T

Reviewer:

The manuscript presents an analysis of projected scattering exceptional points (EPs) generated during spatiotemporal dynamics, along with their experimental implementation on a liquid surface wave platform, revealing spatiotemporal vortices.

Although the experimentally realized system described in the article is closed and Hermitian—and thus evolves unitarily—a projection onto its subsystem(s) leads to an effective non-Hermitian description, with the resulting dynamics becoming non-unitary which enables the observation of EPs. In this sense, the projection of the 4×4 scattering matrix S_p, given in Eq. (1), onto two-by-two scattering sub-matrices S_1 and/or S_2 can be interpreted as treating these subspaces as the system of interest, while the remaining degrees of freedom act as an environment, in line with standard descriptions of losses, for example via a master equation. The sub-matrices S_1 and S_2 are non-Hermitian and can therefore exhibit singularities (e.g., EPs), as studied in the manuscript.

The authors claim to establish a direct cross-domain correspondence linking a static EP to a dynamic scattering singularity. While this is an interesting perspective, connections between static and dynamic EPs (defined in various ways) have been studied previously. For example, EPs of a non-Hermitian Hamiltonian (as mentioned in the manuscript and referred to as Hamiltonian EPs, H-EPs) are typically interpreted as static EPs, whereas EPs of a Liouvillian (referred to as Liouvillian EPs, L-EPs) correspond to dynamic EPs. The relation between H-EPs and L-EPs can be formulated within a hybrid (or q-deformed) Liouvillian framework [Minganti2020, Kumar2021], which was demonstrated experimentally in [Chen2021].

Overall, I find the presented experimental results interesting, novel, and potentially of significant interest to the readership of Nature Communications. However, before recommending publication, I suggest several improvements and clarifications, including citing a few closely related papers.

Response:

Thank you so much for the thorough and constructive review of our manuscript. Your comments have significantly improved the clarity and rigor of our work. We appreciate your recognition of the experimental results as “interesting, novel, and potentially of significant interest to the readership of Nature Communications.” We have added those relevant papers mentioned. Below, we address each of your comments point by point.

Reviewer:

(1) I suggest explicitly indicating in the title that the work concerns projected EPs, i.e., EPs obtained by projecting the full unitary, non-singular scattering matrix S_p onto lower-dimensional 2×2 subspaces. As stated in the manuscript, the intrinsic loss in the experimental metagrating is negligible; therefore, EPs cannot occur in the full matrix S_p. However, they can emerge in its nonunitary subspaces, as demonstrated by the authors. Thus, the title could be revised by adding the word "Projected," for example: “Spatiotemporal Dynamics of Projected Scattering Exceptional Points.”

This is an important conceptual point and should be emphasized not only in the title but also in the abstract—namely, that the observed EPs arise from projections of the unitary scattering matrix onto lower-dimensional subspaces.

Response:

Thank you for the important comment, and we have carefully considered it. However, we would like to respectfully share our perspective and a slight concern, hoping to seek your understanding. We believe the physics in our work can be viewed from two distinct layers:

1. **The intrinsic physical nature (The Phenomenon):** The unique spatiotemporal dynamics (e.g., spatiotemporal vortices, STVs) mathematically mapped and observed in our work represent an intrinsic topological feature of *general* scattering EPs. That is to say, even if a scattering EP were constructed in a genuinely non-Hermitian system (with actual material gain/loss), this universal spatiotemporal dynamic would still hold.
2. **The specific method of realization (The Platform):** Projecting the unitary scattering matrix onto lower-dimensional subspaces is our specific, chosen pathway to successfully construct and observe these EPs in a lossless physical platform.

Therefore, our primary concern is that revising the title to *'Spatiotemporal Dynamics of Projected Scattering Exceptional Points'* might inadvertently mislead readers into thinking that these spatiotemporal dynamics are an exclusive consequence of "projected" EPs, thereby unintentionally narrowing the broad physical universality of the EP-STV correspondence we aim to establish.

However, we fully agree with your core point that the specific realization mechanism (the projection) must be made completely transparent to the readers right from the beginning. To address this, we have followed your advice and extensively revised the **Abstract**. We now explicitly emphasize that our observed EPs arise from projecting the unitary scattering matrix onto lower-dimensional subspaces, ensuring no reader will misunderstand the nature of our experimental system.

We hope this justification for keeping the original title while clarifying the abstract seems reasonable to you. Please rest assured that this is merely our proposed handling of the issue to preserve the broad appeal of the physical findings. If you still feel strongly that adding "Projected" to the title is strictly necessary for scientific accuracy, we are absolutely open to modifying the title in the next round of revision."

Changes (page 2, lines 2-3) in revised abstract:

"…This enables the topology of EPs, which are realized here through projections of the unitary scattering matrix onto lower-dimensional subspaces, to be imprinted into a scattering wave packet, forming a spatiotemporal vortex (STV): a structured wave with intertwined phase singularities in space and time, anchored at the EP and carrying the quantized topological charge of the EP.…"

Changes (page 4, lines 1-3) in revised manuscript:

"…In this work, we transcend the steady-state picture of scattering EPs by investigating their spatiotemporal consequences, with these EPs constructed by projecting the full unitary scattering matrix onto lower-dimensional subspaces.…"

Reviewer:

(2) The authors emphasize that the novelty of the paper lies in its treatment of spatiotemporal dynamics. In this context, I suggest that the authors cite related works on spatiotemporal dynamics,

for example in photonic crystals [Sharabi2022, Bushuev2026], where exceptional points have also been studied.

Response:

We appreciate this suggestion. **We have cited the papers suggested in our revised introduction, which is inspiring and highly relevant to our work.**

Changes (page 3, lines 22, page 4, line 1) in revised manuscript:

"…The very nature of a scattering event is inherently dynamic and unfolds in both space and time[34], whereby the perspective of spatiotemporal dynamic offers new degree of freedoms for scattering manipulation[35, 36].…"

[35] Bushuev, V. A. & Mantsyzov, B. I. Spatiotemporal dynamics of formation and propagation of spectrally singular modes in a broadband quasi-PT-symmetric photonic crystal. Phys Rev. A 113, 033510 (2026).

[36] Sharabi, Y., Dikopoltsev, A., Lustig, E., Lumer, Y. & Segev, M. Spatiotemporal photonic crystals. Optica 9, 585–592 (2022).

Reviewer:

(3) The use of terms such as "revolutionized" or "groundbreaking" appears unnecessary and somewhat overstated. For example, the statement that "exceptional points have revolutionized the control of wave–matter interactions" seems exaggerated, even though exceptional points have indeed attracted considerable interest. Note, e.g., that it remains under debate whether they can enhance sensing or precision of measurements, and there are many works indicating that this may not be the case.

Similarly, referring to demonstrations of Hamiltonian exceptional points as "groundbreaking" may be too strong. A more balanced and precise wording would improve the tone of the manuscript.

Response:

We deeply appreciate you pointing this out. Indeed, some phrasing in the initial manuscript was somewhat overstated and unnecessarily hyperbolic. We have thoroughly reviewed the entire manuscript to ensure a neutral, factual, and professionally restrained tone throughout.

For example, in the revised manuscript:

"revolutionized" has been changed to "provided new opportunities for"

"groundbreaking" has been changed to "representative"

"demonstrate conclusively" has been changed to "provide robust evidence"

"definitive" has been changed to "compelling"

"inaccessible" has been changed to "elusive"

Reviewer:

(4) The first sentence of the abstract refers to non-Hermitian physics and cites two papers by Carl Bender et al. While these works are certainly important—particularly in enabling the analysis of non-Hermitian Hamiltonians (NHHs) in terms of parity–time (PT) symmetry—non-Hermitian physics has been central to quantum research long before the 1998 paper by Bender. Indeed, non-Hermitian systems have been studied for over 60 years, typically in the context of Markovian or non-Markovian master equations (i.e., Liouvillians) or Heisenberg–Langevin equations of motion.

Moreover, NHHs have been widely used since the 1990s within the quantum trajectory approach to solve master equations [Carmichael1993, Dalibard1992]. The similarities and differences between NHHs and Liouvillians underpin the relationship between the above-mentioned H-EPs and L-EPs.

In this context, I would suggest simplifying the phrase “a foundational framework for controlling wave–matter interactions” when referring to Bender's works to simply “a framework,” which would provide a more balanced and historically accurate description.

Response:

We are truly grateful to you for providing this broader and more historically accurate perspective. This comment highlights the profound and much longer history of non-Hermitian physics. Indeed, the study of non-Hermitian systems has been a cornerstone in quantum mechanics for over 60 years, deeply rooted in the contexts of master equations, Liouvillians, and the quantum trajectory approach. Following the suggestion, we have revised the text in the abstract and introduction.

Changes (page 2, lines 13-15) in revised manuscript:

"The study of non-Hermitian physics possesses a profound history of over 60 years in quantum research [1, 2]. Building upon these quantum origins and through the introduction of parity-time symmetry [3, 4], it has developed into a framework for controlling wave–matter interactions.⋯"

[1] Dalibard, J., Castin, Y. & Mølmer, K. Wave-function approach to dissipative processes in quantum optics. Phys. Rev. Lett. 68, 580–583 (1992).

[2] Carmichael, H. J. Quantum trajectory theory for cascaded open systems. Phys. Rev. Lett. 70, 2273–2276 (1993).

Reviewer:

(5) Degeneracies in the spectra of matrices do not necessarily correspond to EPs; in general, they may also correspond to diabolical points [Berry1984, Arkhipov2023], for which the corresponding eigenvectors are orthogonal. I assume that the authors have verified not only the eigenvalues but also the eigenvectors at the identified singularities, and that all the reported cases indeed correspond to exceptional points for which the corresponding eigenvectors are coalescent. Nevertheless, it would be helpful to include a clear statement confirming that no diabolical points are observed in the system.

(6) Moreover, Fig. 2 shows a Dirac-like degeneracy. Such a degeneracy does not necessarily correspond to an exceptional point; it may instead represent a diabolical point—the concept originally introduced by Michael Berry [Berry1984], named for its resemblance to a diabolo toy. This raises the question of whether the authors have verified the nature of the corresponding

eigenvectors. In particular, it would be important to check whether the eigenvectors coalesce, as expected for exceptional points, or remain orthogonal, as is typical for diabolical points.

Response:

Thank you for raising these two highly rigorous and closely related questions. Since both comments concern the fundamental distinction between exceptional points (EPs) and diabolical points (DPs), we address them together here.

First, we greatly appreciate your reminder regarding the rigorous verification of the eigenvectors. Indeed, eigenvalue degeneracy alone is insufficient to claim an EP, as orthogonal eigenvectors would indicate a DP. While we had indeed verified that the reported cases correspond to true EPs during our research, this critical eigenvector information was not explicitly presented in our original manuscript.

To address this, we have now added a quantitative eigenvector-coalescence analysis in the new **Supplementary Note 8 ("Eigenvector coalescence of EP_1 and EP_2")**. In this section, we calculate and display **the phase rigidity near the singularities**. The results unambiguously demonstrate that the phase rigidity strictly approaches zero at both EP_1 and EP_2, which is the definitive mathematical hallmark of eigenvector coalescence[2]. Consequently, we have added **a clear statement in the revised main text confirming that the eigenvectors are indeed coalescent** and explicitly ruling out the presence of DPs.

Second, regarding your specific observation in Comment 6, which is about the Dirac-like degeneracy shown in Fig. 2e. Unlike the typical branch-cut Riemann surfaces of fundamental EPs (e.g. the EP_1 from $\mathbf{S_1}$), the Riemann surface of our charge-2 EP (EP_2 from $\mathbf{S_2}$) exhibits a distinct Dirac-like crossing. It is natural that such a spectrum would raise suspicions of a DP. **However, as quantitatively proven by the aforementioned phase rigidity analysis, this degeneracy is unequivocally an EP because its eigenvectors strictly coalesce.**

We realize that, just as you astutely pointed out, readers will very likely have the exact same concern when observing this Dirac-like eigenvalue behavior. **Here, we note that this is a special state, i.e. a Dirac EP, which exhibits linear-splitting eigenvalues while holds collapsed**

[2] Günther U., Rotter, I., Samsonov, B. F. Projective Hilbert space structures at exceptional points. J. Phys. A-Math. Theor. 40, 8815 (2007).

eigenvectors, has been reported in Refs. [Rivero2022] and [Wu2025]. Therefore, we have added a dedicated clarification in the main text accompanying Fig. 2. We now explicitly emphasize that while this singularity is cloaked in the facade of a DP (the way of eigenvalue degeneracy), it fundamentally possesses the soul of an EP (eigenvector coalescence). This highlights the unique topological feature of the charge-2 EP observed here.

[Rivero2022] Rivero, J. H. D., Feng, L. & Ge, L. Imaginary Gauge Transformation in Momentum Space and Dirac Exceptional Point. Phys. Rev. Lett. 129, 243901 (2022).

[Wu2025] Wu, Y., Zhu, D., Wang, Y., Rong, X. & Du, J. Experimental Observation of Dirac Exceptional Points. Phys. Rev. Lett. 134, 153601 (2025).

Added part in Supplementary Information as Supplementary Note 8:

Eigenvector coalescence of EP_1 and EP_2

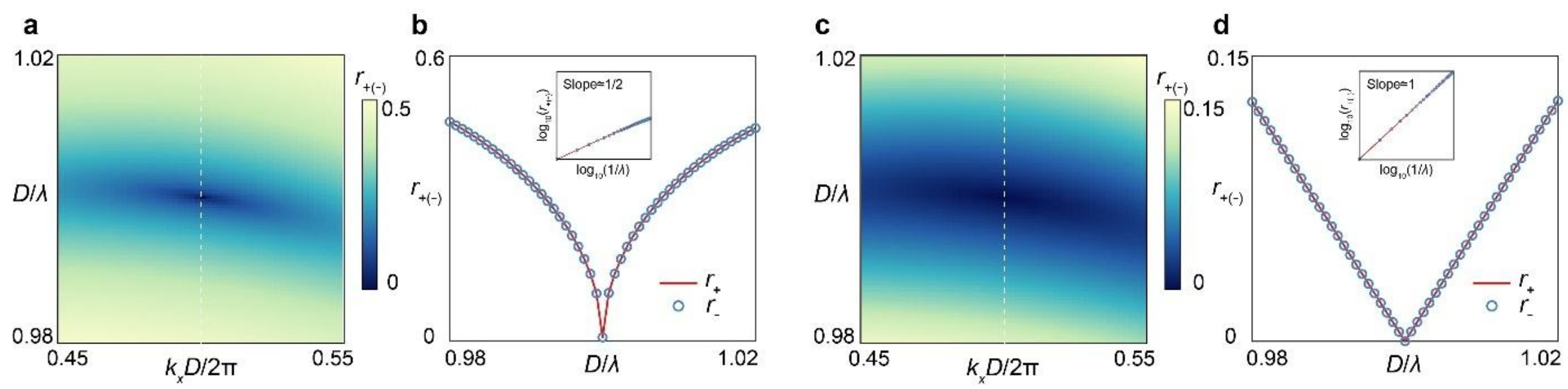

Figure S14 Simulated phase rigidity $r_{+(-)}$ of eigenvectors around EP_1 (**a**) and EP_2 (**c**) in momentum-frequency domain. **b** and **d** are results extracted from **a** and **c** (white dash line), the insets exhibit the logarithmic plots. The *x*- and *y*-axis ranges in logarithmic insets of **b** and **d** are the same.

In order to investigate the eigenvector features of projected EPs, we show the phase rigidity distribution of EP_1 and EP_2 in Fig. S14 obtained from simulated data, which characterizes the degree of "skew" between the eigenvectors, and could be defined by

$$r_{+(-)} = \frac{\langle\tilde{\psi}^L_{+(-)}|\tilde{\psi}^R_{+(-)}\rangle}{\langle\tilde{\psi}^R_{+(-)}|\tilde{\psi}^R_{+(-)}\rangle} \tag{S40}$$

where $\langle\tilde{\psi}^L_{+(-)}| = \frac{\langle\psi^L_{+(-)}|}{\langle\psi^L_{+(-)}|\psi^L_{+(-)}\rangle}$ and $|\tilde{\psi}^R_{+(-)}\rangle = \frac{|\psi^R_{+(-)}\rangle}{\langle\psi^L_{+(-)}|\psi^L_{+(-)}\rangle}$ are the normalized biorthogonal left eigenvectors and right eigenvectors, and $\langle\psi^L_{+(-)}|$ and $|\psi^R_{+(-)}\rangle$ are left and right eigenvectors corresponding to the eigenvalues. Due to the biorthogonal condition, $\langle\tilde{\psi}^L_{+(-)}|\tilde{\psi}^R_{+(-)}\rangle = \delta_{+-}$, so

that the phase rigidity can be expressed as $1/\langle\tilde{\psi}^{R}_{+(-)}|\tilde{\psi}^{R}_{+(-)}\rangle$. For a 2-order EP-degeneracy, $r_+ = r_- = 0$, demonstrating the coalescence of two eigenvectors at EP.

It is evident that $r_{+(-)}$ approaches 0 at EP_1 and EP_2 simultaneously, shown in Fig. S14a and S14c. However, the trends of phase rigidity near EP_1 and EP_2 are strikingly different, displayed by Fig. S14b and S14d. For EP_1, the phase rigidity exhibits a square-root behavior (the approximately 1/2-slope in logarithmic coordinate, the inset of Fig. S14b), demonstrating its typical EP-like topological characteristics. For EP_2, the phase rigidity exhibits a linear behavior (the approximately 1- slope in logarithmic coordinate, the inset of Fig. S14d), demonstrating its Dirac-like topological features, like Ref [Wu2025].

[Wu2025] Wu, Y., Zhu, D., Wang, Y., Rong, X. & Du, J. Experimental Observation of Dirac Exceptional Points. Phys. Rev. Lett. 134, 153601 (2025).

<u>Changes (page 9, lines 13-17) in revised manuscript:</u>

"···We further investigate the behavior of the corresponding eigenstates near the singularities. The phase rigidity strictly approaches zero at both EP_1 and EP_2, which is the definitive mathematical hallmark of eigenvector coalescence (see Supplementary Note 8 for details). This also highlights the topological uniqueness of EP_2: as a Dirac EP, it is cloaked in a diabolic-point[56,57] facade through its eigenvalue degeneracy, yet retains the nature of an EP through eigenvector coalescence.···"

[56] Berry, M. V. & Wilkinson, M. Diabolical points in the spectra of triangles. Proc. R. Soc. London A 392, 15–43 (1984).

[57] Arkhipov, I. I., Miranowicz, A., Minganti, F., ¨Ozdemir, K. & Nori, F. Dynamically crossing diabolic points while encircling exceptional curves: A programmable symmetric-asymmetric multimode switch. Nat. Commun. 14, 2076 (2023).

Reviewer:

(7) The input and output wave vectors on the left- and right-hand sides of Eq. (1) should be distinguished by different symbols. Currently, they are denoted by the same wave vectors, which is misleading.

Response:

Thanks for the comment. We have modified this notation. Input wave vectors are now denoted as **a** and output wave vectors as **b**, making the distinction clear: $\mathbf{b}=\mathbf{S_p a}$. The corresponding descriptions and figure legends in terms of input and output wave vectors have also been updated.

Changes of Equation 1 in revised manuscript:

"…

$$\mathbf{b} = \mathbf{S_p}(k_x, \omega)\mathbf{a} = \begin{bmatrix} r_e^{\mathrm{u1}} & r_o^{\mathrm{u2}} & t_o^{\mathrm{d1}} & t_e^{\mathrm{d2}} \\ r_o^{\mathrm{u1}} & r_e^{\mathrm{u2}} & t_e^{\mathrm{d1}} & t_o^{\mathrm{d2}} \\ t_o^{\mathrm{u1}} & t_e^{\mathrm{u2}} & r_e^{\mathrm{d1}} & r_o^{\mathrm{d2}} \\ t_e^{\mathrm{u1}} & t_o^{\mathrm{u2}} & r_o^{\mathrm{d1}} & r_e^{\mathrm{d2}} \end{bmatrix}\mathbf{a}, \quad (1)$$

…"

Changes (page 7, lines 3-7) in revised manuscript:

"…where the scattering matrix $\mathbf{S_p}$ is composed of reflection and transmission coefficients (denoted r and t) of ordinary and anomalous scattering modes (identified by subscripts o and e) under incidence from different ports (see details in Supplementary Note 1). $\mathbf{S_p}$ relates the output state $\mathbf{b} = (b_{\mathrm{u1}}, b_{\mathrm{u2}}, b_{\mathrm{d1}}, b_{\mathrm{d2}})^{\mathrm{T}}$ to the input $\mathbf{a} = (a_{\mathrm{u1}}, a_{\mathrm{u2}}, a_{\mathrm{d1}}, a_{\mathrm{d2}})^{\mathrm{T}}$, both representing the complex pressure amplitudes at the four ports.…"

Reviewer:

(8) It is somewhat unfortunate, from the perspective of EP studies, that the full 4×4 scattering matrix is unitary in their experiment. If it were nonunitary, the system could allow for a richer structure of spectral degeneracies, including exceptional points, diabolical points, and possibly hybrid exceptional – diabolical degeneracies in a genuinely lossy setting.

Thus, the question arises whether it is experimentally feasible to introduce controlled losses into the 4×4 system such that the resulting scattering matrix becomes non-unitary in the platform used in the manuscript. I am not suggesting that such extended experiments are necessary, but I believe it would be valuable for the reader to hear some comments on a possible

generalization of the experiment—one that incorporates genuine losses in the 4×4 system, rather than effectively simulating losses by restricting attention to a sub-block of a larger unitary matrix S_p.

Response:

Thank you for this inspiring, forward-looking comment. We completely agree with your perspective.

We first answer your question regarding experimental feasibility: yes, introducing controlled genuine losses into our liquid surface wave platform (or other wave platforms such as optics and acoustics) is experimentally feasible. This could be achieved, for instance, by precisely integrating specifically designed absorbers into the metagrating units, thereby transforming the platform into a genuinely non-Hermitian 4×4 scattering system.

As you pointed out, stepping beyond the "projected" non-Hermitian paradigm into a truly lossy full 4×4 system would unlock a profoundly richer landscape of spectral degeneracies. In such a fundamentally non-unitary full scattering matrix (rather than its sub-matrices), it would become mathematically and physically possible to realize a 4th-order EP or even multiple coexisting 3rd-order EPs. Consequently, under dynamic wave-packet incidence, these higher-order static singular topologies could map onto even more complex spatiotemporal dynamics, potentially generating exotic higher-charge STVs, STV clusters, or even interacting vortex networks.

We find this to be a fascinating and promising direction. We deeply appreciate your visionary insight, which not only elevates the depth of our current work but also illuminates the path forward. Following the suggestion, we have added a discussion regarding this possible generalization in the **Conclusion** section of the revised manuscript, aiming to provide readers with a broader and richer physical outlook. And we will experimentally explore this genuinely lossy higher-order EP direction in our next phase of research. Thanks again.

<u>**Changes (page 18, lines 2-6) in revised manuscript:**</u>

"···Furthermore, while the non-Hermiticity in this work arises from projecting a unitary scattering matrix, introducing controlled genuine losses could unlock a profoundly richer landscape of spectral degeneracies. Such lossy systems would support global higher-order EPs that map onto

even more complex spatiotemporal dynamics under wave-packet incidence, including higher-charge STVs, STV clusters, or interacting vortex networks.”

Reviewer:

(9) The analysis of sub-matrices of a unitary 4×4 matrix considerably simplifies the problem, not only from a mathematical perspective but also in terms of physical interpretation. The authors focus on two sub-matrices, S_1 and S_2, presented on page 8. However, many other sub-matrices could be considered, some of which may also exhibit exceptional points. While I do not insist that the authors provide a more detailed analysis of additional sub-matrices, a few clarifying remarks would be beneficial for the reader. In particular, it would be helpful to explain why these two specific sub-matrices were chosen, and whether other sub-matrices may display interesting nontrivial features, including exceptional points and possibly the emergence of diabolical points.

Response:

Thank you for this insightful comment, an observation that intriguingly aligns with the feedback from another Reviewer. We agree that adding investigation of other sub-matrices, as well as clarifying the selection rules for the sub-matrices ($\mathbf{S_1}$ and $\mathbf{S_2}$) provide a much richer context for the readers.

According to the comment, a more comprehensive analysis of the sub-matrices has been added in the new Supplementary Note 3 (“Constructing rule of projected EP”). Based on the intrinsic symmetries of the metagrating system, the full 4-dimensional scattering matrix $\mathbf{S_p}$ can be mathematically projected into various 2-dimensional sub-matrices. By selecting 2 out of the 4 input ports and 2 out of the 4 output ports, there are in total 36 possible 2×2 sub-matrices. However, due to the strict mathematical requirement for the emergence of an EP (i.e., forming a non-diagonalizable Jordan block), **the number of subsystems capable of hosting a projected EP is far fewer than 36**. We show that there are only two pairs of sub-matrices supporting the projected EPs: $\mathbf{S_1}$ and $\mathbf{S_3}$ (supporting EP_1), and $\mathbf{S_2}$ and $\mathbf{S_4}$ (supporting EP_2). As detailed in the revised Supplementary Information, we compared the EPs induced by these different valid sub-matrices.

While their exact eigenvalue Riemann surfaces differ slightly, they share identical eigenvalue topologies (q_ϵ =1 for EP_1 and q_ϵ =2 for EP_2).

Regarding why $\mathbf{S_1}$ and $\mathbf{S_2}$ were specifically highlighted in this work, this selection was entirely driven by the experimental design for the observation of these topological features. These specific channel combinations directly correspond to the physical excitation configurations used in our experiments. Crucially, $\mathbf{S_1}$ and $\mathbf{S_2}$ are uniquely advantageous because they deliberately share the exact same input channel (u_1) but route the vanishing scattering elements to entirely different output channels (t_e^{u1} routes to the bottom-left port d_2, while t_o^{u1} routes to the bottom-right port d_1). This specific configuration spatially separates the transmitted fields into distinct left and right sections. **Consequently, it enables the independent and simultaneous experimental observation of the topological signatures from two different EPs under a single wave-packet incidence, completely avoiding spatial overlap.**

In addition to projected EP, the projected subsystems can also exhibit other unique topological features, including DPs. Here's an example, the subsystem characterized by $\mathbf{S_{DP}}$ as $\mathbf{b}_\mathrm{U} = \mathbf{S}_\mathrm{DP}\mathbf{a}_\mathrm{L}$, where $\mathbf{b}_\mathrm{U} = [b_{\mathrm{u1}}, b_{\mathrm{u2}}]^\mathrm{T}$, $\mathbf{a}_\mathrm{L} = [a_{\mathrm{u1}}, a_{\mathrm{d2}}]^\mathrm{T}$ and $\mathbf{S_{DP}} = [r_e^{\mathrm{u1}}, t_e^{\mathrm{d2}}; r_o^{\mathrm{u1}}, t_o^{\mathrm{d2}}]$. Here abs($\mathbf{S_{DP}}$) = [1, 0; 0, 1], hosting a DP with degenerated eigenvalues ($\lambda_1 = \lambda_2$) and orthogonal eigenvectors ($\lambda_1 = [1 \quad 0]^T, \lambda_2 = [0 \quad 1]^T$), thereby exhibiting a Dirac-like double-conical degeneracy. The similar scattering DP has also been reported in our previous paper [Xiao2025[3]]. Unfortunately, for a DP, we haven't observed the direct equivalence between the eigenvalue topology of DP and the scattering spectral phase-winding topology of STV. Therefore, the exploration of DP is beyond the scope of this work.

According to your comment, we have added a brief clarifying remark in the main text accompanying the definitions of $\mathbf{S_1}$ and $\mathbf{S_2}$ to explain this specific choice, and directed interested readers to Supplementary Note 3 for the exhaustive sub-matrix analysis.

Changes (page 8, 13-18) in revised manuscript:

"Crucially, these specific subspaces are deliberately chosen because they share a common input channel (the top-left port, u_1). Then, the key elements responsible for the occurrence of the

---

[3] Xiao, Y., et al. Evolution of pairwise scattering exceptional points via acoustic metasurfaces. Sci. China Phys. Mech. Astron. 69, 214311 (2026).

projected EPs (EP_1 and EP_2), t_e^{u1} and t_o^{u1}, scatter waves from the same shared input (u_1) to entirely different output channels: tu1e routes to the bottom-left port (d_2), while t_o^{u1} routes to the bottom-right port (d_1). This facilitates the simultaneous and spatially separated experimental observation of EP_1 and EP_2⋯"

Changes (page 9, lines 17-21) in revised manuscript:

"Note that the observed topological features are tied to the specific choice of subsystem decomposition, as each subsystem corresponds to a distinct set of physical excitation and observation channels. Hence, the existence of EP_1 and EP_2 in the specific subspaces implies that their topological features can only be excited by the eigen-input states corresponding to those chosen subsystems"

Added part in Supplementary Information as Supplementary Note 3:

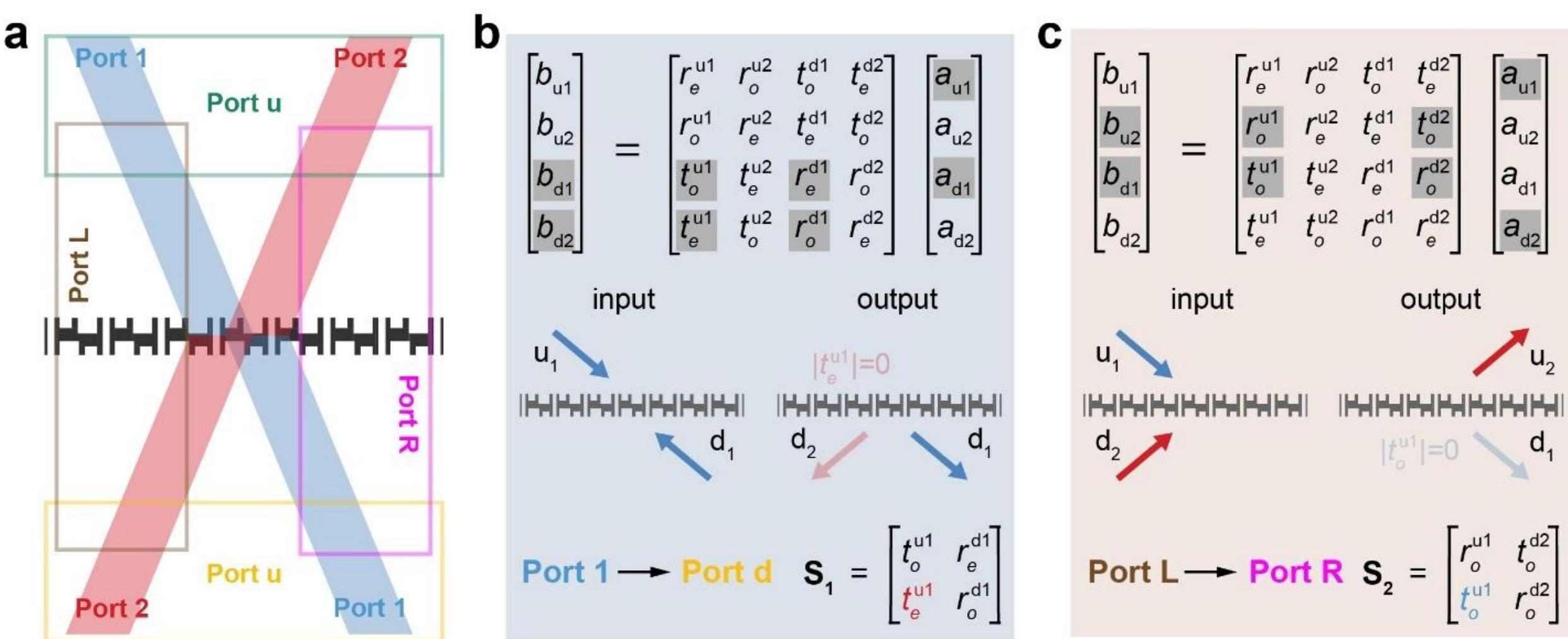

Figure S4 a, The full space is divided into six 2-dimensional subspaces by the intrinsic symmetry of the metagrating. Specifically, ports u and d (vertical half spaces, colored by green and yellow), ports 1 and 2 (diagonal half-spaces, colored by blue and red), ports L and R (horizontal half spaces, colored by brown and magenta). **b** and **c**, Detailed projection process and input-output behaviors of $\mathbf{S_1}$ and $\mathbf{S_2}$ mentioned in the main text.

For the designed metagrating system shown in the main text, the element-wise absolute value of its full scattering matrix takes the form abs($\mathbf{S_p}$) = [1, 0, 0, 0; 0, 0, 0, 1; 0, 0, 1, 0; 0, 1, 0, 0]. Here we divide the full space with six 2-dimensional subspaces by the intrinsic symmetry of the metagrating, shown by Fig. S4a. To be specific, we divide the full 4-dimentional space into paired subspaces, including the diagonal and anti-diagonal half-spaces defined by C_2 rotation symmetry

as port 1 and port 2, the upper and lower half-spaces defined by up-down mirror symmetry as port u and port d, and the left and right half-spaces defined by left-right mirror symmetry as port L and port R. In principle, by selecting 2 out of the 4 input ports and 2 out of the 4 output ports, there are in total 36 possible 2×2 sub-matrices (i.e. 36 2-dimentional subspaces). However, due to the strict mathematical requirement for the emergence of an EP (i.e., forming a non-diagonalizable Jordan block), the number of subsystems capable of hosting a projected EP is far fewer than 36. Mathematically, a second-order Jordan block requires that only one of its off-diagonal elements vanishes. Given that the scattering matrix $\mathbf{S_p}$ of our designed metagrating contains only four non-zero scattering elements, there are only two pairs of sub-matrices that support the projected EPs: $\mathbf{S_1}$, $\mathbf{S_2}$, $\mathbf{S_3}$ and $\mathbf{S_4}$. Among these, $\mathbf{S_1}$ and $\mathbf{S_2}$ have already been discussed in the main text. Below, we will detail how they are obtained through projection operations, and then we will introduce the other two EP-supporting subspaces, $\mathbf{S_3}$ and $\mathbf{S_4}$.

First, we introduce the corresponding input and output projection operators to show the projection process, using $\mathbf{S_1}$ and $\mathbf{S_2}$ as examples. Since the projected subspaces involve asymmetric input-output channel combinations, they are defined by the asymmetric-projection relation as $\mathbf{S}_{1,2} = \mathbf{P}_n^{\mathrm{out}}\mathbf{S}_{\mathbf{p}}\left(\mathbf{P}_n^{\mathrm{in}}\right)^{\mathrm{T}}$ (n=1,2,3,4, indicates the subsystem index), where $\mathbf{P}_n^{\mathrm{in}}$ projects the excitation channels and $\mathbf{P}_n^{\mathrm{out}}$ "selects" the corresponding scattering channels. For $\mathbf{S_1}$ and $\mathbf{S_2}$, $\mathbf{P_1^{in}}$ =[1, 0, 0, 0; 0, 0, 1, 0], $\mathbf{P_1^{out}}$ =[0, 0, 1, 0; 0, 0, 0, 1] and $\mathbf{P_2^{in}}$ =[1, 0, 0, 0; 0, 0, 0, 1], $\mathbf{P_2^{out}}$ =[0, 1, 0, 0; 0, 0, 1, 0]. These asymmetric projection operators provide a clear delineation between the excitation channels and the scattering channels. Physically, $\mathbf{S_1}$ and $\mathbf{S_2}$ represents the input-output behaviors from port 1 (L) to port d (R), and has been discussed in main text. Fig. S4b and S4c show the detailed projection process and input-output behaviors in subspaces of $\mathbf{S_1}$ and $\mathbf{S_2}$.

Then, we investigate the projected EPs of $\mathbf{S_3}$ and $\mathbf{S_4}$. $\mathbf{S_3}$ reveals the inter-port scattering behaviors from port L to port 2 (shown in Fig. S5a): $\mathbf{b_3} = \mathbf{S_3 a_3}$, where $\mathbf{b_3} = (b_{\mathrm{u2}}, b_{\mathrm{d2}})^{\mathrm{T}}$, $\mathbf{a_3} = (a_{\mathrm{u1}}, a_{\mathrm{d2}})^{\mathrm{T}}$ and $\mathbf{S_3} = [r_o^{\mathrm{u1}}, t_o^{\mathrm{d2}}; t_e^{\mathrm{u1}}, r_e^{\mathrm{d2}}]$. Similarly, $\mathbf{S_4}$ defines the scattering behaviors from port u to port d (shown in Fig. S5b): $\mathbf{b_4} = \mathbf{S_4 a_4}$, where $\mathbf{b_4} = (b_{\mathrm{d2}}, b_{\mathrm{d1}})^{\mathrm{T}}$, $\mathbf{a_4} = (a_{\mathrm{u1}}, a_{\mathrm{u2}})^{\mathrm{T}}$ and $\mathbf{S_4} = [t_e^{\mathrm{u1}}, t_o^{\mathrm{u2}}; t_o^{\mathrm{u1}}, t_e^{\mathrm{u2}}]$.

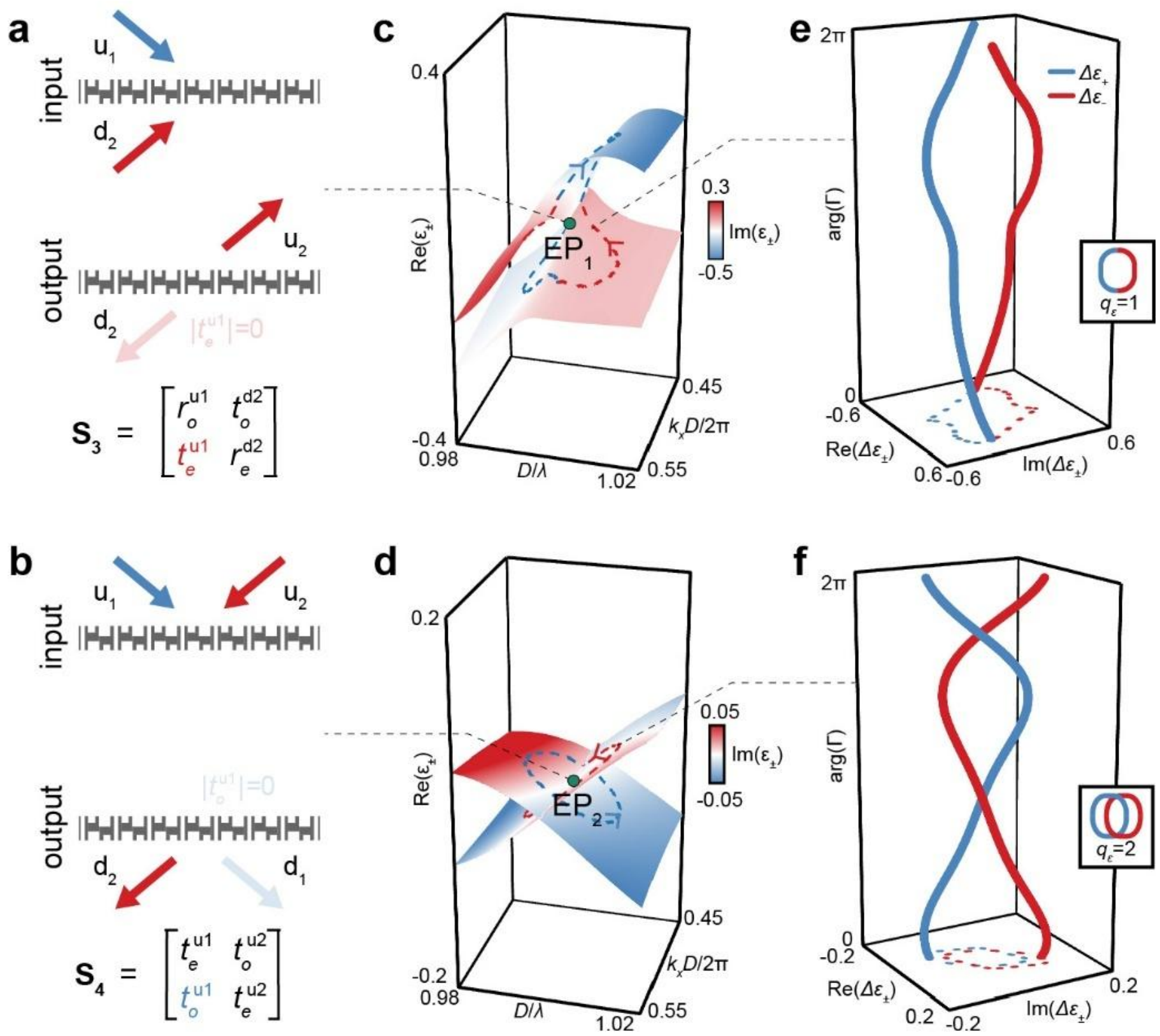

Figure S5 a and **b**, Inter-port scattering behaviors of the two subsystems described by the sub-matrices **S_3** and **S_4**. **c** and **d**, Eigenvalue Riemann surfaces of **S_3** and **S_4**, which exhibit the topological structures of EP_1 (**d**) and EP_2 (**e**), similar with the results of EP_1 and EP_2 shown in Fig. 2(d-e). **e-f**, Adiabatic eigenvalue evolution around EP_1 (**e**) for sub-matrix **S_3** and EP_2 (**ff**) for sub-matrix **S_4**, confirming the two-band charge $q_\epsilon = 1$ and 2, consistent with the results in Fig. 2f and 2h.

Figures S5c-d illustrate the simulated eigenvalue Riemann surfaces of **S_3** and **S_4** around the operated point in momentum-frequency domain. The EPs of **S_1** (main text, Fig. 2d) and **S_3** (Fig. S5d) share the identical branch-cut topologies; both are charge-1 EPs. Likewise, the EPs encoded of **S_2** ((main text, Fig. 2e) and **S_4** (Fig. S5d) share the identical Dirac-like intersecting topologies; both are charge-2 EPs. Notwithstanding that the two Riemann surfaces of the EPs from **S_1** and **S_3** are not exactly the same due to differences in their entries, they share the same topological nature; and the same holds for **S_2** and **S_4**. Encircling the charge-1 EP and charge-2 EP yield the adiabatic evolution of eigenvalue differences for **S_3** and **S_4** as shown in Fig. S5e and S5f, consistent with the results of Fig. 2f and 2h in main text. These comparisons between **S_1** (**S_2**) and **S_3** (**S_4**) demonstrate that the topological invariants of these projected EPs are determined by the vanishing element in their sub-matrices and remain robust regardless of the choice of other elements, which will be

proved in Supplementary Note 4. Noted that due to the preserved C_2 symmetry, the conclusions obtained by the subsystem with t_e^{u1} (t_o^{u1}) as vanishing element are identical to those for the subsystem t_e^{d1} (t_o^{d1}) as vanishing element. In addition, by appropriately selecting the projected subsystems, it is also possible to generate other degeneracies besides EPs, such as diabolic points (DPs).

Reviewer:

(10) The authors state that "EPs are associated with the evolution of confined modes in closed spaces." on page 2. However, in most cases, EPs are demonstrated in open systems that are either dissipative or include gain. While there are theoretical predictions of non-Hermitian dynamics in effectively non-dissipative systems—for example, under the presence of squeezing [Wang2019, Wakefield2024]—I find the current statement in the manuscript somewhat misleading. I do not expect the authors to cite additional references on such systems, but it would be preferable to clarify that EPs are typically associated with open systems, in contrast to the description given here and, for instance, in Ref. [43].

Response:

Thank you for pointing this out, an observation that aligns perfectly with the feedback from another Reviewer. We completely agree that our original phrasing could inadvertently be misleading. You are right, both H-EPs and S-EPs are associated to open systems interacting with the environment. **To clear up this misunderstanding, we have revised the related description in the introduction.**

Changes in the abstract (page 1, lines 15-17) in revised manuscript:

"While traditional EPs are associated with the Hamiltonians, characterizing the coalescence of intrinsic eigenstates, scattering EPs shift the perspective toward input-output responses, offering a powerful framework for wave control in free space. …"

Changes in the introduction (page 2, lines 21-22 and page 3, lines 1) in revised manuscript:

"Conventionally, these demonstrations predominantly revolve around Hamiltonian EPs (H-EPs), which emerge as degeneracies of the non-Hermitian effective Hamiltonian, associated with the coalescence of intrinsic eigenstates."

Changes in the introduction (page 3, lines 4-7) in revised manuscript:

"When considering multi-port scattering problems, nevertheless, Hamiltonian could not directly describe scattering modes behaviors[18]. Open structures such as metagratings[5,25] are more appropriately modeled by a scattering matrix, which fully characterizes the wave-structure interactions in free space. In light of this, the Hamiltonian-centric view has been fruitfully complemented by the concept of the scattering EP (S-EP) [28,29].…"

Reviewer:

(11) As mentioned above, it is worth noting in the manuscript that EPs are not limited to singularities of Hamiltonians or scattering matrices. Hamiltonian EPs, which neglect thermal and quantum jump processes, can be generalized to Liouvillian EPs in a full quantum description. Liouvillian exceptional points (L-EPs) have been introduced for both Markovian [Minganti2019] and non-Markovian [Lin2025] systems, and have been experimentally demonstrated on various platforms, including circuit QED [Chen2021, Chen2022, Zhang2025] and ion traps [Zhang2022, Bu2023, Bu2024].

Response:

Thank you for this comprehensive perspective. Indeed, confining the background discussion exclusively to Hamiltonians and scattering matrices would indeed omit a crucial and highly active frontier in quantum physics. We have reviewed the studies you recommended on this topic and believe they play an important role in the development of non-Hermitian physics. **We have added some descriptions about this field in the revised introduction and have appropriately cited some papers mentioned.**

Changes in the introduction (page 3, lines 1-4) in revised manuscript:

"…H-EPs, which neglect thermal and quantum jump processes, can be generalized to Liouvillian EPs (L-EPs) in a full quantum description[19-21]. Such L-EPs have been introduced for both Markovian[22] and non-Markovian[23] systems, and experimentally demonstrated on various quantum platforms[19,24].…"

Reviewer:

(12) Various types of singularities of scattering matrices—including exceptional points, as well as diabolical points and hybrid exceptional–diabolical degeneracies—have been experimentally observed (see, e.g., [Erb2025, Shaibe2025] and the references therein).

Response:

Thanks for the comment. These have been added as references in revised manuscript.

Reviewer:

In conclusion, I find the paper interesting, especially its experimental results. With appropriate clarifications and by citing some of the references mentioned here, I would be able to recommend the manuscript for publication in Nature Communications.

-- I include the following references for completeness of the report, but I do not require that all of them be cited in the manuscript.

[Minganti2020] F. Minganti et al., Hybrid-Liouvillian formalism connecting exceptional points of non-Hermitian Hamiltonians and Liouvillians via postselection of quantum trajectories, Phys. Rev. A 101, 062112 (2020).

[Kumar2021] P. Kumar, K. Snizhko, and Y. Gefen, Near-unit efficiency of chiral state conversion via hybrid-Liouvillian dynamics, Phys. Rev. A 104, L050405 (2021).

[Chen2021] W. Chen et al., Quantum Jumps in the Non-Hermitian Dynamics of a Superconducting Qubit Phys. Rev. Lett. 127, 140504 (2021).

[Sharabi2022] Y. Sharabi et al., Spatiotemporal photonic crystals, Optica 9, 585 (2022).

[Bushuev2026] V. A. Bushuev and B. I. Mantsyzov, Spatiotemporal dynamics of formation and propagation of spectrally singular modes in a broadband quasi-symmetric photonic crystal, Phys. Rev. A 113, 033510 (2026).

[Carmichael1993] H. J. Carmichael, Quantum Trajectory Theory for Cascaded Open Systems, Phys. Rev. Lett. 70, 2273 (1993).

[Dalibard1992] J. Dalibard, Y. Castin, and K. Molmer, Wave-Function Approach to Dissipative Processes in Quantum Optics, Phys. Rev. Lett. 68, 580 (1992).

[Arkhipov2023] I. I. Arkhipov et al., Dynamically encircling an exceptional curve by crossing diabolic points: A programmable multimode switch, Nat. Comm. 14, 2076 (2023)

[Berry1984] M. V. Berry and M. Wilkinson. “Diabolical points in the spectra of triangles”. Proc. R. Soc. London A 392, 15 – 43 (1984).

[Wang2019] Y.-X. Wang and A. A. Clerk, Non-Hermitian dynamics without dissipation in quantum systems, Phys. Rev. A 99, 063834 (2019).

[Wakefield2024] R. Wakefield, A. Laing, Y. N. Joglekar, Non-Hermiticity in quantum nonlinear optics through symplectic transformations, Appl. Phys. Lett. 124, 201103 (2024).

[Minganti2019] F. Minganti et al., Quantum exceptional points of non-Hermitian Hamiltonians and Liouvillians: The effects of quantum jumps, Phys. Rev. A 100, 062131 (2019).

[Lin2025] J.-D. Lin et al., Non-Markovian Quantum Exceptional Points, Nature Commun. 16, 1289 (2025).

[Chen2022] W. Chen et al., Decoherence Induced Exceptional Points in a Dissipative Superconducting Qubit, Phys. Rev. Lett. 128, 110402 (2022).

[Zhang2022] J.-W. Zhang et al., Dynamical control of quantum heat engines using exceptional points, Nat. Commun. 13, 6225 (2022).

[Bu2023] J.-T. Bu et al., Enhancement of Quantum Heat Engine by Encircling a Liouvillian Exceptional Point, Phys. Rev. Lett. 130, 110402 (2023).

[Bu2024] J.-T. Bu et al., Chiral quantum heating and cooling with an optically controlled ion, Light: Sci. App. 13, 143 (2024).

[Zhang2025] H.-L. Zhang et al., Experimental Observation of Non-Markovian Quantum Exceptional Points, Phys. Rev. Lett. 135, 230203 (2025).

[Erb2025] J. Erb et al., Topology and manipulation of scattering singularities in complex non-Hermitian systems: Two-channel case Phys. Rev. Res. 7, 023090 (2025).

[Shaibe2025] N. Shaibe et al., Superuniversal Statistics of Complex Time Delays in Non-Hermitian Scattering Systems, Phys. Rev. Lett. 134, 147203 (2025).

Response:

Thank you so much for the positive overall evaluation. The comprehensive list of references provided has been carefully reviewed, and the relevant works have been appropriately incorporated into the revised manuscript to ensure completeness and historical accuracy.

In closing, we would like to express our gratitude for the time and effort dedicated to this review. The professional comments, the precise insights into the underlying physics, and the profound perspective provided have been highly beneficial. They have not only significantly elevated the rigor of this manuscript but also broadened our horizons for future research. We consider ourselves truly fortunate to have received your professional review of this manuscript.

Response to Referee 1 -- NCOMMS-26-011336A

Reviewer:

I am overall satisfied with the authors' response to my comments and would like to support publication of the manuscript in Nature Communications.

Response:

We sincerely thank the reviewer for the positive evaluation of our revised manuscript and for supporting its publication in Nature Communications. We are grateful for the reviewer's constructive comments during the review process, which have helped us improve the clarity and quality of the manuscript.

Response to Referee 2 -- NCOMMS-26-011336A

Reviewer:

The authors have revised the manuscript in accordance with the reviewers' comments. I only have two minor remarks regarding the revision:

Response:

We thank the reviewer for the careful reading of our revised manuscript and for recognizing that we have revised the manuscript in accordance with the reviewers' comments. We also appreciate the reviewer's two minor remarks, which have helped us further improve the manuscript and Supplementary Information.

Reviewer:

1. The present manuscript demonstrates the STV using a mirror-symmetry-breaking slab, which is closely related to the recent experimental work Light 15, 77 (2026).

Response:

We thank the reviewer for pointing out this relevant recent experimental work. In the revised manuscript, we have now cited this paper.

Reviewer:

2. There is a typo in Fig. S4: the bottom label "Port u" should be "Port d".

Response:

We thank the reviewer for carefully identifying this typo. We have corrected the bottom label in Fig. S4 from "Port u" to "Port d" in the revised Supplementary Information.

Response to Referee 3 -- NCOMMS-26-011336A

Reviewer:

The authors have thoroughly revised the manuscript and have satisfactorily addressed the concerns raised in my first-round report, which was approximately 1900 words long. In addition, they have provided a very detailed and highly informative 49-page rebuttal letter, in which they carefully respond to the comments and questions of all three referees.

The revised manuscript represents a significant improvement over the original submission. The presentation is clearer, the discussion is better structured, and the main results are now more accessible to the reader. The authors have substantially strengthened the discussion of the novelty of their approach and its advantages over existing methods or even definitions of exceptional points (to mention, e.g., Liouvillian quantum exceptional points), thereby making the scientific significance and potential impact of the work much more evident.

As an example, I would like to mention my suggestion to make the study more complete by analyzing exceptional points associated with additional submatrices obtained by projecting the unitary scattering matrix onto lower-dimensional subspaces. This comment has been taken seriously by the authors, who have included a substantial amount of new material in the Supplementary Information. A similar request was also raised by another referee, and the authors have responded comprehensively by significantly extending both their analysis and discussion.

Although I have not independently verified every technical aspect of the analysis in full detail, the results appear to be physically sound and internally consistent. Moreover, the authors' responses to the referees' comments are thoughtful, comprehensive, and convincing.

In view of these improvements, I believe that the manuscript meets the high standards of Nature Communications, and I am pleased to recommend its publication.

Response:

We sincerely thank the reviewer for the thoughtful and generous assessment of our revised manuscript. The reviewer's constructive comments have significantly strengthened the manuscript, and we are grateful for the recommendation for publication in Nature Communications.
